# Revisions and key to the Vernonieae (Compositae) of Thailand

**DOI:** 10.3897/phytokeys.37.6499

**Published:** 2014-05-13

**Authors:** Sukhonthip Bunwong, Pranom Chantaranothai, Sterling C. Keeley

**Affiliations:** 1 Maejo University Phrae Campus, Mae Sai, Rong Kwang, Phrae 54140, Thailand; 2 Applied Taxonomic Research Center, Department of Biology, Faculty of Science, Khon Kaen University, Khon Kaen 40002, Thailand; 3 Department of Botany, University of Hawaii, Honolulu, HI 96816 USA

**Keywords:** *Acilepis*, Asteraceae, *Camchaya*, Cichorioideae, *Cyanthillium*, *Decaneuropsis*, *Elephantopus*, *Ethulia*, *Gymnanthemum*, *Iodocephalopsis*, *Koyamasia*, *Kurziella*, *Monosis*, *Okia*, *Pseudelephantopus*, *Pulicarioidea*, southeast Asia, *Strobocalyx*, *Struchium*, *Tarlmounia*

## Abstract

Seventeen genera and 48 species, in five subtribes, are recognized in Thailand. These include 15 endemic taxa, half of which are in the largest genus, *Acilepis*, with others in the genera *Camchaya*, *Koyamasia*, and *Okia.* A new monotypic genus, *Pulicarioidea*, is established with *P. annamica*, the new name for the species formerly known as *Vernonia pulicarioides*. New combinations are also made for *Acilepis kerrii*, *Cyanthillium montanum*, *Koyamasia curtisii* and *Okia pseudobirmanica*. Forty-six characters including habit, leaf, flower, achene and pollen morphology were analyzed using UPGMA. Five clusters of taxa were identified. Keys to genera, species and varieties, descriptions, vernacular names, ecological data and illustrations are provided.

## Overview of the Vernonieae and taxonomic history of the tribe in Thailand

The Vernonieae have been shown to be unequivocally monophyletic and are well represented in both the Old and New Worlds, with centers of diversity in east Africa and Brazil (Keeley et al. 2007, Keeley and Robinson 2009). Recent revisions by Robinson (1999a, b, 2007) based on morphology, secondary chemistry and palynological studies, combined with data from the molecular work of Keeley et al. (2007) and Keeley and Robinson (2009), confirm a division into two separate lineages, one for the Old World and one for the New World taxa. Within these lineages Keeley and Robinson (2009) recognized six Old World and 14 New World subtribes and a total of 125 genera. Additional studies since that publication have added to this number, particularly in the Old World (e.g., Bunwong et al. 2009, Robinson and Skvarla 2006, 2009). The range and circumscription of the type genus, *Vernonia*, was also greatly changed by Robinson’s 1999 studies. Once thought to be distributed worldwide and to contain >1000 species, *Vernonia* is now confined to fewer than 25 species and restricted to the Americas. Name changes are not complete for Old World species formerly ascribed to this genus, however, and the generic status of these species will certainly change in the future when these larger scale studies are completed.

Koyama’s publications of Thai Vernonieae (1984, 1993, 1997, 1998, 2003, 2004, 2005) included descriptions of all species known at that time, and also included descriptions of several new genera. Over the course of this work Koyama also synonymized taxa given in the earlier treatments of Kerr (1936) and Suvatti (1978), the only other workers to specifically address the Vernonieae in Thailand. These latter authors held widely differing concepts regarding the number of Vernonieae species as well as conflicting generic concepts (i.e. 16 genera and 196 species, and two genera and 11 species, respectively). Koyama recognized a total of 44 species, most of which were placed in the traditionally large core genus *Vernonia* *s.l.*; only four other genera, *Camchaya*, *Elephantopus*, *Ethulia*, and *Struchium*, were recognized in his treatments. However, only the former genus is southeast Asian with its species native to Thailand, the latter three are found throughout tropical areas of both the New and Old Worlds.

The largest concentration of endemic Vernonieae is found in the northern and northeastern regions of Thailand, and outside of the country’s political boundaries in the adjacent regions of Burma and Yunnan, China. Taxa are typically found in open areas within dipterocarp, deciduous, evergreen and pine-oak forests, at elevations from sea level to over 2000 m. There are 15 endemics: *Acilepis chiangdaoensis*, *Acilepis doichangensis*, *Acilepis kerrii*, *Acilepis namnaoensis*, *Acilepis ngaoensis*, *Acilepis principis*, *Acilepis pseudosutepensis*, *Acilepis sutepensis*, *Camchaya pentagona*, *Camchaya spinulifera*, *Camchaya tenuiflora*, *Camchaya thailandica*, *Koyamasia calcarea*, *Koyamasia curtisii* var. *tomentosa*, and *Okia pseudobirmanica* (treated as species of *Vernonia* by Koyama 1984, 1993, 1997, 1998, 2003, 2004, 2005). Of these, five are restricted to limestone substrates in evergreen forests (*Acilepis pseudosutepensis*, *Koyamasia calcarea*, *Koyamasia curtisii* var. *tomentosa*, *Okia birmanica* and *Okia pseudobirmanica*). *Cyanthillium cinereum*, *Elephantopus scaber*, *Elephantopus mollis*, *Ethulia conyzoides*, *Pseudelephantopus spicatus*, and *Struchium sparganophorum* are found widely throughout the tropics worldwide. Non-weedy, but also widespread in the Malay Peninsula are *Cyanthillium patulum*, *Decaneuropsis cumingiana*, *Strobocalyx arborea*, *Cyanthillium montanum* and *Koyamasia curtisii*.

Koyama’s treatments were used as the starting points for this study, with modifications in generic assignment by Robinson (1999a, b, 2007), Bunwong et al. (2009), and Robinson and Skvarla (2009). Koyama did not consider subtribes, so these are also assigned according to the work of Robinson (1999a, 2007). The relationships among Vernonieae taxa of many south and southeast Asian, Indo-Chinese and Malaysian Vernonieae lack any kind of treatment beyond the earliest naming and description and hence have not been included in this study. Additionally, there are > 200 species in this region which fact has also made understanding of the relationships among these taxa extremely challenging. The present study provides a revision of the genera and species, with keys and complete descriptions for all Vernonieae found within Thailand.

## Materials and methods

### Specimens and morphological measurements

Herbarium specimens were obtained from AAU, B, BCU, BK, BKF, BM, C, E, G, K, KKU, L, P, PSU, QBG and US. Field collections were also made throughout Thailand by the first author and have been deposited at KKU, QBG and US. Information recorded from specimens included distribution, and when available ecological data and vernacular name(s). Only mature vegetative and reproductive parts were measured or scored. Macromorphological measurements, presence/absence and specifics of surfaces and vestitures were obtained by light microscope. Achenes, leaf surfaces and unacetolyzed pollen were observed with scanning electron microscopy (SEM) using a LEO, 1450VP. A complete listing of taxa and voucher information are given in Table 1.

**Table 1.** Taxa and voucher information for species included in UPGMA analysis.

Species	Locality (Province)	Voucher information
*Acilepis attenuata* (I)	Udon Thani	S. Bunwong 347
*Acilepis attenuata* (II)	Khon Kaen	S. Bunwong 351
*Acilepis attenuata* (III)	Loei	S. Bunwong 373
*Acilepis attenuata* (IV)	Sakon Nakon	S. Bunwong 354
*Acilepis chiangdaoensis*	Chiang Mai	S. Bunwong 78
*Acilepis divergens* (I)	Chiang Mai	S. Bunwong 366
*Acilepis divergens* (II)	Ciang Mai	S. Bunwong 377
*Acilepis kingii*	Chiang Mai	S. Bunwong 77
*Acilepis namnaoensis*	Chaiyaphum	S. Bunwong 385
*Acilepis ngaoensis*	Ranong	S. Bunwong 386
*Acilepis peguensis*	Loei	S. Bunwong 372
*Acilepis pseudosutepensis*	Tak	S. Bunwong 388
*Acilepis saligna*	Mae Hong Son	S. Bunwong 357
*Acilepis silhetensis*	Chiang Mai	S. Bunwong 364
*Acilepis sutepensis* (I)	Chiang Mai	S. Bunwong 361
*Acilepis sutepensis* (II)	Chiang Mai	S. Bunwong 367
*Camchaya gracilis*	Ubon Ratchathani	S. Bunwong 346
*Camchaya loloana* (I)	Khon Kaen	S. Bunwong 330
*Camchaya loloana* (II)	Ubon Ratchathani	S. Bunwong 339
*Camchaya loloana* var. *mukdahanensis* (I)	Mukdahan	S. Bunwong 338
*Camchaya loloana* var. *mukdahanensis* (II)	Ubon Ratchathani	S. Bunwong 343
*Camchaya pentagona*	Ubon Ratchathani	S. Bunwong 344
*Camchaya spinulifera* (I)	Udon Thani	S. Bunwong 327
*Camchaya spinulifera* (II)	Sakon Nakon	S. Bunwong 332
*Camchaya spinulifera* (III)	Nong Khai	S. Bunwong 336
*Camchaya tenuiflora*	Loei	S. Bunwong 348
*Camchaya thailandica*	Udon Thani	S. Bunwong 328
*Cyanthillium cinereum*	Loei	S. Bunwong 350
*Cyanthillium hookerianum*	Unon Ratchathani	S. Bunwong 341
*Cyanthillium montanum* (I)	Mae Hong Son	S. Bunwong 356
*Cyanthillium montanum* (II)	Chiang Mai	S. Bunwong 371
*Decaneuropsis cumingiana*	Petchaboon	S. Bunwong 74
*Decaneuropsis eberhardtii*	Chaiyaphum	S. Bunwong 384
*Decaneuropsis garrettiana*	Chiang Mai	S. Bunwong 75
*Elephantopus mollis*	Ubon Ratchathani	S. Bunwong 340
*Elephantopus scaber* (I)	Udon Thani	S. Bunwong 325
*Elephantopus scaber* (II)	Sakon Nakon	S. Bunwong 334
*Elephantopus scaber* var. *penicillatus*	Ubon Ratchathani	S. Bunwong 345
*Gymnanthemum extensum*	Chiang Mai	S. Bunwong 378
*Iodocephalopsis eberhardtii*	Chiang Mai	S. Bunwong 335
*Koyamasia calcarea*	Chiang Mai	P. Suksathan 2847
*Koyamasia curtisii*	Chiang Rai	S. Watthana 875
*Kurziella gymnoclada*	Khon Kaen	S. Bunwong 391
*Monosis volkameriifolia*	Chiang Mai	S. Bunwong 362
*Pseudelephantopus spicatus* (I)	Ubon Ratchathani	S. Bunwong 342
*Pseudelephantopus spicatus* (II)	Chiang Rai	S. Bunwong 352
*Strobocalyx arborea*	Loei	M. Norsangsri 1052
*Strobocalyx solanifolia*	Loei	S. Bunwong 395
*Tarlmounia elliptica* (I)	Khon Kaen	S. Bunwong 390
*Tarlmounia elliptica* (II)	Khon Kaen	S. Bunwong 392

### Phenetic analysis

**Figure 1.**
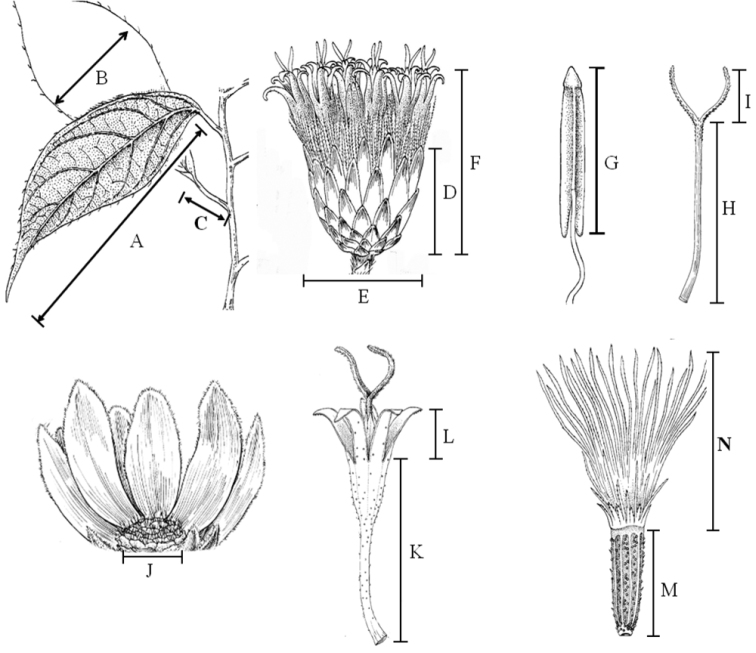
Illustration of characters used in morphological descriptions and UPGMA analysis. **A** Leave length **B** Leave width **C** Petiole length **D** Involucral length **E** Involucral diameter **F** Capitula height **G** Anther length **H** Style length **I** Stigma length **J** Receptacle diameter **K** corolla tubescorolla tube length **L** Corola lobe length **M** Achene length **N** Inner pappus length (Modified from Robinson 1999a)

**Figure 2.**
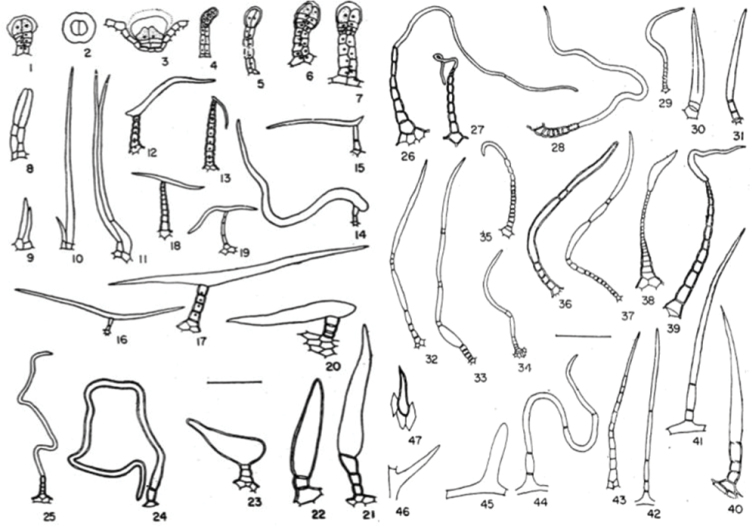
Vernonieae trichome types used in morphological descriptions and UPGMA analysis. **1–7** Glandular **8–1.** Biseriate or twin **12–15** One armed **16–19** T-shaped **20–23** Swollen terminal celled **24–25** Flagellate **26–28** Whip-shaped **29–31** and **45** Cylindrical **32–44** Filiform **46–47** Prickly (Applied from Narayana 1979).

**Figure 3.**
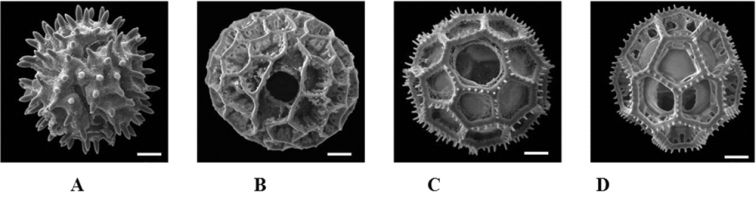
Representative pollen types of Vernonieae. **A** Echinate 3-colporate **B** Lophate 3- colporate **C** Lophate 3-porate **D** Lophate 6-porate (Applied from Bunwong and Chantaranothai 2008).

Twenty-six binary and 20 multistate characters were obtained for 42 species (Table 1, 2), morphological characters are shown in Figs 1, 2, and representative pollen types in Fig. 3. At least 15 specimens per taxon were measured and scored. Data for some taxa were already available (Bunwong and Chantaranothai 2008) and were used as recorded in that study as the methodology was the same as that used here. All data were prepared using MacClade 4.03 (Maddison and Maddison 2001) and imported into PAUP* 4.0b10 (Swofford 2002) for Unweighted Pair Group Mathematical Average (UPGMA) cluster analysis. UPGMA tree were constructed using distance mesure of mean character difference. The number of 1000 replicates is used for all bootstrap tests.

**Table 2.** Morphological characters and character states included in this study.

No.	Characters and character states
1	Habit; erect herb (0), scandent (1), shrub (2), tree (3)
2	Stem; acauline (0), cauline (1)
3	Stem rib; absent (0), present (1)
4	Stem vestiture; puberulous (0), tomentose (1), villose-pilose (2), sericeous (3)
5	Petiole length; 0-1 mm (0), > 1 mm (1)
6	Leaf shape; ovate or lanceolate (0), obovate or oblanceolate (1), elliptic or oblong (2)
7	Leaf margin; crenate (0), serrate (1), entire or undulate (2)
8	Leaf apex; obtuse or rounded (0), acute or acuminate (1), apiculate or cuspidate (2), caudate or aristate (3)
9	Leaf base; attenuate (0), cuneate or obtuse (1)
10	Leaf texture; coriaceous (0), chartaceous (1)
11	Leaf trichomes: whip-shaped with long terminal cell; absent (0), present (1)
12	Leaf trichomes: whip-shaped with short terminal cell; absent (0), present (1)
13	Leaf trichomes: filiform; absent (0), present (1)
14	Leaf trichomes: flagellate; absent (0), present (1)
15	Leaf trichomes: cylindrical; absent (0), present (1)
16	Leaf trichomes: T-shaped; absent (0), present (1)
17	Vestiture on upper leaf surface; puberulous (0), tomentose (1), villose-pilose (2), sericeous (3), scabrous (4)
18	Vestiture on lower leaf surface; puberulous (0), tomentose (1), villose-pilose (2), sericeous (3), scabrous (4)
19	Gland on upper leaf surface; absent (0), present (1)
20	Gland on lower leaf surface; absent (0), present (1)
21	Capitulescence type; spicate (0), paniculate (1), solitary (2), scapose (3), corymbose (4)
22	Phyllary rows; 1-2 (0), 3-5 (1), > 5 (2)
23	Phyllary arrangement; decussate (0), imbricate (1)
24	The middle phyllary shape; ovate (0), lanceolate (1), oblong (2)
25	The outer and the middle phyllary apex; obtuse (0), acute or acuminate (1), apiculate or aristate (2), spinose (3)
26	The outer and the middle phyllary with reflexed apex; absent (0), present (1)
27	Phyllary margin; ciliate (0), piliferous (1), spinulose (2), entire (3)
28	Phyllary vestiture; puberulous (0), tomentose (1), villose-pilose (2), arachnoid (3), sericeous (4)
29	Capitate gland on phyllaries; absent (0), present (1)
30	Number of floret per a capitulescence; 1-4 (0), 5-30 (1), >30 (2)
31	Corolla symmetry; actinomorphic (0), zygomorphic (1)
33	Corolla trichomes; absent (0), present (1)
34	Pollen type; echinate (0), sublophate (1), lophate (2)
35	Pollen furrow; absent (0), present (1)
36	Pollen pore shape; circular (0), semicircular (1)
37	Number of pollen pore; 3 pores (0), 6 pores (1)
38	Pollen spine length; 0 µm (0), >0-5 µm (1), >5 µm (2)
39	Pollen columella; prominent (0), inconspicuous (1)
40	Pollen micropuncta; absent (0), present (1)
41	Achene shape; turbinate (0), clavate (1), terete (2)
42	Achene trichomes; absent (0), present (1)
43	Achene glands; absent (0), present (1)
44	Number of achene rib; 1-9 (0), ≥10 (1)
45	Carpopodium; absent (0), present (1)
46	Pappus; absent (0), present in every floret (1), present in some florets (2)

## Results and discussion

**Figure 4.**
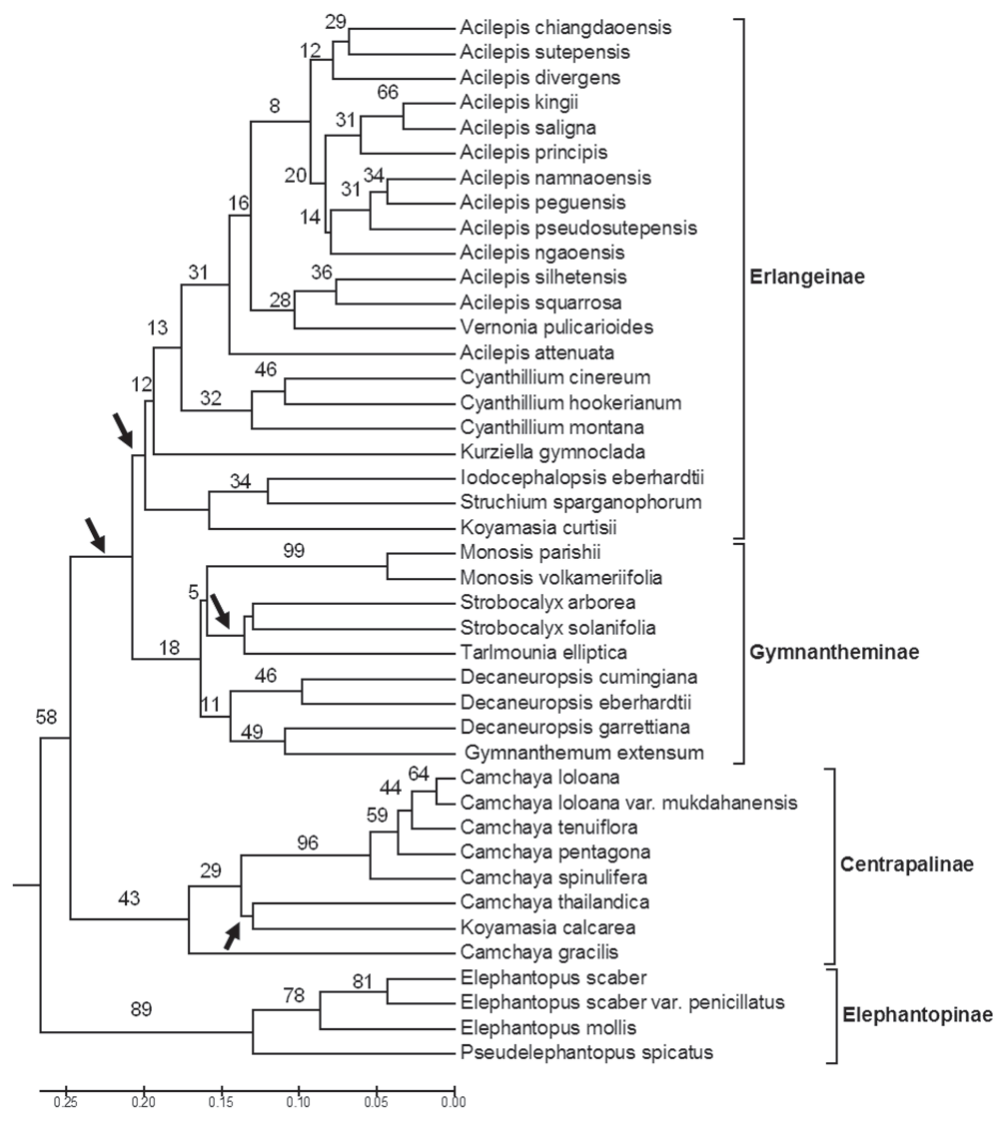
UPGMA tree for 42 Thai Vernonieae based on vegetative, reproductive and pollen morphology (see Table 2, Figs 1, 2, 3). Number above the lines indicate bootstrap support (1000 replications). Zero length branches indicated by arrows.

Five clusters of taxa are recognized in the UPGMA analysis as shown in Fig. 4. These groups largely correspond to three Old World subtribes recognized by Robinson (1999b), the Centrapalinae, Erlangeinae, Gymnantheminae, and two adventive New World subtribes, Elephantopinae, and Vernoniinae. In addition, seven taxa, *Cyanthillium montanum*, *Kurziella gymnoclada*, *Okia birmanica*, *Okia pseudobirmanica*, *Koyamasia curtisii*, *Koyamasia curtisii* var. *tomentosa*, and *Pulicarioidea annamica* not previously assigned to subtribe, appear to belong to the Erlangeinae. However, this assignment is considered tentative as further work will be needed to show definitive placement of these species.

*Camchaya* is a well-supported genus within the subtribe Centrapalinae (Fig. 4). It is characterized by an annual habit, erect, leafy stems, achenes without a carpopodium, a deciduous pappus, and echinolophate hexaporate pollen. Bunwong et al. (2009) distinguished the genus *Iodocephalopsis* from *Camchaya* based on the absence of a spine at the margin of the involucral bracts, differences in bract shape, and the former taxon’s tricolporate pollen. Although both genera are clearly closely related, their placement within the subtribe Centrapalinae may be subject to change. Robinson (pers. comm.) expressed doubt about the existence of true Centrapalinae in Thailand because the taxa are morphologically distinct from those in this same subtribe in Africa, the taxa for which the subtribe was originally described. As a result, the position of Thai taxa now ascribed to this subtribe will need to be evaluated within a larger analysis that also includes African Centrapalinae taxa before a final decision can be made.

Structural features including pollen place the Asian *Gymnanthemum* and *Decaneuropsis* clearly in the subtribe Gymnantheminae, but DNA sequencing is still needed for confirmation. The place of *Monosis* is less certain. For example, genera such as *Decaneuropsis* (and possibly *Strobocalyx* and *Tarlmounia*) have been assigned to the Gymnantheminae (i.e. Robinson 2007, Robinson and Skvarla 2007, Robinson et al. 2008, 2010) while others have been removed and placed in their own subtribe, i.e., *Distephanus*, now in subtribe Distephaninae (Keeley and Robinson 2009). Typical African Gymnantheminae differ in habit from their Thai counterparts as well. African species are typically low-growing scandent shrubs while southeast Asian taxa are large shrubs and treelets. Taxa also differ in corolla morphology and in inflorescence type, characteristically thrysiform in African taxa rather than corymbiform as in the Thai species (Robinson and Skvarla 2007). On the other hand, both African and southeast Asian taxa now placed in this subtribe share deciduous involucral bracts, blunt sweeping hairs on the style, an indurate appendage on the anthers, reflexed and deeply divided corolla lobes, and tricolporate, non-lophate pollen (Robinson 2007, Robinson et al. 2008, Robinson and Skvarla 2009). Whether the Thai genera should be placed in a new subtribe separate from the African Gymnantheminae will require a more detailed study that includes putative members of the subtribe from across its geographical range.

A similar situation also appears to exist in the subtribe Erlangeinae which, like the Gymnantheminae and Centrapalinae, is primarily African (Keeley and Robinson 2009). The six Thai endemic *Acilepis* species, as well as *Kurziella gymnoclada*, now assigned to the Erlangeinae, differ from their African relatives in both reproductive and morphological characters. Also, as is the case for the other subtribes in this study, no African taxa were included in the analyses and, as a result, relationships to other members of this subtribe, including the type genus *Erlangea*, are unknown. Additionally, other taxonomic changes may also be necessary to create monophyletic genera. Further, the subgroup composed of *Iodocephalopsis*, *Struchium* and *Koyamasia curtisii* also needs a more thorough evaluation. Robinson (1999a, b) placed *Struchium* in the primarily New World subtribe Lepidaploinae, and its putative alliance with Old World taxa, as seen here (Fig. 4), may be due to morphological convergence rather than to a close genetic relationship. Until such a time when a wider range of Erlangeinae taxa can be included in an analysis with Thai species, the subtribal status and the relationships among this threesome will remain unclear.

Unlike the Centrapalinae, Erlangeinae, and Gymnantheminae described above, however, the membership of taxa in the Elephantopinae is clear (Fig. 4). Additionally, this subtribe has been previously shown to be monophyletic using DNA sequence data (Keeley et al. 2007). Species in this subtribe are annual to biennial herbs or subshrubs with liguliform zygomorphic corollas, capitula clustered within foliose bracts, filiform hairs on the leaf surfaces and echinolophate triporate pollen, a distinctive combination of characters that clearly sets this subtribe off from the rest of the Vernonieae. Keeley et al. (2007) also found that the Elephantopinae are New World in origin, and *Elephantopus mollis*, *Elephantopus scaber* and *Pseudelephantopus spicatus* now found in disturbed locations throughout the tropics has achieved this distribution by following in the wake of people and their domestic animals. They are excellent camp followers.

## Conclusions

There is now a taxonomic framework that will allow for future development of and testing of hypotheses of Vernonieae relationships over a wide region of the Old World including Africa and south and southeast Asia. Of particular interest are the relationships of taxa now putatively in the same subtribe but whose characteristics differ (i.e., between Asia and Africa). Thailand may also be key to understanding subtribal radiations across the Old World as it is located at the crossroads of biotic migrations westward from Malaysia and eastward from India and Africa. Understanding the subtribal histories will allow us to follow the historical pathways of dispersal, and identify habitat types that promote endemism and local adaptive radiations. The Vernonieae has been referred to as the “evil tribe” (Keeley et al. 2007) because of its taxonomically refractory nature. It has always been difficult to make clear distinctions at every level from the most inclusive subtribe and genus (i.e., *Vernonia*) to the individual species. However, the Vernonieae is also one of the few plant groups blessed with the right combination of geographical distribution, and diversity of genera and species to make it possible to gain a good picture of plant evolution across a region of rich biotic diversity, such as is found in Thailand and southeast Asia.

A key to the genera and species within each genus is provided below. Subtribes are shown in Fig. 4. It is hoped that the reader will use this to gain greater understanding of the tribe in Thailand, and that it will spur further work on this beautiful and challenging group.

### Vernonieae

Cassini, J. Phys. Chim. Hist. Nat. Arts 82: 132. 1816.

urn:lsid:ipni.org:names:235016-2:1.3

http://species-id.net/wiki/Vernonieae

Serratula noveboracensis L., Sp. Pl.: 818. 1753.

#### Type.

*Vernonia noveboracensis* (L.) Willd., Sp. Pl., ed. 4, 3: 1632. 1803.

Herbs, shrubs, vines or trees. Stems acaulescent or caulescent. Leaves simple, usually alternate sometimes opposite or ternate, petiolate or sessile; lamina ovate, obovate, lanceolate, oblanceolate or elliptic, pubescent, margin serrate or entire, apex rounded, acute or acuminate, base cuneate or attenuate, membranaceous, chartaceous or coriaceous. Capitulescences terminal or axillary, solitary, paniculate or corymbiform with cymose branches, sometimes spicate. Capitula discoid, homogamous, pedunculate or sessile, florets bisexual and fertile. Involucres with numerous, imbricate, persistent bracts. Florets purple to white, actinomorphic or zygomorphic, lobes 3–5, pubescent or glabrous. Anthers usually 5, purple to yellow or white, syngenesious bases usually calcarate. Styles purple to white, 2-branched, inner surface covered with stigmatic papillae, outer surface covered with sweeping hairs. Achenes subterete, clavate or obovate, 3–10-ribbed, carpopodium present or absent. Pappus present or absent; if present bristly or coroniform, in 1–2 series, persistent or deciduous. Pollen echinate or lophate, 3-porate, 6-porate or 3-colporate, with or without micropuncta.

#### Key to the genera

**Table:** 

1	Corolla strongly zygomorphic, liguliform with single deepest sinus	2
–	Corolla actinomorphic, without single deepest sinus	3
2	Pappus of straight bristles	*Elephantopus*
–	Pappus of contorted bristles	*Pseudelephantopus*
3	Pappus absent	4
–	Pappus present	5
4	Achenes with 7–10 ribs; pollen lophate and sub-3-colporate	*Iodocephalopsis*
–	Achenes with 4–6 ribs; pollen non-lophate	*Ethulia*
5	Corolla 3- or 4-lobed; capitula appearing sessile; pappus thick and coroniform	*Struchium*
–	Corolla 5-lobed; capitula mostly pedunculate; pappus of bristles	6
6	Vines, scandent shrubs or trees	7
–	Herbs or subshrubs	11
7	Achenes less than 2.5 mm long; involucres less than 4 mm long; pollen not or weakly sublophate, with continuous perforated tectum between colpi	8
–	Achenes more than 2.5 mm long; involucre more than 4 mm long; pollen lophate, with or without continuous perforated tectum between colpi	9
8	Achenes glabrous; involucre glanduliferous; stems and leaves sericeous with long-armed T-shaped trichomes	*Tarlmounia*
–	Achenes pubescent; involucre eglanduliferous; leaves and stems not sericeous with T-shaped trichomes	*Strobocalyx*
9	Involucres 7–10 mm long; pollen psilolophate with high muri	*Monosis*
–	Involucres 4–5 mm long; pollen strongly echinate	10
10	Small trees or shrubs; corolla tubes broad, not closely investing the style shaft	*Gymnanthemum*
–	Scandent shrubs; corolla tubes slender, closely investing style shaft	*Decaneuropsis*
11	Pappus in one series, without strongly differentiated outer series	12
–	Pappus in 2 series	14
12	Plants leafless at anthesis; pappus persistent; pollen echinate, 3-colporate	*Kurziella*
–	Plants with persistent leaves; pappus caduceus; pollen lophate	13
13	Achenes obovate, without evident carpopodia; phyllaries not reflexed; pollen 6-porate	*Camchaya*
–	Achenes oblong; phyllaries reflexed; pollen 3-porate	*Koyamasia*
14	Pappus of flattened setae	*Pulicarioidea*
–	Pappus of capillary bristles not dilated at the base	15
15	Leaves and stems with T-shaped hairs	*Cyanthillium*
–	Leaves and stems without T-shaped hairs	16
16	Peduncles fistulose, 25–40 cm long	*Okia*
–	Peduncles not fistulose, mostly less than 15 cm long	*Acilepis*

### Acilepis

D.Don, Prodr. Fl. Nepal: 169. 1825.

urn:lsid:ipni.org:names:174526-1:1.3

http://species-id.net/wiki/Acilepis

Lysistemma Steetz, Naturw. Reise Mossambique [Peters] 6(Bot., 2): 340. 1864.
Xipholepis Steetz, Naturw. Reise Mossambique [Peters] 6(Bot., 2): 344. 1864.

#### Type.

*Acilepis squarrosa* D.Don, Prodr. Fl. Nepal. 169. 1825.

Perennial herbs. Stems acaulescent or caulescent. Leaves simple, alternate or rosulate, petiolate, lamina ovate, obovate or elliptic, pubescent, margin serrate or entire, apex rounded, acute or acuminate, base cuneate or attenuate, subcoriaceous. Capitulescences terminal or axillary, corymbose, paniculate, spiciform, scapose, solitary. Capitula discoid, homogamous, pedunculate or sessile, florets bisexual and fertile. Involucres herbaceous. Florets 6–80; corollas purple to white, actinomorphic, lobes 5. Anthers 5. Styles purple, 2-branched, inner surface covered with stigmatic papillae, outer surface covered with sweeping hairs reaching to below style bifurcation. Achenes subterete or obovate, usually 10-ribbed, carpopodium present. Pappus in 2 series of bristles, persistent, the outer ones are shorter than the inner ones. Pollen lophate, 3-porate, without micropuncta.

Sixteen species are recognized in Thailand.

#### Key to the species of *Acilepis* in Thailand

**Table:** 

1	Capitula with more than 30 florets	2
–	Capitula with less than 30 florets	6
2	Phyllaries mostly reflexed	3
–	Phyllaries not reflexed	4
3	Leaves basal; corolla and achenes pubescent	*Acilepis attenuata*
–	Leaves cauline; corollas and achenes without pubescence	*Acilepis silhetensis*
4	Capitula terminal, pedunculate	5
–	Capitula axillary, sessile	*Acilepis squarrosa*
5	Phyllaries with dense tomentum; capitula subsessile	*Acilepis doichangensis*
–	Phyllaries with sparsely arachnoid hairs; peduncles elongate	*Acilepis ngaoensis*
6	Capitula pedunculate, involucre ≥ 5 mm long	7
–	Capitula subsessile, involucre ≤ 5 mm long	*Acilepis divergens*
7	Phyllaries herbaceous, apex mucronate or aristate	8
–	Phyllaries scarious, spinose	*Acilepis chiangdaoensis*
8	Leaves chartaceous; achenes pubescent	9
–	Leaves membranaceous; achenes lacking trichomes	*Acilepis peguensis*
9	Upper leaves elliptic, acute to acuminate; achenes glandular	10
–	Upper leaves linear-lanceolate to falcate, or caudate; achenes eglandular	*Acilepis principis*
10	Leaves subsessile or with petioles up to 6 mm long	11
–	Leaves petiolate, petioles up to 25 mm long	13
11	Capitula with ≥ 20 florets, involucres cup-shaped or campanulate	12
–	Capitula with 8–12 florets, involucres narrowly campanulate	*Acilepis kingii*
12	Herbs 1–2 m tall; leaves 15–20 by 5–8 cm	*Acilepis namnaoensis*
–	Herbs 0.2–0.4 m tall; leaves 4–6 by 1–2.5	*Acilepis kerrii*
13	Corolla without hairs; achenes 2–3.5 mm long	14
–	Corolla with sparse hairs; achenes 4–5 mm long	*Acilepis saligna*
14	Inner pappus 8–9 mm long; involucres 8–10 mm long	15
–	Inner pappus 6–7 mm long; involucres 6–7 mm long	*Acilepis virgata*
15	Receptacle glabrous	16
–	Receptacle pubescent	*Acilepis tonkinensis*
16	Phyllaries and peduncles densely villose; pappus 6–7 mm long; anthers purple	*Acilepis pseudosutepensis*
–	Phyllaries and peduncles sparsely pilose; pappus 8–9 mm long; anthers yellowish	*Acilepis sutepensis*

### Acilepis attenuata

(DC.) H.Rob. & Skvarla, Proc. Biol. Soc. Washington 122(2): 137. 2009.

urn:lsid:ipni.org:names:77114130-1:1.2

http://species-id.net/wiki/Acilepis_attenuata

Conyza attenuata Wall., Numer. List [Wallich] no. 3020, comp. no. 130, *nom. nud.*
Vernonia attenuata DC., Prodr. 5: 33. 1836.

#### Type.

**Figure 5.**
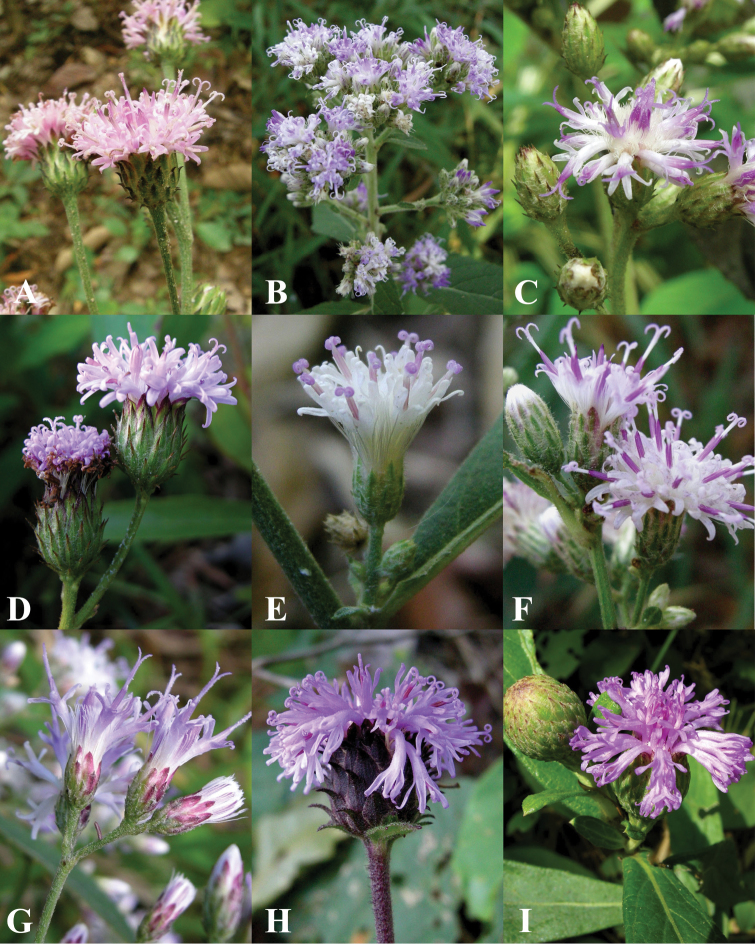
Morphology of *Vernonieae* in Thailand 1. **A** *Acilepis attenuata* **B** *Acilepis divergens* **C** *Acilepis namnaoensis* **D** *Acilepis ngaoensis* **E** *Acilepis peguensis* **F** *Acilepis principis* **G** *Acilepis saligna* **H** *Acilepis silhetensis* **I** *Acilepis squarrosa*.

India orientalis, *Wallich* 3020 (holotype: K!). Fig. 5A.

#### Description.

Perennial herbs 50–120 cm tall. Stems erect, conspicuously ribbed, puberulous. Leaves simple, rosulate, 9–22 by 2–8 cm, obovate to obovate-lanceolate or elliptic, margin entire or serrate, apex obtuse or acute, base cuneate or attenuate, subcoriaceous, upper surface scabrous and lacking glands; lower surface scabrous with whip-shaped hairs and capitate glands; lateral veins 5–11-paired; petioles up to 5 mm long. Capitulescences terminal, scapose. Capitula campanulate, 15–18 mm long, pedunculate. Receptacle flat, 6–8 mm in diam., glabrous. Involucres campanulate, in 6–7 series, 7–11 mm long, herbaceous. Phyllaries imbricate, green with purple apex, margin piliferous, outer surface arachnoid without glands; the outer and the middle ones ovate, apex apiculate, upper half reflexed; the inner ones ovate-lanceolate to oblong, apex acuminate. Florets 40–65; corollas funnelform, purple, puberulous glandular; corolla tubes 6–8.5 mm long; corolla lobes 2–3 mm long. Anthers 2.5–3 mm long, apical appendage acute, base obtuse. Styles purple, 7–10 mm long, branches 2.5–4 mm long. Achenes subterete, 2.5–3 mm long, 10-ribbed, pubescent with twin hairs and capitate glands. Pappus bristles, the inner ones 7–7.5 mm long.

#### Distribution.

Thailand: Mae Hong Son, Chiang Mai, Nong Bua Lum Phu, Khon Kaen, Udon Thani, Sakon Nakhon, Kanchanaburi, Satun. Myanmar.

#### Specimens examined.

Thailand. Loei: Phu Kra Dung national park, 16°52.28'N, 101°50.74'E, 23 Dec 2007, *S. Bunwong* 373 (KKU, US); Udon Thani, Phu Pra Bath historical park, 17°41.30'N, 102°28.40'E, 2 Nov 2007, *S. Bunwong* 347 (KKU, US); Khon Kaen, Phu Wieng national park, 15 Nov 2007, *S. Bunwong* 351 (KKU, US); Sakon Nakhon, Phu Phan national park, 24 Nov 2007, *S. Bunwong* 354 (KKU, US); Satun, Adang Island, 21 Oct 1979, *G. Congdon* 62 (AAU, PSU); Mae Hong Son, Pang Ma Pha, 25 Feb 1968, *B. Hansen & T. Smitinand* 12722 (C, E, K, L, P); Kanchanaburi, Si Sa Wat, 5 Jan 1926, *A.F.G. Kerr* 10147 (BK, BM, K).

#### Ecology.

Dipterocarp or dry evergreen forest, alt. 50–780 m; flowering October to February.

#### Diagnostic characters.

Leaves rosulate and capitulescences terminal with scapose.

#### Vernacular name.

Kra Dum Muang (กระดุมม่วง).

### Acilepis chiangdaoensis

(H.Koyama) H.Rob. & Skvarla, Proc. Biol. Soc. Washington 122(2): 140. 2009.

urn:lsid:ipni.org:names:77114132-1:1.2

http://species-id.net/wiki/Acilepis_chiangdaoensis

Vernonia chiangdaoensis H.Koyama, Bull. Natl. Sci. Mus. Tokyo, Ser. B 31(2): 69. 2005.

#### Type.

Thailand, Chiang Mai, Doi Chiangdao, *M. Tagawa, K. Iwatsuki & N. Fukuoka* T-4038 (holotype: KYO!).

#### Description.

Perennial herbs, 1–2 m tall. Stems erect, conspicuously ribbed, villose. Leaves 10–30 by 4–10 cm, lanceolate, margin serrate, apex acuminate, base attenuate, subcoriaceous, upper surface scabrous and without glands; lower surface scabrous with whip-shaped hairs, cylindrical hairs and capitate glands; lateral veins 7–10-paired; petioles up to 3 cm long. Capitulescences terminal, paniculate. Capitula campanulate, 10–12 mm long, pedunculate. Receptacle flat, 2–2.5 mm in diam., pubescent. Involucres campanulate in 4–5 series, 8–10 mm long, scarious.Phyllaries imbricate, green or purple apically, margin piliferous, outer surface villose, glands capitate; the outer and the middle ones lanceolate, apex spinose; the inner lanceolate to oblong, apex acuminate. Florets 10–15, narrowly funnelform, purple or white, glandular; corolla tubes 4–5 mm long; corolla lobes 2.5–3 mm long. Anthers 2.5–2.8 mm long, apical appendage acute, base obtuse. Styles purple, 4.5–6 mm long, branches 2.5–3 mm long. Achenes subterete, 2.5–3.5 mm long, 10-ribbed, pubescent with twin hairs and capitate glands. Pappus bristles, the inner ones 6–6.5 mm.

#### Distribution.

Thailand. Endemic.

#### Specimens examined.

Thailand. Chiang Mai, Chiangdao wildlife sanctuary, 19°26.08'N, 98°53.76'E, *S. Bunwong* 77 (KKU, US); Doi Chiangdao, 3 Jan 1966, *M. Tagawa, K. Iwatsuki & N. Fukuoka* T-4038 (KYO); Chiang Mai, Doi Sutep national park, 22 Dec 1989, *J.F. Maxwell* 89-1585 (CMU, L); Chiang Mai, Maerim, *W. Nanakorn et al.* 10191 (QBG); Wang Tao, *Th. Sørensen, K. Larsen & B. Hansen* 1073 (BKF, C, K).

#### Ecology.

Mixed deciduous or pine-oak forest, alt. 600–800 m; flowering December to February.

#### Diagnostic characters.

Large leaves and scarious spinose apical phyllaries.

#### Vernacular name.

Dok See Pa (ดอกสีป่า), DoK Doi Pa (ดอกดอยป่า).

### Acilepis divergens

(Edgew.) H.Rob. & Skvarla, Proc. Biol. Soc. Washington 122(2): 140. 2009.

urn:lsid:ipni.org:names:60456948-2:1.2

http://species-id.net/wiki/Acilepis_divergens

Conyza divergens Wall., Numer. List [Wallich] no. 3027A, comp. no. 137, *nom. nud.*
Eupatorium divergens Roxb., Fl. Ind. 3: 414. 1832.
Vernonia divergens (Roxb.) Edgew., J. Asiat. Soc. Bengal 2: 172. 1853.

#### Type.

India; *Wallich* 3027A (holotype: K!). Fig. 5B.

#### Description.

Robust herbs to subshrubs, 1–3 m tall. Stems erect, inconspicuously ribbed, pilose-villose or tomentose. Leaves 10–13 by 3–5 cm, ovate or elliptic, margin serrate, apex acute, base attenuate, subcoriaceous; both surfaces scabrous with whip-shaped hairs and capitate glands; lateral veins 6–10-paired; petioles up to 1 cm long. Capitulescences terminal and axillary, corymbose. Capitula narrowly campanulate, subsessile or shortly pedunculate, 9–10 mm long. Receptacle flat, ca. 1 mm in diam., glabrous. Involucres narrowly campanulate or slightly oblong-cylindrical, in 3–4 series, 4–5 mm long, 3–3.5 mm in diam., herbaceous. Phyllaries green or purple apically, margin piliferous, outer surface arachnoid, glandular; the outer and the middle ones ovate, acuminate or apiculate; the inner ones lanceolate to oblong, apex acuminate. Florets 6–10; corollas funnelform, purple, glandular; corolla tubes 4–5 mm long; corolla lobes 2–2.5 mm long. Anthers 2–2.5 mm long, apical appendage acute, base obtuse. Styles purple, 5–6 mm long, branches 1.5–2 mm long. Achenes obovate, ca. 2 mm long, 10-ribbed, glandular. Pappus bristles, the inner ones 4–5 mm long.

#### Distribution.

Thailand: Chiang Mai, Phayao, Nan, Tak, Sukhothai, Loei, Kanchanaburi. China (Yunnan), India, Myanmar, Laos, Myanmar, Vietnam.

#### Specimens examined.

Thailand. Chiang Mai, Doi Angkhang, 19°54.08'N, 99°2.34'E, 3 Jan 2008, *S. Bunwong* 377 (KKU, US); Chiang Mai, Doi Chiangdao, 29 Dec 1961, *K. Bunchuai* 102 (BKF, C, E, K, L, P); Doi Chiangdao, 6 Jan 1975, *R. Geesink, P. Hiepko & C. Phengklai* 8115 (BKF, C); Doi In Thanon, 12 Jan 1994, *N. Fukuoka & H. Koyama* T-62103 (BKF); Doi In Thanon, 9 Dec 1984, *H. Koyama*, *S. Mitsuta & T. Yahara* T-39916 (BKF); Doi In Thanon, 9 Dec 1984, *H. Koyama, S. Mitsuta & T. Yahara* T-48693 (BKF); Om Koi, 20 Jan 1964, *B. Hansen, G. Siedenfaden & T. Smitinand* 10796 (BKF, C, L); Doi Sutep, 24 Jan 1909, *A.F.G. Kerr* 524 (BM, K); Sa Moeng, 3 Feb 1913, *A.F.G. Kerr* 2918 (BM, K); Chom Thong, 9 Feb 1998, *F. Konta, C. Phengklai & S. Khao-iam* 4135 (BKF); Kanchanaburi, Don Ta Hom, 13 Apr 1965, *Adisai* 1046 (BK); Si Sa Wat, 16 Jan 1926, *A.F.G. Kerr* 10227 (BK, K, BM); Chiang Rai, 25 Dec 1993, *Charal* 431 (BKF); Tak, Doi Pae Poe, 15 Mar 1968, *B. Hansen & T. Smitinand* 12926 (BKF, C, E, K, L); Mae Sod, 26 Jan 1985, *Y. Paisooksantivatana* 1590-85 (BK).

#### Ecology.

Mixed deciduous and cloud forests, alt. 300–1850 m; flowering October to April.

#### Diagnostic characters.

*Acilepis divergens* is clearly distinguished by having a corymbose capitulescences with short peduncle and its capitula that are among the smallest in *Acilepis* spp.

#### Vernacular names.

San Ngern (สารเงิน), Nat Foi (หนาดฝอย).

### Acilepis doichangensis

(H.Koyama) H.Rob. & Skvarla, Proc. Biol. Soc. Washington 122(2): 140. 2009.

urn:lsid:ipni.org:names:77114133-1:1.2

http://species-id.net/wiki/Acilepis_doichangensis

Vernonia doichangensis H.Koyama, Bull. Natl. Sci. Mus. Tokyo, Ser. B 30(1): 22. 2004.

#### Type.

Thailand, Chiang Mai, Mae Taeng Diatrict, Doi Chang, *T. Shimizu, H. Toyokuni, H. Koyama, T. Yahara & T. Santisuk* T-20693 (holotype: KYO!).

#### Description.

Robust herbs, rhizomatous, 0.5–2 m tall. Stems erect, conspicuously ribbed, puberulose. Leaves 7–15 by 2–6 cm, elliptic or oblanceolate, margin subentire or remotely serrulate, apex acute or acuminate, base cuneate, subcoriaceous; both surfaces scabrous with hairs and capitate glands; lateral veins 8–10-paired; petioles up to 5 mm long. Capitulescences terminal and axillary, paniculate. Capitula campanulate, subsessile or pedunculate. Receptacle flat, glabrous. Involucres narrowly campanulate, in 5–6 series, 10–12 mm long, herbaceous. Phyllary margins piliferous, outer surface tomentose, glandular; the outer and the middle ones ovate, acuminate; the inner ones lanceolate to oblong, apex acute. Florets ca. 35; corollas funnelform, purple, glandular; corolla tubes 4–5 mm long; corolla lobes 4–5 mm long. Achenes oblong, ca. 5 mm long, 10-ribbed, glabrous. Pappus bristles, the inner ones ca. 8 mm long.

#### Distribution.

Thailand: Chiang Mai. Endemic.

#### Specimens examined.

Thailand. Chiang Mai, Mae Taeng Diatrict, Doi Chang, 19°7.32'N, 98°56.60'E, 17 Oct 1979, *T. Shimitzu, H. Toyokuni, H. Koyama, T. Yahara & T. Santisuk* T-19059 (KYO); Doi Chang, 24 Oct 1979, *T. Shimitzu, H. Toyokuni, H. Koyama, T. Yahara & T. Santisuk* T-20678 (KYO); Doi Chang, 24 Oct 1979, *T. Shimitzu, H. Toyokuni, H. Koyama, T. Yahara & T. Santisuk* T-20693 (KYO).

#### Ecology.

Hill evergreen forest, alt. 1000–1300 m; flowering September to January.

#### Diagnostic characters.

*Acilepis doichangensis* is distinguished from *Acilepis attenuata* and *Acilepis silhetensis* by its tomentose involucre and glabrous achenes.

#### Vernacular names.

Dok See Pa (ดอกสีป่า), DoK Doi Pa (ดอกดอยป่า).

### Acilepis kerrii

(Craib) Bunwong, Chantar. & S.C.Keeley comb. nov.

urn:lsid:ipni.org:names:77138473-1

http://species-id.net/wiki/Acilepis_kerrii

Vernonia kerrii Craib, Bull. Misc. Inform., Kew 1914: 7. 1914.

#### Type.

Thailand, Me Nan, Sop Ngao, rock crevices by river, *A.F.G. Kerr* 2404 (holotype: K!, isotype: BM!).

#### Description.

Perennial herbs, ca. 30 cm tall. Stems erect, basal branching, inconspicuously ribbed, puberulous. Leaves cauline, 4–6 by 1–2.5 cm, oblanceolate or elliptic, margin slightly serrate, apex acute or obtuse, base cuneate, chartaceous; both surfaces pubescent; lateral veins 7–10-paired; petioles up to 3 mm long. Capitulescences terminal or axillary, solitary or in loose panicled. Capitula campanulate, 10–11 mm long, subsessile or pedunculate. Involucres campanulate, in 4–5 series, 7–8 mm long. Phyllaries margin piliferous, outer surface nearly glabrous; the outer and the middle ones ovate, apex acute; the inner ones ovate-lanceolate, apex acute. Florets 20–25; corollas funnelform, purple, glandular, corolla tubes 6.5–7 mm long; corolla lobes 2–3 mm long. Achenes narrowly turbinate, ca. 3 mm long, 10-ribbed, puberulous. Pappus bristles, the inner ones 6–7 mm long.

#### Distribution.

Thailand: Chiang Mai, Nan. Endemic.

#### Specimens examined.

Thailand, Me Nan, Sop Ngao, 21 Feb 1912, *A.F.G. Kerr* 2404 (K, BM); Chiang Mai, Doi Fa Hom Pok national park, 20°2.72'N, 99°8.74'E, 24 Feb 1958, *Th. Sørensen, K. Larsen & B. Hansen* 1602 (K).

#### Diagnostic characters.

*Acilepis kerrii* can be distinguished by its subsessile capitula in axillary leaves.

#### Ecology.

Rock cliff by river in hill evergreen forest, alt. 700–1400 m; flowering February.

#### Vernacular name.

Muang Dong (ม่วงดง).

### Acilepis kingii

(C.B.Clarke) H.Rob. & Skvarla, Proc. Biol. Soc. Washington 122(2): 141. 2009.

urn:lsid:ipni.org:names:60456951-2:1.2

http://species-id.net/wiki/Acilepis_kingii

Acilepis kingii *Vernonia kingii* C.B.Clarke, Compos. Ind. 12. 1876.

#### Type.

Myanmar, Yomah province, Pegu, Irrawaddy & Sittang Valley, *S. Kurz* s.n. (holotype: K!).

#### Description.

Perennial herbs, 1–1.5 m tall. Stems erect, conspicuously ribbed, puberulous. Leaves 10–20 by 4–8 cm, lanceolate, margin serrate, apex acuminate, base attenuate, subcoriaceous; both surfaces scabrous with whip-shaped hairs and capitate glands; lateral veins 9–10-paired; petioles up to 2.5 cm long. Capitulescences terminal and axillary, paniculate. Capitula campanulate, 10–12 mm long, pedunculate. Receptacles flat, 2–2.5 mm in diam., hairy. Involucres narrowly campanulate, in 4–5 series, 5–6 mm long, 3–3.5 mm in diam., herbaceous. Phyllaries light green or purple apically, margin piliferous, outer surface arachnoid, capitate glands; the outer and the middle ones ovate, apex obtuse and apiculate; the inner ones ovate-lanceolate to oblong, apex obtuse or apiculate. Florets 8–12; corollas funnelform, purple or white, glandular; corolla tubes 4–5 mm long; corolla lobes 2.5–3 mm long. Anthers yellowish, 2.5–3 mm long, apical appendage acute, base acute. Styles purple, 5–7 mm long, branches 2–3 mm long. Achenes subterete, 2.5–3.5 mm long, 10-ribbed, pubescent, glands capitate. Pappus bristles, the inner ones 6–6.5 mm long.

#### Distribution.

Thailand: Mae Hong Son, Chiang Mai, Chiang Rai. China (Yunnan), India, Myanmar, Laos.

#### Specimens examined.

Thailand. Chiang Mai, Chiangdao wildlife sanctuary, 19°26.08'N, 98°53.76'E, 20 Dec 2003, *S. Bunwong* 78 (KKU, US); Doi Chiangdao, 30 Jan 1921, *A.F.G. Kerr* 4729 (BK, BM, K); Doi Chiangdao, 9 Mar 1982, *Y. Paisooksantivatana* 840-82 (BK); Doi Chiangdao, 25 Jan 1996, *W. Nanakorn et al.* 5683 (QBG); Chiang Rai, Doi Thung, 15 Jan 1975, *R. Geesink, P. Hiepko & C. Phengklai* 8277 (BKF, C, K, L, P).

#### Ecology.

Hill evergreen or pine-oak forest, alt. 1000–1800 m; flowering January to March.

#### Diagnostic characters.

*Acilepis kingii* is recognized by obtuse and apiculate phyllaries and loose paniculate capitulescence.

#### Vernacular name.

Muang Doi (ม่วงดอย).

### Acilepis namnaoensis

(H.Koyama) H.Rob. & Skvarla, Proc. Biol. Soc. Washington 122(2): 141. 2009.

urn:lsid:ipni.org:names:60456953-2:1.2

http://species-id.net/wiki/Acilepis_namnaoensis

Acilepis namnaoensis *Vernonia namnaoensis* H.Koyama, Bull. Natl. Sci. Mus. Tokyo, Ser. B 30(1): 22. 2004.

#### Type.

Thailand, Phetchabun, Nam Nao national park, *H. Koyama, H.Terao & Th. Wongprasert* T-311840 (holotype: KYO!). Fig. 5C.

#### Description.

Perennial herbs, 1–2 m tall. Stems erect, conspicuously ribbed, villose. Leaves 15–20 by 5–8 cm, obovate or oblanceolate, margin serrate, apex acuminate, base cuneate, subcoriaceous; both surfaces scabrous with whip-shaped hairs and capitate glands; lateral veins 6–8-paired; petioles up to 10 mm long. Capitulescences terminal and axillary, paniculate. Capitula campanulate, 12–15 mm long, pedunculate. Receptacle flat, 2–2.5 mm in diam., hairy. Involucres campanulate, in 4–5 series, 7–8 mm long, 6–7 mm in diam., herbaceous. Phyllaries light green, margin piliferous, outer surface densely arachnoid, glands capitate; the outer and the middle ones ovate, apex spinose; the inner ones ovate-lanceolate, apex apiculate. Florets ca. 20; corollas funnelform, purple, glandular; corolla tubes ca. 5 mm long; corolla lobes ca. 3 mm long. Anthers yellowish, ca. 3 mm long, apical appendage acute, base acute. Styles purple, 5–8 mm long, branches 1.5–2.5 mm long. Achenes subterete, 3.5–4 mm long, 10-ribbed, covered with sparse hairs and glands. Pappus bristles, the inner ones 6–7 mm long.

#### Distribution.

Thailand: Phetchabun, Chaiyaphum. Endemic.

#### Specimens examined.

Thailand. Chaiyaphum, Chulabhon Dam, 16°32.10'N, 101°38.93'E, 8 Oct 2007, *S. Bunwong* 385 (KKU, US); Chulabhon Dam, 27 Dec 1982, *H. Koyama, H.Terao & Th. Wongprasert* T-311840 (KYO).

#### Ecology.

Dry dipterocarp or dry evergreen forest, alt. 800 m; flowering November to December.

#### Diagnostic characters.

*Acilepis namnaoensis* is characterized by its cup-shaped involucre and spinose phyllaries with whitish tomentum. Its specific epithet is derived from Nam Nao national park in Pethchabun province.

#### Vernacular name.

Nad Muang Nam Nao (หนาดม่วงน้ำหนาว).

### Acilepis ngaoensis

(H.Koyama) H.Rob. & Skvarla, Proc. Biol. Soc. Washington 122(2): 141. 2009.

urn:lsid:ipni.org:names:60456956-2:1.2

http://species-id.net/wiki/Acilepis_ngaoensis

Vernonia ngaoensis H.Koyama, Bull. Natl. Sci. Mus. Tokyo, Ser. B 30(1): 25. 2004.

#### Type.

Thailand, Ranong, Muang District, Ngao waterfall, *T. Shimitzu, H. Toyokuni, H. Koyama, T. Yahara & C. Niyomdham* T-26543 (holotype: KYO!). Fig. 5D.

#### Description.

Perennial herbs, 60–120 cm tall. Stems erect, conspicuously ribbed, puberulous. Leaves 10–20 by 2–5 cm, elliptic or oblanceolate, margin serrate, apex acuminate to acuminate, base cuneate, subcoriaceous; both surfaces scabrous with whip-shaped hairs and capitate glands; lateral veins 9–11-paired; petioles up to 10 mm long. Capitulescences terminal or axillary, solitary or loosely paniculate. Capitula campanulate, 10–20 mm long, pedunculate. Receptacle flat, hairy. Involucres campanulate, in 6–7 series, 10–15 mm long, herbaceous. Phyllaries light green or purple apically, margin piliferous, outer surface arachnoid, glands capitate; the outer and the middle ones ovate or lanceolate, apex spinose; the inner ones lanceolate or oblong, apex apiculate. Florets ca. 80; corollas funnelform, purple, glandular; corolla tubes 7–8 mm long; corolla lobes 3–4 mm long. Anthers 3.5–4 mm long, apical appendage acute, base obtuse. Styles purple, 7–9 mm long, branches 3–3.5 mm long. Achenes subterete, 3–4 mm long, 10-ribbed, pubescent with twin hairs and glands. Pappus bristles, the inner ones 8–9 mm long.

#### Distribution.

Thailand: Ranong. Endemic.

#### Specimens examined.

Thailand. Ranong, Muang District, Ngao waterfall, 9°51.38'N, 98°37.68'E, 25 Jan 2008, *S. Bunwong* 386 (KKU, US); Ngao waterfall, 10 Dec 1974, *S. Indrapong* 84 (BKF); Ngao waterfall, 10 Dec 1974, *S. Indrapong* 843 (K, L); Ngao waterfall, 28 Jan 1968, *J.F. Maxwell* 87-88 (AAU, BKF, CMU, L, P, PSU); Ngao waterfall, 22 Nov 1993, *T. Santisuk* 650 (BKF); Ngao waterfall, 8 Dec 1979, *T. Shimitzu, H.Toyokuni, H. Koyama, T. Yahara & C. Niyomdham* T-26543 (KYO).

#### Ecology.

Rocky dry dipterocarp, alt. 100 m; flowering December to January.

#### Diagnostic characters.

*Acilepis ngaoensis* is recognized by having large capitula with long peduncles, phyllaries not reflexed, and capitulescences solitary or loosely paniculate. Its specific epithet is derived from Ngao waterfall national park in Ranong province.

#### Vernacular name.

Nad Muang Ngao (หนาดม่วงหงาว).

### Acilepis peguensis

(C.B.Clarke) H.Rob. & Skvarla, Proc. Biol. Soc. Washington 122(2): 141. 2009.

urn:lsid:ipni.org:names:60456958-2:1.2

http://species-id.net/wiki/Acilepis_peguensis

Vernonia peguensis C.B.Clarke, Compos. Ind.: 13. 1876.
Acilepis peguensis Type. Myanmar, Yomah province, Pegu, *S. Kurz* 882 (holotype: K!). Fig. 5E.
Vernonia kradungensis H. Koyama, Bull. Natl. Sci. Mus. Tokyo, Ser. B 30(2): 72. 2005.
Acilepis peguensis Type: Thailand, Loei, Phu Kradung national park, *H. Koyama, H. Terao & Th. Wongprasert* T-31211 (holotype: KYO!).

#### Description.

Perennial herbs, 0.5–150 cm tall. Stems erect, conspicuously ribbed, sericeous. Leaves 10–20 by 3–6 cm, oblanceolate or obovate, margin serrate, apex acute or acuminate, base attenuate, chartaceous; both surfaces scabrous with whip-shaped hairs and capitate glands; lateral veins 4–8-paired; petioles up to 30 mm long. Capitulescences terminal or axillary, paniculate. Capitula campanulate, 10–12 mm long, pedunculate. Receptacle flat, 2–2.5 mm in diam., hairy. Involucres narrowly campanulate or slightly oblong-cylindrical, in 4–5 series, 5–6 mm long, 3–4 mm in diam., herbaceous. Phyllaries light green, margin piliferous, outer surface arachnoid, glands capitate; the outer and the middle ones ovate, apex acuminate or aristate; the inner ones lanceolate to oblong, apex acuminate. Florets 10–15; corollas funnelform, white or purple, glandular; corolla tubes 4.5–5 mm long; corolla lobes 2.5–3 mm long. Anthers purple, 3–3.5 mm long, apical appendage acute, base obtuse. Styles purple, 6–7 mm long, branches 2–2.5 mm long. Achenes subterete, 2.5–3.5 mm long, 10-ribbed, glandular. Pappus bristles, the inner ones 5.5–6 mm long.

#### Distribution.

Thailand: Loei. Myanmar.

#### Specimens examined.

Thailand, Loei, Phu Kradung national park, 16°52.25'N, 101°50.74'E, 23 Dec 2007, *S. Bunwong* 372 (KKU, US); Phu Kradung national park, 7 Jul 1949, *Bunpheng* 264 (K); Phu Kradung national park,18 Dec 1982, *H. Koyama, H. Terao & Th. Wongprasert* T-31211 (KYO); Phu Kradung national park, 4 Nov 1984, *G. Murata, C. Phengklai, S. Mitsuta & T. Yahara* T-43083 (BKF, L, KYO); Phu Kradung national park, 4 Nov 1984, *H. Nagamasu & N. Nantasan* T-43024 (AAU, BKF, KYO, US).

#### Ecology.

Deciduous or hill dry evergreen forest, alt. 800 m; flowering November to December.

#### Diagnostic characters.

The distinctive features of *Acilepis peguensis* are membranaceous leaves and achenes without hair.

#### Vernacular name.

Dok Khon Kai Noi (ดอกขนไก่น้อย).

### Acilepis principis

(Gagnep.) H.Rob. & Skvarla, Proc. Biol. Soc. Washington 122(2): 143. 2009.

urn:lsid:ipni.org:names:77114239-1:1.2

http://species-id.net/wiki/Acilepis_principis

Vernonia principis Gagnep., Bull. Mus. Hist. Nat. 25: 490. 1919.

#### Type.

Thailand, Molu, *Prince H. d’Orleans* s.n. (holotype: P!). Fig. 5F.

#### Description.

Perennial herbs, 0.5–2 m tall. Stems erect, conspicuously ribbed, puberulous. Leaves 10–25 by 3–8 cm, ovate-lanceolate or lanceolate, margin serrate, apex acute, base cuneate, subcoriaceous; upper surface scabrous without glands; lower surface scabrous with whip-shaped hairs and capitate glands, lateral veins 9–15-paired; petioles up to 10 mm long. Capitulescences terminal or axillary, paniculate. Capitula campanulate, 10–15 mm long, pedunculate. Receptacle flat, 2.5–3 mm in diam., hairy. Involucres campanulate, in 5–6 series, 7–8 mm long, 4–5 mm in diam., herbaceous. Phyllaries light green or purple apically, margin piliferous, outer surface arachnoid, eglandular; the outer and the middle ones ovate, apex aristate or apiculate; the inner ones ovate-lanceolate, apex obtuse or apiculate. Florets 20–25; corollas funnelform, purple or white, glabrous; corolla tubes 5–6 mm long; corolla lobes 3–4 mm long. Anthers purple, 3–4 mm long, apical appendage acute, base obtuse. Styles purple, 8–9 mm long, branches 3–4 mm long. Achenes subterete, 2.5–3 mm long, 10-ribbed, pubescent with twin hairs. Pappus bristles, the inner ones 7–8 mm long.

#### Distribution.

Thailand: Chiang Rai, Nan, Lamphun, Lampang, Tak. Endemic.

#### Specimens examined.

Thailand. Chiang Rai, Khun Jae, 30 Dec 1977, *J.F. Maxwell* 97-1540 (L); Chiang Rai, Doi Langka, 27 Dec 1965, *K. Iwatsuki & N. Fukuoka* T3464 (AAU, BKF); Chiang Mai, Doi Nang Ka, 16 Nov 1980, *Put* 3449 (BM, K); Nan, Doi Phu Kha national park, 17 Jan 2002, *P. Srisanga* 2392 (QBG); Doi Phu Kha national park, 1 Mar 2002, *P. Srisanga, S. Sasirat, W. Pongamornkul, S. Sukiam & P. Panyachan* 2483 (QBG); Lamphun, Mae Tah, 19 Nov 1993, *J.F. Maxwell* 93-1382 (L); Lampang, Khun Tan national park, 18°29.74'N, 99°16.20'E, 15 Jan 2008, *S. Bunwong* 382 (KKU, US); Tak, Doi Hua Mod, 11 Dec 1933, *H.B.G Garrett* 855 (BKF, E, K, L, P); Doi Hua Mod, 24 Jan, 1964, *B. Hansen, G. Seidenfaden & T. Smitinand* 10862 (C, K, L, P).

#### Diagnostic characters.

This species is similar to *Acilepis kingii* and *Acilepis saligna* in capitula shape but differs in the achenes without hair and with the upper leaves lanceolate-oblong with caudate apex or falcate shape.

#### Ecology.

Evergreen forest, alt. 600–1400 m; flowering December to March.

#### Vernacular name.

Ma Nee Nin (มณีนิล).

### Acilepis pseudosutepensis

(H.Koyama) H.Rob. & Skvarla, Proc. Biol. Soc. Washington 122(2): 143. 2009.

urn:lsid:ipni.org:names:77114240-1:1.2

http://species-id.net/wiki/Acilepis_pseudosutepensis

Vernonia pseudosutepensis H.Koyama, Bull. Natl. Sci. Mus. Tokyo, Ser. B 31(2): 74. 2005.

#### Type.

Thailand, Uthai Thani, Huay Ka Kaeng; *J.F. Maxwell* 76-94 (holotype: AAU!, isotype: L!).

#### Description.

Perennial herbs, 60–150 cm tall. Stems erect, conspicuously ribbed, sericeous. Leaves simple, alternate at base, 10–14 by 4–7 cm, ovate-lanceolate or elliptic, margin serrate, apex acuminate, base cuneate or acuminate, subcoriaceous; upper surface scabrous without glands; lower surface scabrous with whip-shaped hairs and capitate glands; lateral veins 9–11-paired; petioles up to 10 mm long. Capitulescences terminal and axillary, paniculate. Capitula campanulate, 10–15 mm long, pedunculate. Receptacle flat, 1.5–3 mm in diam., glabrous. Involucres narrowly campanulate, in 5–6 series, 8–10 mm long, 3–4 mm in diam., herbaceous. Phyllaries light green or purple apically, margin piliferous, outer surface densely arachnoid, capitate glands; the outer and the middle ones ovate or lanceolate, apex apiculate or aristate; the inner ones ovate-lanceolate to oblong, apex apiculate. Florets 10–15; corollas funnelform, purple, glandular; corolla tubes 7–8 mm long; corolla lobes 3–4 mm long. Anthers 3.5–4 mm long, apical appendage acute, base obtuse. Styles purple, 7–9 mm long, branches 3–3.5 mm long. Achenes subterete, 2–3.5 mm long, 10-ribbed, pubescent with twin hairs and capitate glands. Pappus bristles, the inner ones 6–7 mm long.

#### Distribution.

Thailand: Tak, Uthai Thani, Kanchanaburi. Endemic.

#### Specimens examined.

Thailand, Tak, Doi Hua Mod, 15°57.63'N, 98°51.43'E, 16 Feb 2008, *S. Bunwong* 388 (KKU, US); Doi Hua Mod, 18 Dec 1961, *K. Larsen* 8794 (C, L), *K. Larsen* 8990 (C, L); Tak, Maesod, 26 Jan 1985, *Y. Paisooksantivatana* 1558-85 (BK); Uthai Thani, Huay Kha Khang, 10 Feb 1976, *J.F. Maxwell* 76-94 (AAU, BK, L); Kanchanaburi, Sangklaburi, Thaung Yai Naresuan, 14 Jan 1994, *J.F. Maxwell* 94-41 (L); Kanchanaburi, Sai Yok, *C. Phengklai* 346 (BKF, L).

#### Diagnostic characters.

*Acilepis pseudosutepensis* is recognized by elongate peduncles, loose capitulescences, densely villose and scarious phyllaries.

#### Ecology.

Limestone mountain or mixed evergreen forest, alt. 200–400 m; flowering December to February.

#### Vernacular name.

Ka Ma Plong (กะม่าปล่อง).

### Acilepis saligna

(DC.) H.Rob., Proc. Biol. Soc. Washington 112(1): 226. 1999.

urn:lsid:ipni.org:names:1010886-1:1.1.2.1.1.2

http://species-id.net/wiki/Acilepis_saligna

Conyza saligna Wall., Numer. List [Wallich] no. 3061, comp. no. 171, *nom. nud.*
Vernonia saligna DC., Prodr. 5: 33. 1836.

#### Type.

India Orient, Silhet, *Wallich* 3061 (isotype: E!, isotype: G!, holotype: K!). Fig. 5G.

#### Description.

Perennial herbs, 2–2.5 m tall. Stems erect, conspicuously ribbed, sericeous. Leaves 10–15 by 3–6 cm, lanceolate or elliptic, margin serrate, apex acuminate, base cuneate, subcoriaceous; both surfaces scabrous with whip-shaped hairs and capitate glands; lateral veins 6–10-paired; petioles up to 6 mm long. Capitulescences terminal or axillary, paniculate. Capitula campanulate, 6–7 mm long, pedunculate. Receptacle flat, 2–2.5 mm in diam., hairy. Involucres in 5–6 series, 6–7 mm long, 3.5–4.5 mm in diam., herbaceous, campanulate. Phyllaries light green or purple apically, margin piliferous, outer surface arachnoid, glands capitate; the outer and the middle ones ovate, apex acuminate or cuspidate; the inner ones ovate-lanceolate to oblong, apex rounded or apiculate. Florets 10–15; corollas funnelform, purple, puberulous glandular; corolla tubes 6–7 mm long; corolla lobes 2–3 mm long. Anthers purple, 2.5–3 mm long, apical appendage acute, base obtuse. Styles purple, 5–7 mm long, branches 1.5–2 mm long. Achenes subterete, 3–3.5 mm long, 10-ribbed, pubescent with twin hairs and capitate glands. Pappus bristles, the inner ones 6–7 mm long.

#### Distribution.

Thailand: Mae Hong Son, Chiang Mai. China (Yunnan), India, Nepal, Bhutan, Myanmar, Laos, Vietnam.

#### Specimens examined.

Thailand, Mae Hong Son, Pang Ma Pha, 19°26.77'N, 98°19.15'E, 8 Dec 2007, *S. Bunwong* 357 (KKU, US); Chiang Mai, Doi Chiangdao, 5 Dec 1965, *E. Hennipman* 3220 (BKF, P).

#### Diagnostic characters.

*Acilepis saligna* differs from *Acilepis kingii* by its sessile leaves and smaller capitula in a dense panicle.

#### Ecology.

Pine-oak forest, alt. 1100–1400 m; flowering November to December.

#### Vernacular name.

Pai Lin (ไพลิน).

### Acilepis silhetensis

(DC.) H.Rob., Proc. Biol. Soc. Washington 112(1): 227. 1999.

urn:lsid:ipni.org:names:1010888-1:1.1.2.1.1.2

http://species-id.net/wiki/Acilepis_silhetensis

Vernonia bracteata Wall., Numer. List [Wallich] no. 2921, comp. no. 31, *nom. nud.*
Decaneurum silhetense DC., Prodr. 5: 67. 1836.
Vernonia silhetensis (DC.) Hand.-Mazz., Symb. Sin. 7: 1084. 1936.

#### Type.

India, Silhet; *Wallich* 2921 (holotype: E!). Fig. 5H.

#### Description.

Perennial herbs, 1–3 m tall. Stems erect, inconspicuously ribbed, pilose. Leaves 7–12 by 2–4 cm, lanceolate or oblanceolate, margin serrate, apex acute or acuminate, base attenuate, subcoriaceous; both surfaces scabrous with whip-shaped hairs and capitate glands; lateral veins 5–10-paired; petioles up to 1 cm long. Capitulescences terminal, paniculate or solitary. Capitula campanulate, 15–20 mm long, pedunculate. Receptacle flat, 6–10 mm in diam., hairy. Involucres in 6–7 series, 11–18 mm long, 10–15 mm in diam., herbaceous, campanulate. Phyllaries dark purple or green with purple apex, margin piliferous, outer surface arachnoid lacking glands; the outer and the middle ones ovate, apex acuminate, upper half strongly reflexed; the inner ones ovate-lanceolate to oblong, apex acuminate or aristate. Florets 50–75; corollas funnelform, purple, glandular; corolla tubes 8–12 mm long; corolla lobes 3.5–5 mm long. Anthers 3.5–4.5 mm long, apical appendage acute, base obtuse. Styles purple, 10–12 mm long, branches 3.5–4.5 mm long. Achenes subterete, 4–5 mm long, 10-ribbed, glandular. Pappus bristles, the inner ones 6.5–8 mm long.

#### Distribution.

Thailand: Mae Hong Son, Chiang Mai, Lamphun, Lampang, Phetchabun, Loei, Khon Kaen. China, India, Bhutan, Myanmar, Laos, Myanmar.

#### Specimens examined.

Thailand, Chiang Mai, Queen Sirikit botanic garden, 18°53.89'N, 98°51.61'E, 10 Dec 2007, *S. Bunwong* 364 (KKU, US); Mae Hong Son, Pai, 20 Oct 1979, *T. Shimizu, H. Toyokuni, H. Koyama, T. Yahama & T. Santisuk* T-20113 (BKF, L); Chiang Mai, Doi Chiangdao, Nov 25 1963, *Adisai* 714 (BK); Doi Chiangdao, 24 Jul 1998, *K. Buchuai* 279 (AAU, BKF); Doi Chiangdao, 16 Dec 1983, *N. Fukuoka & M. Ito* T-35208 (BKF); Doi Chiangdao,3 Dec 1984, *H. Koyama* T-39781 (BKF, L); Doi Chiangdao, 5 Aug 1988, *H. Koyama* T- 61104 (BKF); Doi Chiangdao,29 Nov 1984, *H. Koyama, T. Yahara, H. Nagamasu, W. Nanakorn & N. Nantasan* T-39710 (AAU, BKF, L); Doi Chiangdao, 30 Nov 1984, *H. Koyama, T. Yahara, H. Nagamasu, W. Nanakorn & N. Nantasan* T- 39736 (BKF, L); Doi Chiangdao, 4 Nov 1995, *J.F. Maxwell* 95-1065 (CMU, BKF); Doi Chiangdao, 13 Oct 1931, *Put* 311 (BK, BM, K); Chiang Mai, Doi Sutep, 6 Oct 1982, *Konta Th. Wongprasert & B. Sangkhachand* 29741 (BKF); Doi Sutep,28 Nov 1984, *H. Koyama, S. Mitsuta, T. Yahara & H. Nagamasu* T-39671 (BKF, L); Doi Inthanon, 6 Dec 1984, *S. Mitsuta, T. Yamada & H. Nagamazu* T-46454 (BKF); Doi Inthanon, 1 Oct 1971, *G. Murata, K. Iwatsuki, C. Phengklai & C. Charoenpol* T-15497 (BKF); Doi Inthanon, 1 Oct 1971, *G. Murata, K. Iwatsuki, C. Phengklai & C. Charoenpol* T-15498 (BKF, P); Mae Rim, 21 Dec, 1985, *Y. Paisooksantivatana* 1645b-85 (BK); Mae Rim, 26 Dec 1987, *R. Pooma* 43 (BKF); Lampang, Jae son national park, 2 Dec 1995, *J.F. Maxwell* 95-1227 (BKF, L); Doi Khun Tan, 2 Jan 1985, *H. Koyama & C. Phengklai* T-39197 (BKF, L); Phetchabun, Namnao national park, 18 Nov 1973, *J.F. Maxwell* 73-636 (AAU, BK, BKF); Loei, Phu Kradung, 24 Dec 1991, *Dee* 195 (E); Phu Kradung, 21 Oct 1989, *Din* 125 (BKF); Phu Kradung, 19 Dec 1982, *H. Koyama, H. Terao & Th. Wongprasert* T-31330 (C, BKF); Phu Kradung, 5 Sep 1988, *R. Pooma* 62 (BKF); Phu Kradung, 21 Oct 1967, *Prayad* 1076 (BK); Khon Kaen, route to Nam Nao national park, 18 Nov 1979, *T. Shimizu, H. Toyokuni, H. Koyama, T. Yahama & T. Santisuk* T-22531 (BKF, L).

#### Diagnostic characters.

This species is distinguished by its strongly reflexed phyllaries and glandular achenes lacking trichomes.

#### Ecology.

Dipterocarp or pine-oak forest, alt. 700–1350 m; flowering August to January.

#### Vernacular names.

Phak Phet Kao Kum (ผักเผ็ดข้าวกํ่า), Ya Klung (หญ้าคลัง), Ya Hang Nok Kiew (หญ้าหางนกเขียว), Hudsakuen (หัสคึน).

### Acilepis squarrosa

D.Don, Prodr. Fl. Nepal.: 169. 1825.

urn:lsid:ipni.org:names:174526-1:1.3

http://species-id.net/wiki/Acilepis_squarrosa

Vernonia teres Wall., Numer. List [Wallich] no. 2926, comp. no. 36, *nom. nud.*
Vernonia squarrosa (D.Don) Less., Linnaea 6: 678. 1831.

#### Type.

Nepal, *Hamilton* s.n. (not seen). Fig. 5I.

#### Description.

Perennial herbs, 30–80 cm tall. Stems erect, inconspicuously ribbed, villose. Leaves 3–10 by 1–3 cm, oblanceolate, margin serrate, apex acute, base cuneate, coriaceous; both surfaces scabrous with whip-shaped hairs and capitate glands; lateral veins 5–10-paired; petioles up to 5 mm long. Capitulescences terminal and axillary, solitary. Capitula campanulate, 15–20 mm long, sessile or subsessile. Receptacle flat, 4.5–5.5 mm in diam., glabrous. Involucres campanulate, in 12–13 series, 15–20 mm long, 10–15 mm in diam., herbaceous. Phyllaries light green or purple apically, margin piliferous, outer surface arachnoid and lacking glands; the outer and the middle ones ovate or lanceolate, apex acuminate or apiculate; the inner ones ovate-lanceolate to oblong, apex acuminate or apiculate. Florets 50–80; corollas funnelform, purple, puberulous, glands capitate; corolla tubes 7–10 mm long; corolla lobes 4–6 mm long. Anthers 3–3.5 mm long, apical appendage acute, base obtuse. Styles purple, 9–11 mm long, branches 2–3 mm long. Achenes subterete, 2.8–3.2 mm long, 10-ribbed, pubescent without glands. Pappus bristles, the inner ones 7–11 mm long.

#### Distribution.

Thailand: Mae Hong Son, Chiang Mai, Chiang Rai, Lamphun, Lampang, Tak, Phetchabun, Loei, Sakon Nakhon, Mukdahan, Kalasin, Khon Kaen, Chaiyaphum, Nakhon Ratchasima, Surin, Uthai Thani, Kanchanaburi, Lop Buri, Prachin Buri, Chon Buri. China (Yunnan), India, Bhutan, Myanmar, Laos.

#### Specimens examined.

Thailand, Phetchabun, Nam Nao national park, 16°44.29'N, 101°34.19'E, 26 Dec 2007, *S. Bunwong* 374 (KKU, US); Mae Hong Son, Pai, 16 Jan 1983, *H. Koyama & H. Terao & Th. Wongprasert* T-32609 (BKF); Pai, 16 Jan 1786, *Y. Paisooksantivatana* 1780-86 (BK); Pai, 23 Nov 1989, *Y. Paisooksantivatana* 2548-89 (BK); Chiang Mai, Hod, 13 Jan 1983, *H. Koyama & H. Terao & Th. Wongprasert* T-32454 (BKF); Hod, 13 Nov 1965, *Prayad* 44 (BK); Hod, 4 Dec 1975, *J. Sadakorn* 664 (BK); Lamphun, Maeli, 3 Nov 1925, *Winit* 1515 (BKF, K); Lampang, Doi Khun Tan, 28 Dec 1984, *H. Koyama & C. Phengklai* T-39155 (BKF, L); Tak, Maesod, 13 Jan 1989, *Y. Paisooksantivatana* 2306-89 (BK); Phetchabun, Nam Nao national park, 18 Nov 1973, *J.F. Maxwell* 73-630 (AAU, BK); Nam Nao national park, 23 Jun 1975, *J.F. Maxwell* 75-626 (AAU, BK, L); Loei, Paa See Than, 2 Sep 1991, *Dee* 321 (BKF); Sakon Nakhon, 23 Nov 1962, *Adisai* 155 (BK); Sakon Nakhon, Phu Phan, 15 Nov 1984, *G. Murata & C. Phengklai* 50372 (BKF); Mukdahan, route number 212, 12 Dec 1982, *H. Koyama & H. Terao & Th. Wongprasert* T-32161 (BKF); Kalasin, Phu Sing, Sahadsakhan, 22 Oct 1975, *S. Sutheesorn* 3503 (BK); Khon Kaen, route to Nam Nao national park, 18 Nov 1979, *T. Shimitzu, H. Toyokuni, H. Koyama, T. Yahara & C. Niyomdham* T-22533 (BKF, L); Chaiyaphum, Kang Kraw, 23 Oct 1965, *S. Sutheesorn* 3048 (BK); Nakhon Ratchasima, Pak Thong Chai, 10 Nov 1963, *Pradit* 523 (BK); Hui Taleng, 21 Dec 1928, *Put* 2175 (BK, BM, K); Surin, 3 Dec 1976, *C. Phengklai et al.* 3595 (BKF); Uthai Thani, 4 Dec 1977, *C. Phengklai et al.* 3993 (BKF); Kanchanaburi, Thong Pha Phume, 25 Jan 1983, *H. Koyama & H. Terao & Th. Wongprasert* T-32909 (BKF); Thong Pha Phume, *T. Shimitzu, H. Toyokuni, H. Koyama, T. Yahara & C. Niyomdham* T-21892 (BKF, L).

#### Diagnostic characters.

*Acilepis squarrosa* is easily recognized by its large capitula (with more than 100 phyllaries) sessile in the axils of the leaves

#### Ecology.

Dipterocarp, deciduous, hill evergreen or pine-oak forest, alt. 10–1200 m; flowering September to May.

#### Vernacular names.

Kiang Pa Chang (เกี๋ยงพาช้าง), Nat Dhum (หนาดดำ), Nat Khum (หนาดคำ).

### Acilepis sutepensis

(Kerr) H.Rob. & Skvarla, Proc. Biol. Soc. Washington 122(2): 144. 2009.

urn:lsid:ipni.org:names:77114242-1:1.2

http://species-id.net/wiki/Acilepis_sutepensis

Vernonia sutepensis Kerr, Bull. Misc. Inform., Kew. 1935: 329. 1935.

#### Type.

Thailand, Chiang Mai, Doi Sutep, *A.F.G. Kerr* 3561 (holotype: K!).

#### Description.

Perennial herbs, 60–150 cm tall. Stems erect, conspicuously ribbed, villose. Leaves rosulate, 10–14 by 4–7 cm, ovate-lanceolate or elliptic, margin serrate, apex acuminate, base cuneate or acuminate, subcoriaceous; both surfaces scabrous with whip-shaped hairs and capitate glands, lateral veins 9–11-paired; petioles up to 10 mm long. Capitulescences terminal and axillary, paniculate. Capitula campanulate, 10–15 mm long, pedunculate. Receptacle flat, 1.5–3 mm in diam., glabrous. Involucres narrowly campanulate, in 5–6 series, 8–10 mm long, 3–4 mm in diam., herbaceous. Phyllaries light green or purple apically, margin piliferous, outer surface sparsely arachnoid with capitate glands; the outer and the middle ones ovate or lanceolate, apex apiculate or aristate; the inner ones ovate-lanceolate to oblong, apex apiculate. Florets 13–20; corollas funnelform, purple, glandular; corolla tubes 7–8 mm long; corolla lobes 3–4 mm long. Anthers 3.5–4 mm long, apical appendage acute, base obtuse. Styles purple, 7–9 mm long, branches 3–3.5 mm long. Achenes subterete, 2–3.5 mm long, 10-ribbed, pubescent with twin hairs and capitate glands. Pappus bristles, the inner ones 8–9 mm long.

#### Distribution.

Thailand: Chiang Mai, Chiang Rai, Lamphun, Lampang, Nan. Endemic.

#### Specimens examined.

Thailand, Chiang Mai, Doi Sutep Pui national park, 18°48.39'N, 98°54.90'E, 9 Dec 2007, *S. Bunwong* 361 (KKU, US); Doi Sutep Pui national park, 14 Feb 1988, *J.F.Maxwell* 88-182 (AAU, CMU, BKF, L); Doi Sutep Pui national park, 22 Feb 1987, *C. Niyomdham &R. Kubat* 1347 (AAU, BKF, C, E, K, L); Doi Sutep Pui national park, 24 Mar 1965, *C.H. & B. Sangkachand* 287 (BKF); Doi Sutep Pui national park, 20 Mar 1951, *T. Smitinand & P. Suvarnakoset* 152 (BKF); Doi Sutep Pui national park, 13 Feb 1958, *Th. Sørensen, K. Larsen & B. Hansen* 6903 (BKF, C, K); Doi Chiangdao, 2 Mar 1974, *T. Koyama, C. Phengklai, C. Niyomdham, H. Okada*& *P.J.O’Connor* 15599 (AAU, BKF); Doi Chiangdao, 1 Mar 1995, *J.F.Maxwell* 95-168 (BKF, CMU, L); Doi Chiangdao, 19 Feb 1997, *J.F.Maxwell* 97-157 (CMU, L); Chiang Rai, Wieng Pa Pao, 30 Dec 1993, *J.F.Maxwell* 97-1540; Nan, Doi Phu Kha national park, 28 Feb 2002, *P. Srisanga, S. Sasirat, W. Pongamornkul, S. Sukiam & P. Panyachan* 2477 (QBG).

#### Diagnostic characters.

*Acilepis sutepensis* is distinguished from *Acilepis pseudosutepensis* by having larger capitula and sparse hairs on the phyllaries.

#### Ecology.

Hill evergreen or pine-oak forest, 1100–1500 m; flowering November to March.

#### Vernacular name.

Mu Nin (มุนิน).

### Acilepis tonkinensis

(Gagnep.) H.Rob. & Skvarla, Proc. Biol. Soc. Washington 122(2): 144. 2009.

urn:lsid:ipni.org:names:77114244-1:1.2

http://species-id.net/wiki/Acilepis_tonkinensis

Vernonia tonkinensis Gagnep., Bull. Mus. Hist. Nat. 25: 492. 1919.

#### Type.

Vietnam, Tonkin, *Balansa* 3078 (holotype: P!).

#### Description.

Perennial herbs, 1–1.5 m tall. Stem erect, inconspicuously ribbed, villose. Leaves alternate, 5–10 by 1.5–4 cm, elliptic or obovate, margin serrate, apex acute, base cuneate or truncate, subcoriaceous; upper surface scabrous, lower surface pilose glandular; lateral veins 6–11-paired; petioles up to 5 mm long. Capitulescences terminal, paniculate. Capitula campanulate, 9–12 mm long, pedunculate. Receptacle flat, hairy. Involucres broadly campanulate, in 5–6 series, 8–10 mm long, herbaceous. Phyllaries purple, outer surface arachnoid, glands capitate; the outer and the middle ones ovate, apex acute; the inner ones ovate-lanceolate to oblong, apex apiculate. Florets ca. 20; corollas funnelform, purple, glandular; corolla tubes ca. 5 mm long; corolla lobes ca. 4 mm long. Achenes subterete, ca. 3 mm long, 10-ribbed, pubescent with twin hairs and capitate glands. Pappus bristles, the inner ones 8–9 mm long.

#### Distribution.

Thailand: Chiang Mai. Vietnam.

#### Specimens examined.

Thailand, Chiang Mai, Doi Sa Ket, 18°52.26'N, 99°8.17'E, 15 Feb 1983, *H. Koyama, H. Terao & Th. Wongprasert* T-33577 (KYO); Doi Sa Ket, *J. Kubiniok* 392/6 (CMU).

#### Diagnostic characters.

*Acilepis tonkinensis* can be recognized by its pubescent receptacle and broadly ovate phyllaries with acute apices.

#### Ecology.

Granite bedrock in dry dipterocarp forest, alt. 930–1200 m; flowering January to February.

#### Vernacular name.

Dok Muang Doi (ดอกม่วงดอย).

### Acilepis virgata

(Gagnep.) H.Rob. & Skvarla, Proc. Biol. Soc. Washington 122(2): 144. 2009.

urn:lsid:ipni.org:names:77114245-1:1.2

http://species-id.net/wiki/Acilepis_virgata

Vernonia virgata Gagnep., Bull. Mus. Hist. Nat. 25: 493. 1919.

#### Type.

Laos, Xieng-Kouang, *Spire* 1302 (holotype: P!).

#### Description.

Perennial herbs, ca. 1 m tall. Stems erect, conspicuously ribbed, pilose. Leaves cauline 8–10 by 2.5–3.5 cm, elliptic, margin serrate, apex acuminate, base cuneate, subcoriaceous; upper surface pilose along main and lateral veins; lower surface pilose glandular; lateral veins 7–10-paired; petioles up to 5 mm long. Capitulescences terminal, paniculate. Capitula campanulate, 7–10 mm long, pedunculate. Receptacle flat, hairy. Involucres campanulate, herbaceous, in 5–6 series, ca. 6 mm long. Phyllaries green with purple tips, outer surface arachnoid, glands capitate; the outer and the middle ones ovate-lanceolate, apex acuminate; the inner ones ovate-lanceolate to oblong, apex acute. Florets ca. 20; corollas funnelform, purple, glandular; corolla tubes ca. 3 mm long; corolla lobes ca. 4 mm long. Achenes subterete, ca. 2 mm long, 10-ribbed, pubescent with twin hairs and capitate glands. Pappus bristles, the inner ones 6–7 mm long.

#### Distribution.

Thailand: Surat Thani. Laos.

#### Specimens examined.

Thailand, Surat Thani, Khao Sok national park, 8°54.99'N, 98°31.68'E, 2 Mar 1983, *H. Koyama, H. Terao & Th. Wongprasert* T-33960 (KYO).

#### Diagnostic characters.

*Acilepis virgata* is distinguished from *Acilepis tonkinensis* by phyllaries that are nearly scarious and capitula that are long pedunculate in loosely paniculate capitulescences.

#### Ecology.

Evergreen forest, alt. 180 m; flowering March.

#### Vernacular name.

Muang Ngam (ม่วงงาม).

### Camchaya

Gagnep., Notul. Syst. 4: 14. 1920.

urn:lsid:ipni.org:names:8072-1:1.1.2.1.1.1

http://species-id.net/wiki/Camchaya

#### Type.

*Camchaya kampotensis* Gagnep., Notul. Syst. 4: 14. 1920.

#### Description.

Annual herbs. Stems erect, pubescent. Leaves simple, alternate, usually petiolate; lamina ovate to lanceolate, pubescent with hairs and glands, margin serrate, apex acute to acuminate, base attenuate, usually chartaceous. Capitulescences terminal or axillary, paniculate or corymbose. Capitula campanulate or hemispherical, pedunculate, homogamous; florets bisexual and fertile. Involucres campanulate or hemispherical. Phyllaries imbricate, persistent, the outer and the middle ones ovate or lanceolate, the inner ones linear-oblong, sometimes pubescent, glands capitate. Corollas purple or white, narrowly funnelform, pubescent with hairs and/or capitate glands; corolla lobes 5. Anthers apical appendage acute, base rounded. Styles purple, 2-branched, slender, acute, inner surface covered with stigmatic papillae, outer surface and shaft covered with sweeping hairs reaching to below style bifurcation. Achenes obovate, usually 10-ribbed, carpopodium absent. Pappus in one series of often deciduous bristles. Pollen echinolophate, 6-porate, without micropuncta.

Seven species are recognized in Thailand.

#### Key to the species

**Table:** 

1	Phyllaries broadly ovate without marginal spines	2
–	Phyllaries broadly ovate with marginal spines	3
2	Achenes 4–5-ribbed	*Camchaya gracilis*
–	Achenes 10-ribbed	*Camchaya thailandica*
3	Achenes 5 (6–9)-ribbed	*Camchaya pentagona*
–	Achenes 10-ribbed	4
4	Phyllaries without glands, spines up to 10 mm	*Camchaya spinulifera*
–	Phyllaries with glands, spines up to 5 mm	5
5	Phyllaries acuminate; achenes 2.5–3 mm long	*Camchaya kampotensis*
–	Phyllaries aristate or apiculate; achenes 1.5–2 mm	6
6	Leaves with T-shaped hairs; phyllaries spinose ≤ 1 mm long	*Camchaya loloana*
–	Leaves without T-shaped hairs; phyllaries spinose ≥ 1 mm long	*Camchaya tenuiflora*

### Camchaya gracilis

(Gagnep.) S. Bunwong & H. Rob., Proc. Biol. Soc. Washington 122(3): 361. 2009.

urn:lsid:ipni.org:names:77114296-1:1.3

http://species-id.net/wiki/Camchaya_gracilis

Iodocephalus gracilis Thorel ex Gagnep., Notul. Syst. (Paris) 4: 17. 1920.

#### Type.

**Figure 6.**
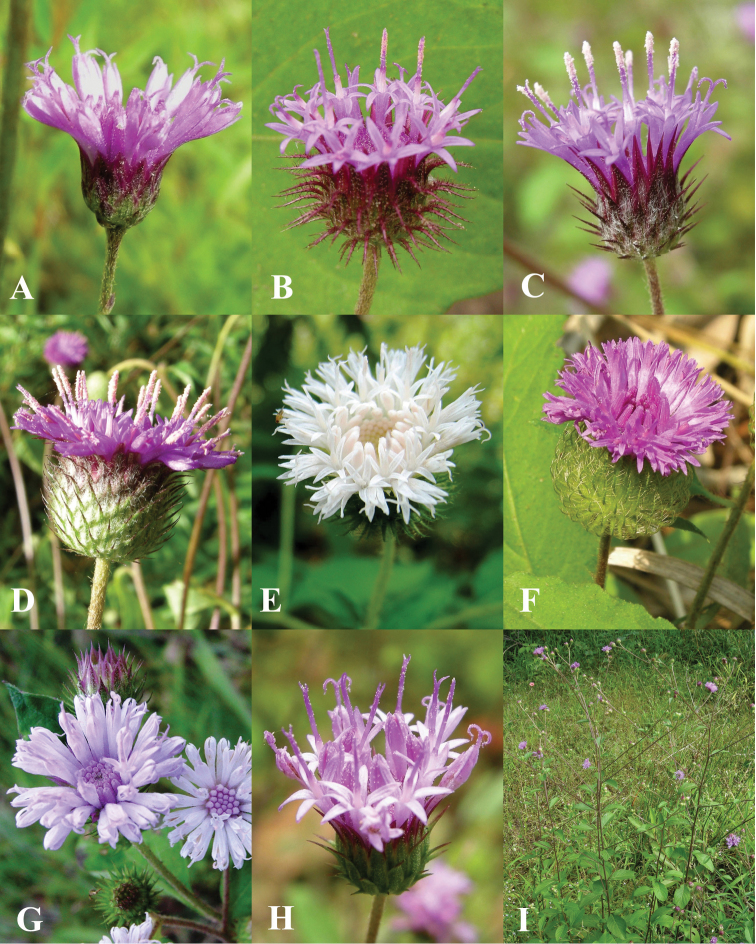
Morphology of *Vernonieae* in Thailand 2. **A** *Camchaya gracilis* **B** *Camchaya loloana* **C** *Camchaya loloana* var. *mukdahanensis* **D** *Camchaya pentagona* **E–F** *Camchaya spinulifera* **G** *Camchaya tenuiflora* **H–I** *Camchaya thailandica*.

Laos, Bassac, *Thorel* 2396 (holotype: P!). Fig. 6A.

#### Description.

Annual herbs, 50–100 cm tall. Stems erect, terete, inconspicuously ribbed; villose with uniseriate hairs, T-shaped hairs and glands. Leaves alternate, elliptic-oblong, 3–6 by 0.3–1 cm, margin serrate or entire, apex acute, base attenuate, subcoriaceous; upper surface scabrous lacking glands, lower surface scabrous with whip-shaped hairs and capitate glands, lateral veins 8–10-paired; petioles up to 3 mm long. Capitulescences terminal and axillary, corymbose. Capitula campanulate, 8–10 mm long, pedunculate. Receptacle flat, 2–3 mm in diam., glabrous. Involucres campanulate, in 4–5 series, 7–8 mm long, 6–7 mm in diam. Phyllaries imbricate, light green or purple, margin entire without spines, outer surface arachnoid, glandular; the outer ones ovate, apex acute to acuminate; the inner ones ovate-lanceolate, apex acute to acuminate. Florets 50–70; corollas funnelform, purple, puberulous, glands capitate; corolla tubes 3–5 mm long; corolla lobes 2–3 mm long. Anthers ca. 2 mm long, apical appendage acute, base rounded. Styles purple, 5–7 mm long, branches 1.5–2 mm long. Achenes obovate, ca. 2 mm long, puberulous with twin hairs and capitate glands, 4–5-ribbed, carpopodium absent. Pappus 1–2 mm long in 1 series, frequently deciduous or lacking.

#### Distribution.

Thailand: Ubon Ratchathani. Laos.

#### Specimens examined.

Thailand, Ubon Ratchathani, route number 2222, 15°18.59'N, 105°23.15'E, route number 2222, 28 Oct 2007, *S. Bunwong* 346 (KKU, US). Route number 2112 to Khong Chiam, 16 Sep 2004, *R. Pooma, K. Phattanahirankanok & S. Sirimongkol* 4737 (AAU).

#### Diagnostic characters.

*Camchaya gracilis* is characterized by phyllaries without marginal spines, achenes with 4–5 ribs and leaves that are narrowly elliptic-oblong. This species is included in *Camchaya* as it shares 6-porate pollen found in no other genera.

#### Ecology.

Dipterocarp forest, alt. 150 m; flowering October to December.

#### Vernacular name.

Ao Ra Nid (อรนิช).

### Camchaya kampotensis

Gagnep., Notul. Syst. 4: 14. 1920.

urn:lsid:ipni.org:names:188171-1:1.1.2.1.1.1

http://species-id.net/wiki/Camchaya_kampotensis

#### Type.

Myanmar, Kampot, *Geoffray* 331 (holotype: P!).

#### Description.

Annual herbs 30–80 cm tall. Stems erect, terete, inconspicuously ribbed, pubescent with T-shaped hairs and glands. Leaves lanceolate, 7–25 by 5–9 cm, margin serrate, apex acute, base attenuate, chartaceous; both surfaces puberulous glandular; petioles up to 7 mm long. Capitulescences terminal and axillary, paniculate or solitary. Capitula campanulate, 10–15 mm long, pedunculate. Involucres campanulate, herbaceous, in 6–7 series, 9–12 mm long. Phyllaries imbricate, green or light purple, margin with spines up to 5 mm long, outer surface puberulous, glands capitate; the outer and the inner ones lanceolate, apex acuminate; the inner ones lanceolate or linear-oblong, apex acute. Corollas funnelform, purple, puberulous, glands capitate. Anthers ca. 2 mm long, apical appendage acute, base rounded. Styles purple. Achenes obovate, 2.5–3 mm long, glabrous, 10-ribbed. Pappus in 1 series of deciduous bristles or lacking.

#### Distribution.

Thailand: Ubon Ratchathani, Saraburi, Trat, Chanthaburi. Myanmar.

#### Specimens examined.

Thailand. Trat, Kao Kuap, 12°20.0'N, 102°25.0'E, 26 Nov 1929, *A.F.G. Kerr* 17783 (BK, E, K, P); Ubon Ratchathani, Khong Chiam, *T. Santisuk* 526 (BKF); Saraburi, Phiang, 30 Aug 1995, *J.F. Maxwell* 99-624 (CMU). Myanmar. Poporkvil, 4 Dec 1964, *T. Kira, K. Hozumi, K. Yoda & S. Kokawa* 102 (BKF); Bokor, Kampot, 20 Dec 1965, *J.E.Vidal* 4766 (L); Kampot, *M. Martin* 769 (L).

#### Diagnostic characters.

*Camchaya kampotensis* can be recognized by its acute to broadly acuminate phyllaries and its large achenes.

#### Ecology.

Evergreen forest, alt. 700–900 m; flowering December.

#### Vernacular name.

Nin La Pad (นิลปัทน์).

### Camchaya loloana

Kerr, Bull. Misc. Inform., Kew. 1935: 327. 1935.

urn:lsid:ipni.org:names:188172-1:1.1.1.3

http://species-id.net/wiki/Camchaya_loloana

#### Types.

Thailand, Chiang Mai, Chiangdao district; *A.F.G. Kerr* 6650 (holotype: BK!, isotype: BM!, isotype: K!, isotype: P!). Fig. 6B.

### Camchaya loloana var. loloana

#### Description.

Annual herbs, 30–70 cm tall. Stems erect, terete, inconspicuously ribbed, pubescent with T-shaped hairs and glands. Leaves ovate, 3–10 by 2–4 cm, margin serrate, apex acute or acuminate, base attenuate, chartaceous; both surfaces scabrous with whip-shaped hairs, cylindrical hairs and capitate glands; lateral veins 8–12-paired; petioles up to 10 mm long. Capitulescences terminal and axillary, paniculate. Capitula campanulate, 9–10 mm long, pedunculate. Receptacle convex, 3.5–4 mm in diam., glabrous. Involucres campanulate, in 7–8 series, 8–10 mm long. Phyllaries imbricate, greenish with purple apex, margin with spines up to 1 mm long, outer surface arachnoid, glands capitate; the outer lanceolate, apex spinose; the inner ones lanceolate or linear-oblong, apex acuminate. Florets 65–100; corollas funnelform, purple rarely white, puberulous, glands capitate; corolla tubes 7–9.5 mm long; corolla lobes 2.5–3 mm long. Anthers 1.8–2.5 mm long, apical appendage acute, base rounded. Styles purple, 7–11 mm long, branches ca. 2 mm long. Achenes obovate, 1.3–1.7 mm long, glabrous, 10-ribbed. Pappus in one series, bristles 1.5–3 mm long, absent or deciduous.

#### Distribution.

Thailand: Chiang Mai, Lampang, Phitsanulok, Khon Kaen, Nakhon Ratchasima, Ubon Ratchathani, Kanchanaburi, Saraburi. China (Yunnan), Laos, Myanmar.

#### Specimens examined.

Thailand, Khon Kaen, Phu Wiang national park, 16°40.93'N, 102°14.15'E, 1 Oct 2007, *S. Bunwong* 330 (KKU, US); Chiang Mai, Doi Chiangdao, 9 Nov 1922, *A.F.G. Kerr* 6650 (BM, K, P); Doi Chiangdao, 30 Jul 1968, *K. Larsen, T. Santisuk & E. Warncke* 2862 (BKF, E); Doi Chiangdao, 27 Sep 1994, *W. Nanakorn et al.* 1821 (QBG); Doi Chang, 29 Oct 1979, *T. Shimizu, H. Toyokumi, H. Koyama, T. Yahara & T. Santisuk* 20639 (AAU, BKF, L); Khon Kaen, 18 Sep 1994, *W. Nanakorn et al.* 1626 (QBG),9 Jan 1997, *W. Nanakorn et al.* 8472 (QBG); Ubon Ratchathani, Sirinthon Dam, 27 Oct 2007, *S. Bunwong* 339 (KKU, US); Kanchanaburi, Kritee, 9 Jul 1973, *R. Geesink & C. Phengklai* 6192 (AAU, BKF, E, L, P); Sangkhlaburi, 14 Jul 1973, *S. Sutheesorn* 2637 (BK); Saraburi, Chaibadan, 15 Dec 1923, *A.F.G. Kerr* 7982 (BK, BM, K, P).

#### Diagnostic characters.

*Camchaya loloana* is recognized by having short spines on the margins of the phyllaries, 10-ribbed achenes, and leaves with T-shaped hairs.

#### Ecology.

On limestone in dipterocarp, dry evergreen, and hill evergreen forest, alt. 400–1500 m; flowering July to December.

#### Vernacular name.

Dok Lea (ดอกแล่), Phu Muang (พู่ม่วง).

### Camchaya loloana var. mukdahanensis

H.Koyama, Acta Phytotax. Geobot. 35(1-3): 52. 1984.

urn:lsid:ipni.org:names:918305-1:1.5

#### Type.

Thailand, Mukdahan, Muang District, Dongman Village, *H. Koyama et al.* T-30941 (holotype: KYO!). Fig. 6C.

#### Description.

Annual herbs, 10–70 cm tall. Stems erect, terete, inconspicuously ribbed, scabrous with uniseriate hairs and T-shaped hairs. Leaves alternate, 5–11 by 2–5 cm, ovate or lanceolate, margin serrate or undulate, apex acute or acuminate, base attenuate, chartaceous; both surfaces scabrous with whip-shaped hairs, cylindrical hairs and capitate glands; lateral veins 5–9-paired; petioles up to 15 mm long. Capitulescences terminal and axillary, paniculate. Capitula campanulate, 9–10 mm long, pedunculate. Receptacle convex, 2–2.5 mm in diam., glabrous. Involucres campanulate, in 5–6 series, 7–9 mm long, 4–6 mm in diam. Phyllaries greenish with purple apex, margin with spines up to 0.2 mm long, outer surface arachnoid, glands capitate; the outer lanceolate, apex spinose; the inner ones lanceolate to oblong, apex acuminate. Florets 30–70; corollas funnelform, purple, puberulous, glands capitate; corolla tubes ca. 5.5 mm long; corolla lobes 1.5–2 mm long. Anthers 1.5–2 mm long, apical appendage acute, base rounded. Styles purple, 5–6 mm long, branches ca. 2 mm long. Achenes obovate, 1.3–5 mm long, glabrous, 10-ribbed. Pappus in one series of bristles 1.5–2 mm, absent or deciduous.

#### Distribution.

Thailand: Nong Khai, Mukdahan, Ubon Ratchathani. Laos.

#### Specimens examined.

Thailand, Mukdahan, Phu Pha Thoep national park, 16°26.08'N, 104°48.33'E, 22 Oct 2007, *S. Bunwong* 338 (KKU, US); Phu Pha Thoep national park, 12 Dec 1982, *H. Koyama, H. Terao & Th. Wongprasert* T-30866 (BKF); Phu Pha Thoep national park, 13 Dec 1982, *H. Koyama et al.* T-30941 (KYO); Mukdahan, Cham Cha Iee, 13 Dec 1982, *H. Koyama, H. Terao & Th. Wongprasert* T-30904 (BKF, L); Nong Khai, route number 2186, 16 Dec 1982, *H. Koyama, H. Terao & Th. Wongprasert* T-31137 (BKF, L); Ubon Ratchathani, Sirinthon Dam, 9 Dec 1982, *H. Koyama, H. Terao & Th. Wongprasert* T-30662 (BKF, L); Sirinthon Dam, *M. Norsangsri* 1158 (QBG); Gang Ta na national park, 27 Oct 2007, *S. Bunwong* 343 (KKU, US),

#### Diagnostic characters.

*Camchaya loloana* var. *mukdahanensis* differs from the typical variety by having smaller capitula, a shorter involucre, and fewer florets.

#### Ecology.

Rocky area in dipterocarp forest, alt. 250–400 m; flowering August to January.

#### Vernacular name.

Phu Muang (พู่ม่วง).

### Camchaya pentagona

H.Koyama, Acta Phytotax. Geobot. 35(1-3): 53. 1984.

urn:lsid:ipni.org:names:903797-1:1.5

http://species-id.net/wiki/Camchaya_pentagona

#### Type.

Thailand, Ubon Ratchathani; *H. Koyama, H. Terao & Th. Wongprasert* T-30791 (holotype: KYO!, isotype: AAU!, isotype: L!). Fig. 6D.

#### Description.

Annual herbs, 20–60 cm tall. Stems erect, terete, inconspicuously ribbed, scabrous with uniseriate and T-shaped hairs. Leaves alternate, ovate or lanceolate, 3–10 by 2–4 cm, margin serrate or sinuate, apex acute, base attenuate, chartaceous; both surfaces scabrous with whip-shaped hairs, cylindrical hairs and capitate glands; lateral veins 5–10-paired; petioles up to 2 cm long. Capitulescences terminal and axillary, paniculate. Capitula broadly campanulate or hemispherical, 13–15 mm long, peduculate. Receptacle convex, 3.5–6 mm in diam., glabrous. Involucres hemispherical, in 8–9 series, 11–12 mm long. Phyllaries imbricate, greenish with purple apex, margin with spines up to 0.5 mm long, outer surface arachnoid and lacking glands; the outer and the middle ones lanceolate, apex spinose; the inner ones lanceolate to oblong, apex acuminate. Florets 80–150; corollas funnelform, purple, puberulous, glands capitate; corolla tubes 6–7 mm long; corolla lobes 2–2.5 mm long. Anthers ca. 2 mm long, apical appendage acute, base rounded. Styles purple, 6–7.5 mm long, branches ca. 2 mm long. Achenes obovate, 1.7–2 mm long, glabrous, 5(–6–9)-ribbed. Pappus in one series of bristles, 2–3.5 mm long, present in some florets, deciduous.

#### Distribution.

Thailand: Ubon Ratchathani. Endemic.

#### Specimens examined.

Thailand, Ubon Ratchathani, Sai Moon subdistrict, route number 2222, 15°16.92'N, 105°18.56'E, 27 Oct 2007, *S. Bunwong* 344 (KKU, US); Nachaluay Distr. 10 Dec 1982, *H. Koyama, H.Terao & Th. Wongprasert* T-30760 (L); Muang Sam Sib District, 11 Dec 1982, *H. Koyama, H.Terao & Th. Wongprasert* 30791 (KYO, AAU, L).

#### Diagnostic characters.

*Camchaya pentagona* is distinguished by its typically 5-ribbed achenes.

#### Ecology.

Disturbed area in dipterocarp forest, alt. 220–300 m; flowering October to December.

#### Vernacular name.

Phu Tab Tim (พู่ทับทิม).

### Camchaya spinulifera

H.Koyama, Acta Phytotax. Geobot. 35(1-3): 54. 1984.

urn:lsid:ipni.org:names:903798-1:1.5

http://species-id.net/wiki/Camchaya_spinulifera

#### Type.

Thailand, Mukdahan, Nikomkhamsoi district, Phu Moo forest park; *H. Koyama, H.Terao & Th. Wongprasert* T-30837 (holotype: KYO!, isotype: L!). Figs 6E–F.

#### Description.

Annual herbs, 40–100 cm tall. Stems erect, terete, inconspicuously ribbed, scabrous with uniseriate and T-shaped hairs. Leaves alternate, ovate to lanceolate, 4–10 by 1.5–4 cm, margin serrate, apex acute, base attenuate, chartaceous; both surfaces scabrous with whip-shaped hairs, cylindrical hairs, T-shaped hairs and capitate glands; lateral veins 5–13-paired; petioles up to 1 cm long. Capitulescences terminal and axillary, paniculate. Capitula campanulate or hemispherical, 10–15 mm long, pedunculate. Receptacle convex, 4.5–6.5 mm in diam., glabrous. Involucres hemispherical, in 8–9 series, 10–15 mm long, 10–20 mm in diam. Phyllaries imbricate, greenish with purple apex, margin with spines up to 10 mm long, outer surface arachnoid without glands; the outer lanceolate, apex spinose; the inner ones lanceolate to oblong, apex acuminate. Florets 130–220; corollas funnelform, purple, puberulous, glands capitate; corolla tubes 7.5–9 mm long; corolla lobes 2.5–3 mm long. Anthers ca. 2 mm long, apical appendage acute, base rounded. Styles purple, 8–11 mm long, branches 2–2.5 mm long. Achenes obovate, 1.3–1.5 mm long, glabrous, 10-ribbed. Pappus in one series of bristles, 1.5–3 mm long, present in some florets, deciduous.

#### Distribution.

Thailand: Nong Khai, Sakon Nakhon, Udon Thani, Mukdahan, Kalasin, Chaiyaphum, Ubon Ratchathani. Endemic.

#### Specimens examined.

Thailand, Sakon Nakhon, Phu Phan national park, 17°4.0'N, 103°58.0'E, 1 Oct 2007, *S. Bunwong* 332 (KKU, US); Phu Phan national park, 14 Dec 1982, *Koyama, H.Terao & Th. Wongprasert* T-31007 (L); Phu Phan national park, 14 Dec 1982, *Koyama, H.Terao & Th. Wongprasert* T-31054 (BKF); Phu Phan national park, 12 Nov 1984, *G. Murata, C. Phengklai, S. Mitsuta, T. Yahara, H. Nagamasu & N. Nantasan* T-50638 (BKF); Phu Phan national park, 12 Nov 1984, *G. Murata, C. Phengklai, S. Mitsuta, T. Yahara, H. Nagamasu & N. Nantasan* T-51352 (BKF); Phu Phan national park, 25 Nov 1962, *P. Suvanakoses* 1947 (BKF); Nong Khai, Phu Woa, 21 Oct 2007, *S. Bunwong* 336 (KKU, US); route number 212, 15 Dec 1982, *Koyama, H.Terao & Th. Wongprasert* T-31068 (BKF); Bung Kla, 8 Nov 1996, *C. Niyomdham* 4897 (BKF); Bung Kla, 17 Nov 1966, *T. Smitinand* 10097 (BKF, L); Udon Thani, 29 Sep 2007, *S. Bunwong* 327 (KKU, US); Mukdahan, Nikomkamsoi, Phu Moo, 11 Dec 1982, *Koyama, H.Terao & Th. Wongprasert* T-30837 (AAU, BKF); route number 2030,13 Dec 1982, *Koyama, H.Terao & Th. Wongprasert* T-30954 (BKF); Kalasin, Sahatsakhan, 20 Oct 1975, *S. Sutheesorn* 3500 (BK); Chaiyaphum, Pha Hin Ngam, 21 Nov 1992, *S. Suddee* 6 (BKF).

#### Diagnostic characters.

*Camchaya spinulifera* is recognized by having the longest marginal spines on their phyllaries of any species, and the lack of glands on the phyllaries.

#### Ecology.

Rocky area in dipterocarp forest, alt. 200–300 m; flowering September to December.

#### Vernacular name.

Phu Muang (พู่ม่วง), Up-Pa-Kud (อุปคุต).

### Camchaya tenuiflora

Kerr, Bull. Misc. Inform., Kew. 1935: 327. 1935.

urn:lsid:ipni.org:names:188174-1:1.1.1.2

http://species-id.net/wiki/Camchaya_tenuiflora

#### Type.

Thailand, Bangkok, *A.F.G. Kerr* 20563 (holotype: BK!, isotype: E!, isotype: K!, isotype: L!, isotype: P!). Fig. 6G.

#### Description.

Annual herbs, 20–70 cm tall. Stems erect, terete, inconspicuously ribbed, scabrous with uniseriate and T-shaped hairs. Leaves alternate, ovate or lanceolate, 3–10 by 1.5–2.5 cm, margin serrate, apex acute, base attenuate, chartaceous; both surfaces scabrous with whip-shaped hairs, cylindrical hairs and capitate glands; lateral veins 5–10-paired; petioles up to 10 mm long. Capitulescences terminal and axillary, paniculate and solitary. Capitula campanulate, 8–10 mm long, pedunculate. Receptacle convex, 1.5–3 mm in diam., glabrous. Involucres campanulate, in 6–7 series, 8–9 mm long, 6–10 mm in diam. Phyllaries imbricate, light green with purple apex, margin with spines up to 5 mm long, outer surface arachnoid glandular; the outer and the middle ones lanceolate, apex spinose; the inner ones lanceolate to oblong apex acuminate. Florets 40–60; corollas funnelform, purple or white, puberulous, glands capitate; corolla tubes 4–6 mm long; corolla lobes 1.5–2.5 mm long. Anthers ca. 2 mm long, apical appendage acute, base rounded. Styles purple, 4–7 mm long, branches 1.5–1.7 mm long. Achenes obovate, 1.5–1.7 mm long, glabrous, 10-ribbed. Pappus in 1 series of bristles, 1–4 mm long, present in some florets, deciduous.

#### Distribution.

Thailand: Chiang Mai, Chiang Rai, Loei, Chaiyaphum, Nakhon Ratchasima. Endemic.

#### Specimens examined.

Thailand, Loei, Nahaew, Phu Suan Sai national park, 17°30.21'N, 100°56.35'E, 6 Nov 2007, *S. Bunwong* 348 (KKU, US); Phu Suan Sai national park, *R. Pooma* 1231 (BKF); Chiang Rai, Wieng Pa Pao, 26 Oct 1997, *J.F.Maxwell* 197-1215 (BKF); Nakhon Ratchasima, Bua Yai, 1 Nov 1931, *Put* 4264 (BK, E, K, P).

#### Diagnostic characters.

*Camchaya tenuiflora* differs from *Camchaya loloana* by its longer marginal spine on phyllaries and leaf surfaces without T-shaped hair.

#### Ecology.

Open area in evergreen forest, alt. 700 m; flowering October to December.

#### Vernacular name.

Phu Ra Wee (พู่ระวี).

### Camchaya thailandica

Bunwong, Chantar. & S.C.Keeley, PhytoKeys 12: 53–57. 2012.

urn:lsid:ipni.org:names:77119225-1:1.5

http://species-id.net/wiki/Camchaya_thailandica

#### Type.

Thailand. Prov. Udon Thani, rare on rocky area in Phu Phrabat historical park, *S. Bunwong* 328 (holotype KKU!, isotype US!). Figs 6H–I.

#### Description.

Annual herbs, 50–100 cm tall. Stems erect, terete, inconspicuously ribbed; scabrous with uniseriate hairs, T-shaped hairs and glands. Leaves alternate, elliptic to oblong, 3–8 by 2–3 cm, margin serrate, apex acute, base attenuate, chartaceous; both surfaces puberulous with cylindrical hairs, T-shaped hairs and capitate glands; lateral veins 5–10-paired; petioles up to 2 cm long. Capitulescences terminal and axillary, corymbose. Capitula campanulate, 8–10 mm long, pedunculate. Receptacle convex, 2.5–3 mm in diam., glabrous. Involucres broadly campanulate, in 5–6 series, 7–8 mm long, 5–6 mm in diam. Phyllaries imbricate, light green with purple apex, margin pale without spine, outer surface arachnoid glandular; the outer and the middle ones ovate, apex acuminate; the inner ones lanceolate to oblong, apex acuminate. Florets 50–70; corollas funnelform, purple, puberulous, glands capitate; corolla tubes 6–7 mm long; corolla lobes 2.5–3 mm long. Anthers ca. 2 mm long, apical appendage acute, base rounded. Styles purple, 6–7 mm long, branches 2–2.5 mm long. Achenes obovate, ca. 1.5 mm long, glandular, 10-ribbed. Pappus in 1 series of bristles, 1–2 mm long, present in some florets, deciduous.

#### Distribution.

Thailand: Udon Thani. Endemic.

#### Specimens examined.

Thailand. Udon Thani, Ban Phue district, Phu Phrabat historical park, 17°43.84'N, 102°29.65'E, *S. Bunwong* 328 (KKU, US).

#### Diagnostic characters.

This species is similar to *Vernonia gracilis* in having ovate phyllaries which its without marginal spine but differs in 10-ribbed achenes and broadly ovate leaf shape.

#### Ecology.

Rocky areas in dipterocarp forest, alt. 300 m; flowering November to December.

#### Vernacular name.

Muk Udon (มุกอุดร).

### Cyanthillium

Blume, Bidjr. Fl. Ned. Ind. 15: 889. 1826.

urn:lsid:ipni.org:names:8489-1:1.1.2.2.1.2

http://species-id.net/wiki/Cyanthillium

#### Type.

*Cyanthillium villosum* Blume. Bijdr. Fl. Ned. Ind. 15: 889. 1826.

#### Description.

Annual herbs. Stems erect, pubescent with T-shaped hairs. Leaves simple, alternate, petiolate; lamina ovate, lanceolate, elliptic or rhombic, pubescent, margin serrate or undulate, apex acute or acuminate, base attenuate, chartaceous. Capitulescences terminal or axillary. Capitula discoid, homogamous, pedunculate, florets bisexual and fertile. Involucre imbricate, persistent. Corollas funnelform, purple to white, actinomorphic, corolla lobes 5. Anthers 5, syngenesious. Styles purple, 2-branched, inner surface covered with stigmatic papillae, outer surface covered with sweeping hairs reaching below style bifurcation. Achenes clavate or turbinate, 5–10-ribbed, carpopodium present. Pappus in one or two series, persistent, the outer ones are shorter than the inner ones. Pollen echinolophate, 3-porate, with micropuncta.

Three species are recognized in Thailand.

#### Key to the species

**Table:** 

1	Pappus in 2-series; capitula narrowly campanulate with 25–30 florets	2
–	Pappus in 1-series; capitula subglobose with 80–120 florets	*Cyanthillium patulum*
2	Herbs up to 1 m tall, stems and involucres sericeous; achene indistinctly ribbed	*Cyanthillium cinereum*
–	Herbs up to 2 m tall, stems and involucres pilose-villose to tomentose; achene with distinct 5–8 ribs	*Cyanthillium montanum*

### Cyanthillium cinereum

(L.) H.Rob., Proc. Biol. Soc. Washington 103(1): 252. 1990.

urn:lsid:ipni.org:names:1015961-2:1.3

http://species-id.net/wiki/Cyanthillium_cinereum

Conyza cinerea L., Sp. Pl.: 862. 1753.
Vernonia cinerea (L.) Less., Linnaea 4: 291. 1829.

#### Type.

**Figure 7.**
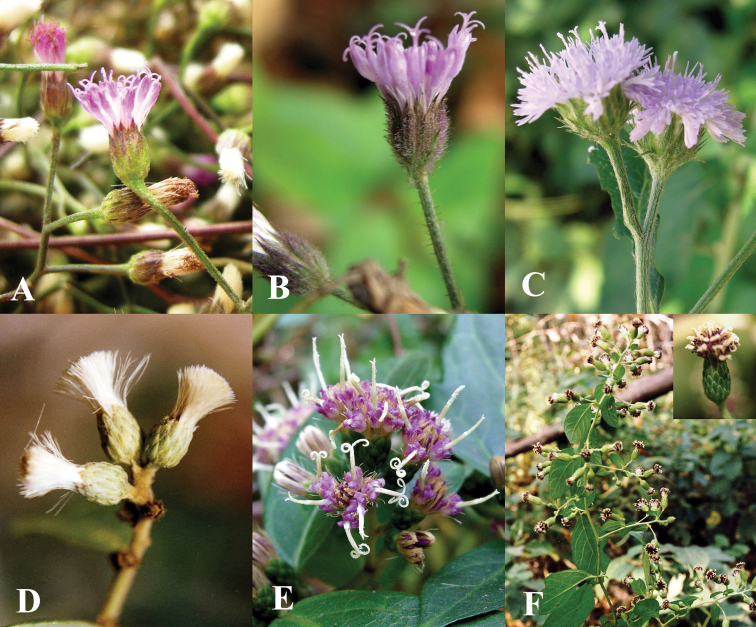
Morphology of *Vernonieae* in Thailand 3. **A** *Cyanthillium cinereum* **B** *Cyanthillium montanum* **C** *Cyanthillium patulum* **D** *Decaneuropsis cumingiana* **E** *Decaneuropsis eberhardtii* **F** *Decaneuropsis garrettiana*.

Sri Lanka, Herb. Hermann 3: 16, No. 419 (BM, lectotype designated by Jeffrey 1998: 224). Fig. 7A.

#### Description.

Annual herbs, 20–100 cm tall. Stems erect, conspicuously ribbed, sericeous. Leaves 3–5 by 2–3 cm, lanceolate or ovate to broadly ovate, margin undulate to serrate, apex acute to acuminate, base attenuate, chartaceous; upper surface sericeous without glands; lower surface sericeous with cylindrical hairs, T-shaped hairs and capitate glands, lateral veins 5–7-paired; petioles up to 2 cm long. Capitulescences terminal or axillary, paniculate. Capitula campanulate, 5–6 mm long, pedunculate. Receptacle flat, 2–2.5 mm in diam., glabrous. Involucres campanulate, in 3–4 series, 4–4.5 mm long, 2.5–3 mm in diam. Phyllaries imbricate, green with purple apex, margin piliferous, outer surface sericeous glandular; the outer and the middle ones lanceolate, apex acute to acuminate; the inner ones lanceolate to oblong, apex acuminate. Florets 25–30; corollas funnelform, purple or white, puberulous glandular; corolla tubes 3–3.5 mm long; corolla lobes ca. 1 mm long. Anthers ca. 0.6 mm long, apical appendage acute, base obtuse. Styles purple, ca. 3 mm long, branches ca. 0.5 mm long.Achenes clavate, 1.5–1.8 mm long, ribs inconspicuous, densely pubescent with twin hairs and capitate glands. Pappus in 2 series of bristles, the inner ones 3–3.5 mm long, persistent.

#### Distribution.

Thailand: Mae Hong Son, Chiang Mai, Chiang Rai, Phitsanulok, Nakhon Sawan, Phetchabun, Loei, Nong Bua Lum Phu, Udon Thani, Nong Khai, Sakon Nakhon, Nakhon Phanom, Mukdahan, Kalasin, Maha Sarakham, Khon Kaen, Chaiyaphum, Nakhon Ratchama, Ubon Ratchathani, Kanchanaburi, Lop Buri, Saraburi, Nakhon Nayok, Bangkok, Chumphon, Ranong, Phangnga, Phuket. Tropics and subtropics.

#### Specimens examined.

Thailand, Khon Kaen, Khon Kaen University, 16°28.03'N, 102°49.71'E, *S. Bunwong* 22 (KKU); Khon Kaen University, 2 Nov 1973, *T. Boonkird* 66 (BK); Chiang Rai, 20 Jan 1981, *Y. Paisooksantivatana* 495-81(BK); Chiang Rai, 22 Jan 1981, *Y. Paisooksantivatana* 521-81(BK); Chiang Rai, 27 Jan 1981, *Y. Paisooksantivatana* 548-81(BK), Chiang Mai, Doi Pui, 10 Mar 1982, *Y. Paisooksantivatana* 843-82 (BK); Nan, Muang District, 29 Nov 1986, *Y. Paisooksantivatana* 1887-86 (BK); Loei, Phu Kra Dung, 12 Jan 1960, *L.B. & E.C. Abbe & T. Smitinand* 2470 (BKF); Udon Thani, 23 Mar 1988, *Parikarn* 11 (BK); Nakhon Phanom, 13 May 1932, *A.F.G. Kerr* 21420 (BK, BM, E); Chaiyaphum, 1 Sep 1988, *Parikarn & Prayad* 95 (BK); Kanchanaburi, Si Sa Wat, 19 May 1962, *Kasem* 156 (BK); Saraburi, 23 Dec 1973, *J.F. Maxwell* 73-789 (AAU, BK); Bangkok, 28 Oct 1992, *W. Somprasong* 112 (BK); Ranong, 3 Dec 1928, *A.F.G. Kerr* 16459 (BK, BM, K),

#### Diagnostic characters.

The species is a widespread weed of disturbed areas throughout the tropics. It is widely known by its former name, *Vernonia cinerea*. Its leaf shape and capitula size are vary continuously so that plants in dry areas frequently have small capitula and leaves while those in more mesic situations have larger heads and leaves.

#### Ecology.

Open areas of dipterocarp or dry evergreen forest, alt. 0–250 m; flowering January to December.

#### Vernacular name.

Mor Noi (หมอน้อย), Kan Toop (ก้านธูป), Tua Haa Din (ถั่วแฮะดิน), Fa Rang Kok (ฝรั่งโคก), Suea Sam Kha (เสือสามขา), Ya Dok Kao (หญ้าดอกขาว), Ya La Ong (หญ้าละออง).

### Cyanthillium montanum

(C.B.Clarke) Bunwong, Chantar. & S.C.Keeley comb. & stat. nov.

urn:lsid:ipni.org:names:77138521-1

http://species-id.net/wiki/Cyanthillium_montanum

Vernonia cinerea b. *montana* C.B.Clarke, Comp. Ind.: 21. 1876.
Vernonia cinerea var. *montana* (C.B.Clarke) Kosterm., Blumea 1: 416. 1935.

#### Type.

India, Assam, Khasi hill (not seen). Fig. 7B.

#### Description.

Annual herbs, 1–2 m tall. Stems erect, conspicuously ribbed, pilose-villose. Leaves 7–8 by 2–4 cm, ovate or ovate-lanceolate, margin serrate or dentate, apex acute, base attenuate, chartaceous; upper surface puberulous glandular; lower surface villose with whip-shaped hairs, T-shaped hairs and capitate glands; lateral veins 5–7-paired; petioles up to 2 cm long. Capitulescences terminal or axillary, paniculate. Capitula narrowly campanulate, 5–7 mm long, pedunculate. Receptacle flat, 2–2.5 mm in diam., glabrous. Involucres narrowly campanulate, in 3–4 series, 5–6 mm long, 3–4 mm in diam. Phyllaries imbricate, purplish, margin piliferous, outer surface hirsute without glands; the outer and the middle ones lanceolate, acuminate; the inner ones lanceolate to oblong, apex acuminate. Florets 20–30; corollas funnelform, purple, corolla tubes 4–5 mm long; corolla lobes ca. 2 mm long. Anthers 0.5–2 mm long, apical appendage acute, base obtuse. Styles purple, 5–6 mm long, branches 1–2 mm long. Achenes clavate, 1.5–2 mm long, 5–8-ribbed, densely pubescent with twin hairs and capitate glands. Pappus in 2 series of bristles, the inner ones 5–6 mm long.

#### Distribution.

Thailand: Chiang Mai, Nan, Loei, Khon Kaen, Nakhon Ratchasima, Ubon Ratchathani. Laos, Myanmar, Vietnam, Indonesia (Sumatra).

#### Specimens examined.

Thailand, Loei, Phu Ruea national park, 17°28.29'N, 101°21.10'E, *S. Bunwong* 16, (KKU), *S. Bunwong* 62 (KKU); Chiang Mai, Mae Rim district, Pong Yang or rock town, 18°56.15'N, 98°49.36'E, 10 Dec 2007, *S. Bunwong* 371 (KKU, US); Doi Sutep, 22 Feb 1988, *J.F. Maxwell* 88-213 (CMU); Doi Sutep, 1 Aug 1958, *Th. Sørensen, K. Larsen*& *B. Hansen* 6588 (BKF, C); Doi Inthanon,8 Jan 1983, *H. Koyama, H. Terao & Th. Wongprasert* T-32130 (BKF); Chiangdao, 9 Feb 1983, *H. Koyama, H. Terao & Th. Wongprasert* T-33279 (BKF); Chiangdao, 3 Jan 1990, *J.F. Maxwell* 90-10 (CMU, E, L); Doi Maeya, 19 Jan 1983, *H. Koyama, H. Terao & Th. Wongprasert* T-32800 (BKF); Hod, 11 Jan 1983, *H. Koyama*, *H. Terao*& *Th. Wongprasert* T-32307 (BKF); Doi Anga, 16 Jan 1935, *H.B.G. Garrett* 922 (AAU, BKF E, L, P); Mae Thang, Doi Chang, 24 Oct 1979, *T. Shimizu, H. Toyokuni, H. Koyama, T. Yahama & T. Santisuk* T-20676 (BKF); Loei, Phu Kra Dung national park, 1 Sep 1988, *H. Takahashi & M.N. Tamura* T- 63331 (BKF); Phu Rue national park, 23 Dec 1982, *H. Koyama, H. Terao & Th. Wongprasert* T-31565 (BKF); Kanchanaburi, Bo Ploi, 8 Nov 1979, *T. Shimizu, H. Toyokuni, H. Koyama, T. Yahama & C. Niyomdham* T-22031 (BKF).

#### Diagnostic characters.

*Cyanthillium montanum* is separated from *Cyanthillium cinereum* by having villose to tomentose hairs on stems, branches, lower leaf surfaces and involucres, rather than appressed sericeous hairs. This species restricted to pine oak forest on the mountains.

#### Ecology.

Hill evergreen or pine-oak forest, alt. 500–1000 m; flowering October to March.

#### Vernacular name.

Pliw Doi (ปลิวดอย).

### Cyanthillium patulum

(Aiton) H.Rob. Proc. Biol. Soc. Washington 103(1): 252. 1990.

urn:lsid:ipni.org:names:962414-1:1.1.2.1.1.2

http://species-id.net/wiki/Cyanthillium_patulum

Conyza patula Aiton., Hortus Kew. 3: 184. 1789.
Vernonia patula (Aiton) Merrill, Philipp. J. Sci., C, 3: 439. 1909.

#### Type.

China, Cult. 1758, Philip Miller s.n. (not seen). Fig. 7C.

#### Description.

Annual herbs, 1–2 m tall. Stems erect, conspicuously ribbed, white sericeous. Leaves 3–10 by 2–5 cm, elliptic to ovate or slightly rhombic, margin serrate or slightly sinuate, apex acute or obtuse, base attenuate, chartaceous; upper surface puberulous glandular;lower surface sericeous with T-shaped hairs and capitate glands; lateral veins 4–8-paired; petioles up to 2 cm long. Capitulescences terminal or axillary, paniculate. Capitula broadly campanulate or subglobose, 7–10 mm long, pedunculate. Receptacle flat, 2–3 mm in diam., glabrous. Involucres hemispherical, in 4–5 series, 6–7 mm long, 5–6 mm in diam. Phyllaries imbricate, light green, margin piliferous, outer surface arachnoid glandular; the outer and the middle ones lanceolate, apex acuminate or aristate; the inner ones lanceolate to oblong, apex acuminate. Florets 80–120; corollas funnelform, purple or white, corolla tubes 4–5 mm long; corolla lobes ca. 2 mm long. Anthers 1.5–2 mm long, apical appendage acute, base obtuse. Styles purple, 4–5 mm long, branches 1–1.5 mm long. Achenes turbinate, 1–1.5 mm long, 5-ribbed, glandular. Pappus in 1 series of bristles, 2–3 mm long, deciduous.

#### Distribution.

Thailand: Nan, Sukhothai, Mukdahan, Buri Ram, Ubon Ratchathani, Bangkok, Samut Prakan, Prachin Buri, Surat Thani. China, India, Malay Peninsula, Laos, Myanmar, Philippines, New Guinea.

#### Specimens examined.

Thailand, Ubon Ratchathani, Chong Mek border crossing, 15°8.02'N, 105°28.01'E, 27 Oct 2007, *S. Bunwong* 341 (KKU, US); Nan, Chieng Klang, 28 Nov 1986, *Y. Paisooksantivatana* 1879-86 (BK); Sukhothai, 2 Nov, 1971, *J.F. Maxwell* 71-643 (BK, L); Mukdahan, Ban Wan, 6 Jun 1932, *M.C. Lakshnakara* 948 (BK, BM, K); Buri Ram, 24 Nov 1976, *C. Phengklai et al.* 3392 (BKF); Samut Prakan, 1 Feb 1970, *J.F. Maxwell* 70-19 (BK, L); Nakon Sawan, 1 Jul 1920, *Vanpruk* 1015 (BKF, K); Prachin Buri, Chakan, 30 Jan 1983, *H. Koyama, H. Terao & Th. Wongprasert* T-33138 (AAU, BKF, L); Surat Thani, Kiriras to Nikoon, 21 Oct 1970, *S. Sutheesorn* 1875 (BK).

#### Diagnostic characters.

*Cyanthillium patulum* differs from *Cyanthillium cinereum* by having only single series of pappus, a 5-ribbed achenes without hair and a globose capitula.

#### Ecology.

Open area in dry evergreen forest or secondary forest, alt. 0–100 m; flowering August to February.

#### Vernacular name.

Mud Muang (หมุดม่วง).

### Decaneuropsis

H.Rob. & Skvarla, Proc. Biol. Soc. Washington 120(3): 360. 2007.

urn:lsid:ipni.org:names:77094678-1:1.1

http://species-id.net/wiki/Decaneuropsis

#### Type.

*Vernonia cumingiana* Benth. in Hook.f., Kew Journ. 4: 232. 1852.

#### Description.

Perennial plants. Stems scandent, young branches terete, pubescent or glabrous. Leaves simple, alternate, petiolate, pubescent with uniseriate hairs; lamina ovate, lanceolate or elliptic; margin entire, apex acute or acuminate, base cuneate, subcoriaceous. Capitulescences terminal or axillary. Capitula discoid, homogamous, pedunculate, florets bisexual and fertile. Involucre campanulate, in 4–6-series. Phyllaries imbricate, 7–12 mm long, persistent, lacking glands. Corollas funnelform, purple or white, actinomorphic, corolla basal tubes slender, closely investing style shaft; corolla lobes 5. Anthers 5, syngenesious. Styles 2-branched, inner surface covered with stigmatic papillae, outer surface covered with sweeping hairs on the outer surface reaching below style bifurcation. Achenes clavate or turbinate, 10-ribbed, hairy, without glands, carpopodium present. Pappus in 2 series of bristles. Pollen subechinolophate, 3-colporate, with micropuncta.

Three species are recognized in Thailand.

#### Key to the species

**Table:** 

1	Phyllaries whitish puberulous	2
–	Phyllaries ferruginous tomentose	*Decaneuropsis cumingiana*
2	Phyllaries obtuse; leaves glandular	*Decaneuropsis garrettiana*
–	Phyllaries acute; leaves eglandular	*Decaneuropsis eberhardtii*

### Decaneuropsis cumingiana

(Benth.) H.Rob. & Skvarla, Proc. Biol. Soc. Washington 120(3): 364. 2007.

urn:lsid:ipni.org:names:77094684-1:1.1

http://species-id.net/wiki/Decaneuropsis_cumingiana

Vernonia cumingiana Benth. in Hook.f., Kew J. 4: 232. 1852.
Decaneuropsis cumingiana Type. Philippines, *M. Cuming* 1092 (holotype: G!). Fig. 7D.
Vernonia sangka Kerr, Bull. Misc. Inform. Kew 1935: 329. 1935.
Decaneuropsis cumingiana Type: Thailand, Kanchanaburu, Sangka, *A.F.G. Kerr* 8302 (holotype: K!).

#### Description.

Climbing or scandent shrubs, 3–10 m tall. Stems sprawling, young branches rounded, inconspicuously ribbed, ferruginous tomentose. Leaves 7–10 by 3–4 cm, elliptic to oblong-elliptic, margin entire, apex acute, base cuneate, subcoriaceous; upper surfaces puberulous with glands, lower surface sericeous with whip-shaped hairs, cylindrical hairs and capitate glands; lateral veins 5–6-paired; petioles up to 1 cm long. Capitulescences terminal and axillary, paniculate. Capitula campanulate, 12–15 mm long, pedunculate. Receptacle flat, 4–4.5 mm in diam., hairy. Involucres campanulate, in 5–6 series, 7–8 mm long, 6–7 mm in diam. Phyllaries imbricate, dull green, margin piliferous, outer surface ferruginous tomentose, glands capitate; the outer and the middle ones ovate, apex acute; the inner ones lanceolate to oblong, apex acute. Florets 20–30; corollas funnelform, purple, glandular, corolla tubes 5–6.5 mm long; corolla lobes ca. 2 mm long. Anthers 2.8–3 mm long, apical appendage acute, base obtuse. Styles purple, 7–7.5 mm long, branches 3.5–4 mm long. Achenes clavate, 3–3.5 mm long, 10-ribbed, puberulous without glands. Pappus in 2 series of bristles, the outer ones are shorter than the inner ones, the inner ones 9–9.5 mm long, persistent.

#### Distribution.

Thailand: Chiang Mai, Nan, Lampang, Phetchabun, Kanchanaburi, Phra Nakhon Si Ayutthaya, Saraburi, Nakhon Nayok, Yala. Hong Kong, India, Laos, Myanmar, Vietnam, Philippines, New Guinea.

#### Specimens examined.

Thailand, Phetchabun, Nam Nao national park, 16°44.29'N, 101°34.19'E, 18 Mar 2003, *S. Bunwong* 74 (KKU); Chiang Mai, Doi Sutep,15 Apr 1910, *A.F.G. Kerr* 1114 (BK, BM, K); Doi Sutep, 16 Feb 1958, *Th. Sørensen, K. Larsen & B. Hansen* 6942 (C, K); Lampang, 6 Mar 2525, *Winit* 1287 (BK, BKF, K), 31 Mar 1930, *Winit* 1916 (K); Saraburi, Mauk Lek, 9 Nov 1924, *A. Marcan* 1844 (BK, BM, K); Yala, Banang Sata, 10 Dec 1966, *B. Sangkhachand* 1409 (BKF).

#### Diagnostic characters.

*Decaneuropsis cumingiana* is recognized by its ferruginous pubescence on the leaves and phyllaries.

#### Ecology.

Evergreen or pine-oak forest, alt. 200–850 m; flowering November to April.

#### Vernacular name.

Phaya Rak Pa (พญารักป่า), Pan Sieng (พันเซียง).

### Decaneuropsis eberhardtii

(Gagnep.) H.Rob. & Skvarla, Proc. Biol. Soc. Washington 120(3): 365. 2007.

urn:lsid:ipni.org:names:77094685-1:1.1

http://species-id.net/wiki/Decaneuropsis_eberhardtii

Vernonia eberhardtii Gagnep., Bull. Mus. Natl. Hist. Nat.: 489. 1919.
Decaneuropsis eberhardtii Type. Vietnam, Tonkin, *Eberhardt* 4230 (holotype: P!). Fig. 7E.
Vernonia craibiana Kerr, Bull. Misc. Inform. Kew 1935: 328. 1935.
Decaneuropsis eberhardtii Type: Thailand, *A.F.G. Kerr* 9969 (holotype: K!, isotype: L!, isotype: P!).

#### Description.

Climbing or scandent shrubs. Stems sprawling, young branches inconspicuously ribbed, puberulous. Leaves 10–15 by 5–7 cm, elliptic or obovate, margin entire, apex acute, base cuneate, subcoriaceous; both surfaces puberulous with cylindrical hairs, lateral veins 5–7-paired; petioles up to 1 cm long. Capitulescences terminal and axillary, paniculate. Capitula campanulate, 10–15 mm long, pedunculate. Receptacle flat, 2–3 mm in diam., hairy. Involucres campanulate, in 4–5 series, 7–8 mm long, 4–5 mm in diam. Phyllaries imbricate, dark green or purple apically, margin piliferous, outer surface puberulous without glands; the outer and the middle ones ovate, acute or apiculate; the inner ones ovate or ovate-lanceolate, apex acute or obtuse. Florets 11–13; corollas funnelform, purple, glabrous; corolla tubes 5–5.5 mm long; corolla lobes 2–2.5 mm long. Anthers 3–3.5 mm long, apical appendage acute, base acute. Styles purple, 6–6.5 mm long, branches 4–5 mm long. Achenes turbinate, 2.5–3 mm long, 10-ribbed, covered with dense hairs. Pappus in 2 series of bristles, the inner ones 6–7 mm long, persistent.

#### Distribution.

Thailand: Mae Hong Son, Chiang Mai, Lampang, Phetchabun, Nakhon Ratchasima, Chaiyaphum, Kanchanaburi, Yala. Myanmar, Vietnam.

#### Specimens examined.

Thailand, Chaiyaphum, Chulabhorn Dam, 16°32.09'N, 101°38.93'E, 6 Nov 2007, *S. Bunwong* 384 (KKU, US); Mae Hong Son, Pai, 30 may 1977, *T. Santisuk* 1122 (BKF); Chiang Mai, Doi Sutep, 8 Apr 1992, *J.F. Maxwell* 92-129 (E, L); Doi Sutep, 21 Oct 1922, *A.F.G. Kerr* 6413 (K, P); Doi Chiangdao, 21 Mar 1956, *H.B.G. Garrett* 1483 (K, L, P); Lampang, Jae Son, 29 Mar 1996, *J.F. Maxwell* 96-466 (BKF); Jae Son, 1 Feb 1997, *J.F. Maxwell* 97-101 (BKF); Phetchabun, Nam Nao national park, 26 Dec 2007, *S. Bunwong* 376 (KKU, US); Nakhon Ratchasima, Kao Lem, 27 Dec 1980, *Put* 3553 (BK, BM, E, K, P); Kao Lem, 17 Dec 1962, *C. Phengklai* 562 (BKF); Chaiyaphum, 20 Dec 1971, *C.F. van Beusekom, C. Phengklai, R. Geesink & B. Wongwan* 4459 (BKF, C, K, L); Kanchanaburi, Thong Pha Phume, 10 Jan 1985, *H. Koyama, F. Konta & W. Nanakorn* T-48974 (BKF); Thong Pha Phume, 14 Nov 1971, *C.F. van Beusekom, C. Phengklai, R. Geesink & B. Wongwan* 3774 (BKF, C, K, L, P); Yala, Banang Sata, Tal To waterfall, 10 Dec 1966, *B. Sangkhachand* 3064 (BKF).

#### Diagnostic characters.

*Decaneuropsis eberhardtii* differs from *Decaneuropsis cumingiana* by having white hairs on leaves and phyllaries.

#### Ecology.

Dry evergreen forest, alt. 500–800 m; flowering December to February.

#### Vernacular name.

Ya Kaa Krua (ยาแก้เครือ).

### Decaneuropsis garrettiana

(Craib) H.Rob. & Skvarla, Proc. Biol. Soc. Washington 120(3): 365. 2007.

urn:lsid:ipni.org:names:77094686-1:1.1

http://species-id.net/wiki/Decaneuropsis_garrettiana

Vernonia garrettiana Craib, Bull. Misc. Inform., Kew. 1915: 431. 1915.

#### Type.

Thailand, Lampang, Me Maw, *A.F.G. Kerr* 2341 (isotype: BM!, isotype: E!, holotype: K!). Fig. 7F.

#### Description.

Climbing or scandent shrubs, young branches terete, inconspicuously ribbed, puberulous. Leaves 9–25 by 4–15 cm, elliptic or obovate, margin entire, apex acute, base cuneate, chartaceous; upper surface puberulous without glands; lower surface puberulous with whip-shaped hairs, cylindrical hairs and capitate glands, lateral veins 4–11-paired; petioles up to 3.5 cm long. Capitulescences terminal and axillary, paniculate. Capitula campanulate, 12–15 mm long, pedunculate. Receptacle flat, 5–5.5 mm in diam., hairy. Involucres campanulate, in 5–6 series, 10–12 mm long, 8–8.5 mm in diam. Phyllaries imbricate, green, margin piliferous, outer surface puberulous without glands; the outer and the middle ones ovate or ovate-lanceolate, obtuse; the inner ones obovate-lanceolate, apex obtuse. Florets 20–30; corollas funnelform, dark purple, glandular; corolla tubes 7–8 mm long; corolla lobes ca. 2 mm long. Anthers 3–3.5 mm long, apical appendage acute, base acute. Styles white, 7–7.5 mm long, branches 3.5–4 mm long. Achenes subterete, 2.8–3 mm long, 10-ribbed, covered with dense hairs without glands. Pappus in 2 series of bristles, the inner ones 6.5–8.5 mm long, persistent.

#### Distribution.

Thailand: Chiang Mai, Chiang Rai, Lampang, Phrae, Kanchanaburi. Myanmar.

#### Specimens examined.

Thailand, Chiang Mai, Doi Chiangdao wildlife sanctuary, 19°24.11'N, 98°55.10'E, 20 Dec 2003, *S. Bunwong* 75 (KKU); Chiang Mai, Fang, 26 Jan 1973, *S. Sutheesorn* 2287 (BK); Doi Inthanon, 5 Dec 1969, *C.F. van Beusekom & C. Phengklai* 2316 (AAU, BKF, C, E, L, P); Muang District, 14 Feb 1990, *R. Pooma* 386 (BKF); Muang District, 20 Dec 1963, *S. Phusamseang* 66 (BKF, K, L); Muang District, 12 Dec 1997, *W. Pongamornkul* 23 (QBG); Muang District, 24 Dec 1997, *W. Pongamornkul* 48 (QBG); Muang District, 25 May 1995, *W. Nanakorn et al.* 5337 (QBG); Chiang Rai, Chiang kaung, 10 Jan 1922, *H.B.G. Garrett* 130 (BK, BKF, K); Thoeng, 23 Jan 1970, *S. Sutheesorn* 1639 (BK); Kanchanaburi, 15 Jan 1926, *A.F.G. Kerr* 10216 (BK, BM, K).

#### Diagnostic characters.

*Decaneuropsis garrettiana* is distinguished from *Decaneuropsis cumingiana* and *Decaneuropsis eberhardtii* by its obtuse phyllaries.

#### Ecology.

Mixed deciduous, evergreen or pine-oak forest, alt. 400–800 m; flowering November to February.

#### Vernacular name.

Krue Muang Doi (เครือม่วงดอย).

### Elephantopus

L., Sp. Pl.: 814. 1753.

urn:lsid:ipni.org:names:73926-3:1.1

http://species-id.net/wiki/Elephantopus

#### Type.

*Elephantopus scaber* L., Sp. Pl.: 814. 1753.

#### Description.

Annual or perennial herbs. Stems rosulate or caulescent, surface pubescent. Leaves simple, alternate or rosette, usually petiolate; lamina obovate, oblanceolate, elliptic, pilose-villose glandular; margin crenate, dentate or serrate; apex acute or obtuse, base attenuate, usually chartaceous. Capitulescences terminal or axillary, scapose or paniculate. Capitula discoid, tubular; clusters subtended by secondary foliose bracts, homogamous; florets bisexual and fertile. Phyllaries 8, in 2 series, decussate, persistent, oblong, outer surface puberulous or sometimes pilose-villose, glands capitate. Florets 4; corollas purple or white, glabrous or glandular, zygomorphic; corolla tubes slender; corolla lobes 5. Anthers 5, syngenesious, apical appendage acute, anther base not calcarate. Styles purple or white, 2–3-branched, inner surface covered with stigmatic papillae, outer surface covered with sweeping hairs reaching to below style bifurcation. Achenes usually clavate, 10-ribbed, pubescent, carpopodium present. Pappus in 1 series of usually 5 bristles dilated at base. Pollen lophate, 3-porate, without micropuncta.

Two species are recognized in Thailand.

#### Key to the species

**Table:** 

1	Leaves cauline; capitulescences terminal and axillary	*Elephantopus mollis*
–	Leaves rosulate; capitulescences scapiform	*Elephantopus scaber*

### Elephantopus mollis

Kunth in Humb., Bonpl. & Kunth, Nov. Gen. Sp. 4: 26. 1820.

urn:lsid:ipni.org:names:202942-1:1.5

http://species-id.net/wiki/Elephantopus_mollis

Elephantopus tomentosus Koster, Blumea 1: 464. 1935, non L.

#### Type.

**Figure 8.**
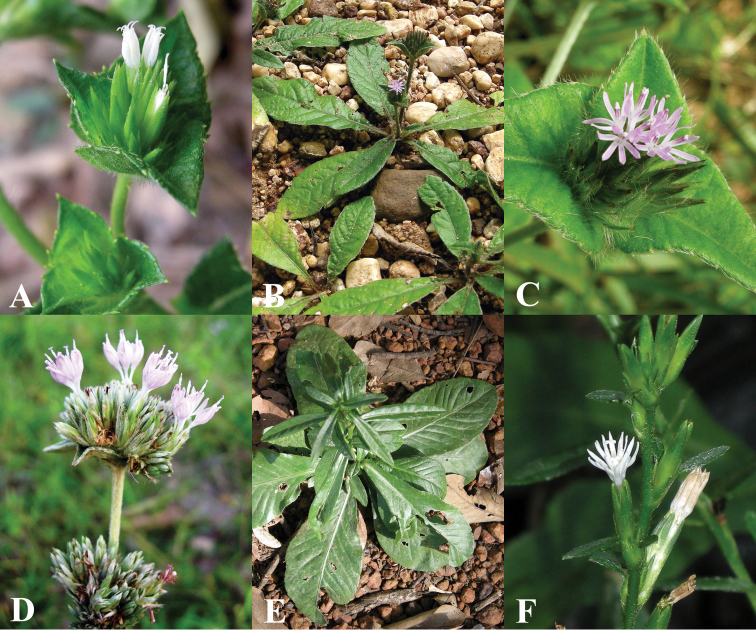
Morphology of *Vernonieae* in Thailand 4. **A** *Elephantopus mollis* **B–C** *Elephantopus scaber* **D** *Elephantopus scaber* var. *penicillatus* **E–F** *Pseudelephantopus spicatus*.

Venezuala, Caracas, *Humboldt & Bonpland* 627 (holotype: P!). Fig. 8A.

#### Description.

Perennial herbs, 0.5-2.0 m tall. Stems caulescent, erect or procumbent, terete, inconspicuously ribbed, pilose. Leaves alternate, 10–20 by 3–5 cm, elliptic or oblong, margin crenate, apex acute, base attenuate, subcoriaceous; both surfaces sparsely pilose with filiform hairs, cylindrical hairs and capitate glands; lateral veins 10–16-paired; petioles up to 10 mm long. Capitulescences terminal and axillary, paniculate. Foliose bracts 3, deltoid. Capitula tubular, 7–8 mm long. Receptacle flat, ca. 1 mm in diam., glabrous. Involucres tubular, 6.5–8 mm long, 3–4 mm in diam. Phyllaries 8, in 2 series, decussate, light green, margin entire, outer surface puberulous, without glands; the outer ovate, apex acute; the inner ones lanceolate, apex acuminate. Florets 4; corollas white, zygomorphic, glabrous; corolla tubes slender, 3–5 mm long; corolla lobes bilabiate, 1.5–2 mm long. Anthers ca. 1 mm long, apical appendage acute, base rounded. Styles white, 4–5 mm long, branches ca. 1 mm long. Achenes clavate, 2.5–3 mm long, pubescent, densely covered with twin hairs but lacking glands, inconspicuously ribbed. Pappus of 5 bristles in one series, dilated at base, 2.5–4.5 mm long.

#### Distribution.

Thailand: Nakhon Ratchasima; Ubon Ratchathani, Satun, Songkhla. Pantropics.

#### Specimens examined.

Thailand, Ubon Ratchathani, Chong Mek border crossing, 15°8.02'N, 105°28.01'E, 27 Oct 2007, *S. Bunwong* 340 (KKU, US); Nakhon Ratchasima, Hui Thaleng, 28 Dec 1928, *Put* 2205 (BM); Satun, 28 Jan 1961, *T. Smitinand* 7194 (BKF); SongKhla, Had Yai, 23 Jan 1986, *J.F. Maxwell* 86-47 (BK, CMU, L).

#### Diagnostic characters.

*Elephantopus mollis* is cleary recognized by its cauline leaves and completely white flowers.

#### Ecology.

Evergreen forest, alt. 0–100 m; flowering October to December.

#### Vernacular name.

Doo Mai Ru Lom (โด่ไม่รู้ล้ม), Hun Huay (หุนหวย).

### Elephantopus scaber

L., Sp. Pl.: 814. 1753.

urn:lsid:ipni.org:names:73955-3:1.1

http://species-id.net/wiki/Elephantopus_scaber

#### Type.

Indiis, *Ana-schovadi* in Rheede, Hort. Malab. 10: 13, t.7. 1690. (Lectotype designated by Jeffrey in Jarvis & al. (ed.), Regnum Veg. 127: 44. 1993. Figs 8B–C.

#### Description.

Perennial herbs. Stems lacking except for the flowering scape, erect, terete, inconspicuously ribbed, sericeous. Leaves in basal rosette at base, obovate or obovate-lanceolate, margin crenate or serrate, apex obtuse or acute, base attenuate, subcoriaceous. Capitulescences terminal, scapose. Foliose bracts 3, deltoid. Capitula tubular. Involucres green or with purple apex. Phyllaries 8, in 2 series, decussate, margin entire or piliferous. Florets 4; corollas salverform, white or purple, zygomorphic. Anthers ca. 2 mm long, apical appendage acute, base acute or rounded. Styles purple. Achenes clavate, 2–3 mm long, pubescent with dense twin hairs, lacking glands. Pappus of 5 bristles with dilated bases in one series, persistent.

Two varieties are recognized in Thailand.

#### Key to the varieties

**Table:** 

1	Leaves and inflorescences densely pilose-tomentose	var. *penicillatus*
–	Leaves and inflorescences sparsely pilose	var. *scaber*

### Elephantopus scaber var. penicillatus

Gagnep., Fl. Indo-Chine [M.H. Lecomte et al.] 3: 503. 1924.

#### Type.

Laos, Chedom, *Thorel* 1407 (holotype: K!). Fig. 8D.

#### Description.

Perennial herbs, 40–100 cm tall. Stems lacking except for inflorescence which is scapose, erect, terete, inconspicuously ribbed and pilose-villose. Leaves in a rosette at base of scape, 6–20 by 6–10 cm, obovate, obovate-lanceolate, elliptic, margin crenate to serrate, apex obtuse, base attenuate, subcoriaceous; upper surface sparsely pilose, without glands; lower surface densely pilose, with filiform cylindrical hairs and capitate glands; lateral veins 7–16-paired; petioles up to 6 cm long. Capitulescences terminal, scapose. Foliose bracts 3, deltoid. Capitula 7–9 mm long. Receptacle flat, ca. 1 mm in diam., glabrous. Involucres tubular, 6.5–8 mm long, 3–4 mm in diam. Phyllaries distichous, decussate, margin entire, outer surface puberulous, glands capitate; the outer ovate-lanceolate, apex acuminate; the inner ones lanceolate or oblong, apex acuminate. Florets 4; corollas salverform, white or purple, zygomorphic, glabrous or rarely hairy; corolla tubes 4–6 mm long; corolla lobes 2.5–3.5 mm long. Anthers ca. 2 mm long, apical appendage acute, base rounded. Styles purple, 6–8 mm long, branches ca. 1 mm long. Achenes 2–2.5 mm long, pubescent with a dense cover of twin hairs, without glands, 10-ribbed. Pappus of 5 bristles ca. 3 mm long, in one series, bristles with dilated bases.

#### Distribution.

Thailand: Loei, Nong Khai, Sakon Nakhon, Nakhon Phanom, Nakhon Ratchasima, Ubon Ratchathani, Chon Buri. Laos, Vietnam.

#### Specimens examined.

Thailand. Udon Thani, Ban Phue district, Phu Phrabat historical park, 17°43.84'N, 102°29.65'E, 29 Sep 2007, *S. Bunwong* 326 (KKU, USLoei, Phu Kra Dung, 17 Aug 1989, *Din* 174 (BKF); Phu Kra Dung, 22 Oct 1990, *Dee* 20 (BKF); Nong Khai, Sang Khom, 16 Dec 1982, *H. Koyama, H. Terao & Th. Wongprasert* T-31139 (BKF); Sakon Nakhon, Phu Phan national park, 5 Oct 2007, *S. Bunwong* 333 (KKU, US); Phu Phan national park, 13 Nov 1984, *G. Murata, C. Phengklai, S. Mitsuta, T. Yahara, H. Nagamasu & N. Nantasan* T-50689 (BKF); Nakhon Phanom, 14 Dec 1982, *H. Koyama, H. Terao & Th. Wongprasert* T-31019 (BKF); Ubon Ratchathani, Route number 2222, 27 Oct 2007, *S. Bunwong* 345 (KKU, US); Kalasin, Sahatsakhan, 22 Oct 1975, *B. Sangkhachand & S. Sutheesorn* 3528 (BK); Nakhon Ratchasima, Bau Yai, 31 Oct 1931, *Put* 4216 (BM, BK, K, L); Chon Buri, Khao Khiew, 19 Oct 1975, *J.F. Maxwell* 75-1040 (AAU, BK, BKF).

#### Diagnostic characters.

*Elephantopus scaber* var. *penicillatus* differs from the typical variety in having large capitula and whitish pilose-tomentose leaf surface and involucres.

#### Ecology.

Dipterocarp or dry evergreen forest, alt. 100–400 m; flowering August to January.

#### Vernacular name.

Doo Mai Ru Lom (โด่ไม่รู้ล้ม), Kee Fai Nok Khum (ขี้ไฟนกคุ้ม).

### Elephantopus scaber var. scaber

#### Description.

Perennial herbs, 10–40 cm tall. Stems lacking except for the flowering scape, erect, terete, inconspicuously ribbed, sericeous. Leaves in basal rosette at base, 8–20 by 3–5 cm, obovate or obovate-lanceolate, margin crenate or serrate, apex obtuse or acute, base attenuate, subcoriaceous; upper surface sparsely pilose without glands, lower surface densely pilose with filiform and cylindrical hairs and capitate glands; lateral veins 12–15-paired; petioles up to 2 cm long. Capitulescences terminal, scapose. Foliose bracts 3, deltoid. Capitula 8–10 mm long. Receptacle flat, ca. 0.5 mm in diam., glabrous. Involucres green with purple apex, 7–10 mm long, 2–3 mm in diam. Phyllaries decussate, margin entire or piliferous, outer surface pilose, without glands; the outer lanceolate, apex acuminate to acuminate; the inner ones oblong, apex acuminate. Florets 4; corollas salverform, purple, zygomorphic, glabrous; corolla tubes 3–3.5 mm long; corolla lobes 1.5–2 mm long. Anthers ca. 2 mm long, apical appendage acute, base acute. Styles purple, 7–8 mm long, branches ca. 0.5 mm long. Achenes 2.5–3 mm long, pubescent with dense twin hairs, lacking glands, inconspicuously ribbed. Pappus of 5 bristles with dilated bases in one series.

#### Distribution.

Thailand: Mae Hong Son, Chiang Mai, Chiang Rai, Nan, Lampang, Sukhothai, Phitsanulok, Phetchabun, Loei, Nong Khai, Sakon Nakhon, Mukdahan, Kalasin, Maha Sarakham, Khon Kaen, Chaiyaphum, Nakhon Ratchasima, Buri Ram, Surin, Ubon Ratchathani, Kanchanaburi, Prachuap Khiri Khan, Chon Buri, Chantaburi, Trat, Chumphon, Ranong, Phangnga, Phuket, Nakhon Si Thammarat, Trang, Satun, Songkhla. Pantropics.

#### Specimens examined.

Thailand, Sakon Nakhon, Phu Phan national park, 17°4.0'N, 103°58.0'E, 6 Oct 2007, *S. Bunwong* 334 (KKU, US); Mae Hong Son, Mae Sariang, 20 Nov 2000, *W. Pongamornkul* 563 (QBG); Chiang Mai, 18 Oct 1979, *T. Shimizu, H. Toyokuni, H. Koyama, T. Yahama & T. Santisuk* T-19344 (BKF); San Kam Phang, 22 Oct 1996, *J.F. Maxwell* 96-1380 (BKF); Doi Inthanon, 16 Dec 1965, *M. Tagawa, K. Iwatsuki & N. Fukuoka* T-2297 (BKF); Mae Rim, 13 Oct 1998, *W. Pongamornkul* 345 (QBG); Chiang Rai, Doi Langka, 20 Dec 1965, *K. Iwatsuki & N. Fukuoka* T3562 (BKF, L), 29 Jun 1997, *T. Smitinand* s.n. (BKF); Nan, Doi Phu Kha national park, 12 Dec 2002, *P. Srisanga* 2618 (QBG); Sukhothai, 4 Nov 1971, *J.F. Maxwell* 71-687 (AAU, BK, BKF); Phetchabun, Nam Nao national park, 26 Dec 1972, *H. Koyama, H. Terao, C. Niyomdham & Th. Wongprasert* T-31745 (BKF); Loei, Na Haew, Phu Suan Sai, 6 Nov 2007, *S. Bunwong* 349 (KKU, US); Phu Rue, 12 Dec 1996, *W. Nanakorn et al.* 8171 (QBG); Mukdahan, Ban Dong Mun, 12 Dec 1982, *H. Koyama, H. Terao, C. Niyomdham & Th. Wongprasert* T-30910 (BKF); Maha Sarakham, Wa Pee Pathum, 31 Oct 1965, *S. Sutheesorn* 697 (BK); Nakhon Ratchasima, Pak Thong Chai, 8 Jun 1982, *Pradit* 601 (BK); Buri Ram, 5 Oct 1984, *G. Murata, C. Phengklai, S. Mitsuta, T. Yahara, H. Nagamasu & N. Nantasan* T-37560 (AAU, BKF); Surin, 8 Jun 1982, *Y. Paisooksantivatana & S. Sutheesorn* 959-82 (BK); Ubon Ratchathani, Phu Pra Bath historical park, 29 Sep 2007, *S. Bunwong* 325 (KKU, US); Kanchanaburi, Si Sa Wat, Arawan, 26 Nov 1982, *H. Koyama, H. Terao, C. Niyomdham & Th. Wongprasert* T-30363 (BKF); Trat, Koh Chang, 22 Oct 1972, *J.F. Maxwell* 72-493 (BK), Koh Good, 20 Oct 2000, *C. Phengklai* 13153 (BKF); Chumphon, Sa Wi, 2 jan 1974, *S. Sutheesorn* 2794 (BK); Nakhon Si Thammarat, Wat Kiriwong, 23 Jan 1966, *M. Tagawa, K. Iwatsuki & N. Fukuoka* T-5382 (BKF); Trang, Khao Chong, 8 Dec 1969, *B. Sangkhachand* 2218 (BK); Songkhla. Had Yai, Thon Nga Chang, 29 Jan 1979, *G. Congdon* 345 (AAU).

#### Diagnostic characters.

This plant is characterized by having basal rosette of leaves and spiciform capitulescence with conspicuous scape. Its leaf shape is variable.

#### Ecology.

Open area in dipterocarp, evergreen or pine-oak forest, alt. 0–300 m; flowering August to January.

#### Vernacular name.

Doo Mai Ru Lom (โด่ไม่รู้ล้ม), Kee Fai Nok Khum (ขี้ไฟนกคุ่ม), Ya Kai Nok Khum (หญ้าไก่นกคุ่ม), Ya Prab (หญ้าปราบ), Ya Sam Sib Song Hab (หญ้าสามสิบสองหาบ), Nat Pha (หนาดผา) Ta Che Go Wa (ตะชีโกวะ), Nat Mee Klan (หนาดมีแคลน).

### Ethulia

L.f., Dec. Pl. Hort. Upsal.: 1. 1762.

urn:lsid:ipni.org:names:8874-1:1.1.2.1.1.1

http://species-id.net/wiki/Ethulia

#### Type.

*Ethulia conyzoides* L.

#### Description.

Annual herbs. Stems erect. Leaves simple, alternate, petiolate; lamina ovate, lanceolate, elliptic, pubescent, margin serrate, apex acute to acuminate, base attenuate, chartaceous. Capitulescences terminal or axillary, corymbose. Capitula discoid, pedunculate, homogamous; florets bisexual and fertile. Involucres campanulate, phyllaries imbricate. Corollas purple, funnelform, glandular; corolla lobes 5, actinomorphic. Anthers 5, syngenesious, apical appendage acute, base obtuse. Styles white, 2-branched, inner surface covered with stigmatic papillae, outer surface covered with sweeping hairs reaching to below style bifurcation. Achenes turbinate, 6-ribbed, glandular, carpopodium absent. Pappus absent. Pollen echinate, tricolporate.

One species is recognized in Thailand.

### Ethulia conyzoides

L., Sp. Pl.: 1171. 1763.

urn:lsid:ipni.org:names:205240-1:1.5

http://species-id.net/wiki/Ethulia_conyzoides

#### Type.

Egypt, *P. Forsskal* 1387 (holotype: K!).

#### Description.

Annual herbs, 50–150 cm tall. Stems erect, conspicuously ribbed, puberulous. Leaves alternate, 5–8 by 1–2 cm, elliptic or lanceolate, margin serrate, apex acuminate or acute, base attenuate, chartaceous; both surfaces ferrugineous with unicellular hairs and capitate glands, shortly petiolate. Capitulescences terminal and axillary, corymbose. Capitula hemispherical, 3–4 mm long. Receptacle ca. 1.5 mm in diam., glabrous. Involucres semispherical, in 3–4 series, 1.5–2 mm long, 1–2 mm in diam. Phyllaries imbricate, green with purple apex, margin piliferous, outer surface puberulous, glands capitate; the outer and the middle ones ovate to lanceolate, apex acute; the inner ones lanceolate to oblong, apex acute. Florets 20–30; corollas funnelform, purple, glandular; corolla tubes 0.5–1 mm long; corolla lobes ca. 1 mm long. Anthers 1.5–2 mm long, apical appendage acute, base obtuse. Styles purple, inner surface covered with stigmatic papillae, sweeping hairs. Achenes turbinate, 1.5–2 mm long, glandular, 6-ribbed. Pappus absent.

#### Distribution.

Thailand: Chiang Rai, Chiang Mai, Nakhon Phanom. Tropics.

#### Specimens examined.

Thailand: Chiang Rai, Mae Kok riverbank, Lamnam Kok national park, 19°57.49'N, 99°41.14'E, 14 Jun 1925, *H.B.G. Garrett* 227 (BKF, BM, K); Nakhon Phanom, 10 May 1932, *A.F.G. Kerr* 21396 (BK, BM, K); Nakhon Phanom, 9 May 1932, *A.F.G. Kerr* 21809 (K).

#### Diagnostic characters.

*Ethulia conizoides* is distinguished by achenes having 4–6 ribs, pappus and carpopodium are absent.

#### Ecology.

Open area along river bank in evergreen forest, alt. 200–400 m; flowering May to September.

#### Vernacular name.

Ya Hua Mud (หญ้าหัวหมุด).

### Gymnanthemum

Cass., Bull. Soc. Philom. Paris 1: 10. 1817.

http://species-id.net/wiki/Gymnanthemum

#### Type.

*Gymnanthemum senegalense* (Pers.) Sch.Bip.

#### Description.

Small trees. Stems caulescent. Leaves simple, alternate, petiolate, lamina ovate, or elliptic, pubescent, margin serrate, apex acuminate, base attenuate, chartaceous. Capitulescences terminal or axillary, corymbose. Capitula discoid, homogamous, pedunculate. Florets bisexual and fertile. Involucre herbaceous, persistent, apex obtuse. Corolla purple to white, actinomorphic; lobes 5. Anthers 5, syngenesious. Styles purple, 2-branched, inner surface covered with stigmatic papillae, outer surface covered with sweeping hairs reaching to below style bifurcation. Achenes subterete or obovate, usually 10-ribbed, carpopodium present. Pappus in 2 series of bristles, persistent, the outer ones are shorter than the inner ones. Pollen subechinolophate, 3-colporate, with prominent micropuncta.

One species is recognized in Thailand.

### Gymnanthemum extensum

(DC.) Steetz, Naturw. Reise Mossambique [Peters] 6(Bot., 2): 337. 1864.

urn:lsid:ipni.org:names:210894-1:1.4

http://species-id.net/wiki/Gymnanthemum_extensum

Figs 9A–B

Vernonia extensa DC., Prodr. 5: 33. 1836.
Conyza extensa Wall., Numer. List [Wallich] no. 3061, comp. no. 126, *nom. nud.*
Gymnanthemum extensum Type. Nepal; *Wallich* 3061 (holotype: G!).
Vernonia cylindriceps C.B. Clarke, J. Linn. Soc. Bot. 25: 35. 1890.
Gymnanthemum cylindriceps (C.B.Clarke) H.Rob., Proc. Biol. Soc. Washington 112(1): 241. 1999.
Gymnanthemum extensum Type: India, Hartook Mekong, *C.B. Clarke* 42109 (holotype: K!).

#### Description.

Shrubs or subshrubs 2–6 m tall. Stems caulescent, young branches inconspicuously ribbed, white puberulous. Leaves 7–13 by 2–4 cm, oblanceolate, margin serrate, apex acute, base attenuate, chartaceous; upper surface puberulous, without glands; lower surface puberulous with whip-shaped hairs, cylindrical hairs and capitate glands; lateral veins 7–12-paired; petioles up to 1 cm long. Capitulescences terminal, corymbose. Capitula narrowly campanulate, 14–16 mm long, pedunculate. Receptacle convex, 1–1.5 mm in diam., hairy. Involucres slightly oblong-cylindrical, in 5–6 series, 8–10 mm long, 3–4 mm in diam. Phyllaries imbricate, green, margin piliferous, outer surface arachnoid without glands; the outer and the middle ones, ovate, apex obtuse; the inner ones lanceolate or ovate-lanceolate, apex obtuse or rounded. Florets 5–10; corollas funnelform, purple or white, glandular, corolla tubes 6–7 mm long; corolla lobes 4–4.5 mm long. Anthers 4–4.5 mm long, apical appendage acute, base obtuse. Styles purple, 9–10 mm long, branches 2, 4–5 mm long. Achenes turbinate, 3–3.5 mm long, 10-ribbed, covered with dense hairs and capitate glands. Pappus in 2 series of bristles, the inner ones 8.5–9 mm long, persistent.

#### Distribution.

Thailand: Chiang Mai, Chiang Rai. China (Yunnan), India, Nepal, Myanmar.

#### Specimens examined.

Thailand, Chiang Mai, Doi Chiangdao wildlife sanctuary, 19°24.11'N, 98°55.10'E, 20 Dec 2002, *S. Bunwong* 76 (KKU); Chiang Mai, Doi Ang Khang, 3 Jan 2008, 19°54.1'N, 99°2.4'E, 3 Jan 2008, *S. Bunwong* 378 (KKU, US); Fang, 11 Feb 1983, *H. Koyama, H. Terao & Th. Wongprasert* T-33345 (BKF); Fang, 12 Feb 1983, *H. Koyama*, *H. Terao & Th. Wongprasert* T-33458 (BKF); Mae Tang, Doi Chang, 12 Feb 1900, *R. Pooma* 382 (BKF); Doi Chang, 18 Jan 1983, *H. Koyama, T. Yahara & W. Nanakorn* T-32726 (BKF, L); Doi Chang, 23 Oct 1979, *T. Shimizu, H. Toyokuni, H. Koyama, T. Yahama & T. Santisuk* 20608 (BKF); Doi Anga Ga, Inthanon, 7 Feb 1931, *H.B.G. Garrett* 629 (BKF, BM, K, P); Doi Inthanon, 22 Mar 1967, *T. Smitinand et al.* 10280 (BK, BKF, K, L, P); Inthanon, 20 May 1970, *Worawoot* 95 (BKF); Doi Chiangdao, 7 Feb 1983, *H. Koyama, H. Terao & Th. Wongprasert* T-33185 (BKF); Chiang Rai, Doi Thung, Mae Sai, 14 Feb 1983, *H. Koyama, H. Terao & Th. Wongprasert* T-33515 (BKF).

#### Diagnostic characters.

This species is characterized by discinctly sweet smell, the habit of shrubs or subshrubs, and obtuse phyllaries.

#### Ecology.

Evergreen or pine-oak forest, alt. 1000–2000 m; flowering December to April.

#### Vernacular name.

Pim Pai Lin (พิมพ์ไพลิน).

### Iodocephalopsis

Bunwong & H.Rob., Proc. Biol. Soc. Washington 122(3): 358. 2009.

urn:lsid:ipni.org:names:77114298-1:1.2

http://species-id.net/wiki/Iodocephalopsis

#### Type.

*Iodocephalopsis eberhardtii* (Gagnep.) Bunwong & H.Rob. Proc. Biol. Soc. Washington 122(3): 358. 2009.

#### Description.

Erect perennial herbs. Stems erect, pubescent with T-shaped hairs. Leaves simple, alternate, petiolate; lamina ovate, pubescent. Capitulescences laxly cymose with capitula solitary or 2 or 3 in a group. Capitula discoid, campanulate, pedunculate, homogamous; florets fertile. Involucre campanulate. Phyllaries imbricate with serrate or entire margins. Florets 15–25; corollas funnelform, purplish or whitish; corolla lobes 5. Anthers 5, purplish or yellowish, syngenesious, exerted. Styles purple, 2-branched, without enlarged basal node, inner surface covered with stigmatic papillae, outer surface and lower style shaft covered with sweeping hairs reaching to below style bifurcation. Achenes 7–10-ribbed, glandular, with vermicular series of idioblasts on the surfaces; achene walls with distinct fibrous layer inside, without raphids, base without carpopodium. Pollen echinolophate, sub-3-colporate, pores in short colpus formed of two partially fused lacunae.

One species is recognized in Thailand.

### Iodocephalopsis eberhardtii

(Gagnep.) Bunwong & H.Rob., Proc. Biol. Soc. Washington 122(3): 358. 2009.

urn:lsid:ipni.org:names:77114298-1:1.2

http://species-id.net/wiki/Iodocephalopsis_eberhardtii

**Figure 9.**
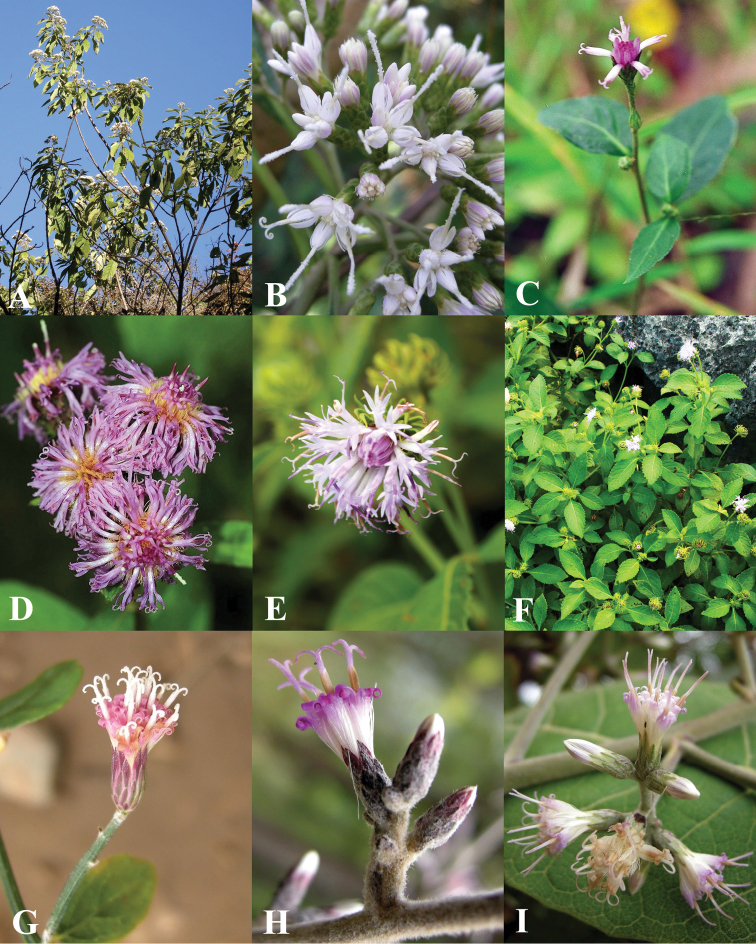
Morphology of *Vernonieae* in Thailand 5. **A–B** *Gymnanthemum extensum* **C** *Iodocephalopsis eberhardtii* **D** *Koyamasia calcarea* **E–F** *Koyamasia curtisii* **G** *Kurziella gymnoclada* **H** *Monosis parishii* **I** *Monosis volkameriifolia*

Iodocephalus eberhardtii Gagnep., Notul. Syst. (Paris) 4: 18. 1920.
Camchaya eberhardtii (Gagnep.) Kitam., Acta Phytotax. Geobot. 23: 71. 1968.
Iodocephalopsis eberhardtii Type. Vietnam, Annam, Lang-bian, *Eberhardt* 1711 (holotype: P!). Fig. 9C.
Iodocephalus glandulosus Kerr, Kew Bull.: 326. 1935.
Iodocephalopsis eberhardtii Type: Thailand, Chiang Mai, Doi Sutep, *A.F.G. Kerr 789* (holotype: BM!).

#### Description.

Perennial herbs, 0.5–1 m tall. Stems erect, terete, inconspicuously ribbed, puberulous with T-shaped hairs and glands. Leaves alternate, obovate-lanceolate, 4–10 by 1–4 cm, margin serrate or entire, apex acute, base attenuate, chartaceous; both surfaces puberulous with whip-shaped hairs, cylindrical hairs and capitate glands; lateral veins 5–8-paired; petioles up to 3 mm long. Capitulescences terminal or axillary, corymbose or solitary. Capitula campanulate, 8–10 mm long, pedunculate. Receptacle convex, 2.5–4 mm in diam., glabrous. Involucre campanulate, in 3–4 series, 6–7 mm long. Phyllaries imbricate, dull green, margin piliferous, outer surface arachnoid, glandular; the outer and the middle ones ovate, apex acute to acuminate; the inner ones ovate-lanceolate, apex acute to acuminate. Florets 15–25; corollas funnelform, purple or white, puberulous, glands capitate; corolla tubes 2.5–4 mm long; corolla lobes 2–2.5 mm long. Anthers ca. 2 mm long, apical appendage acute, base rounded. Styles purple, 3–5 mm long, branches 1–2 mm long. Achenes turbinate, 3–5 mm long, glandular, 7–10-ribbed. Pappus absent.

#### Distribution.

Thailand: Chiang Mai, Chiang Rai, Nan, Phitsanulok, Loei, Khon Kaen, Nakhon Ratchasima, Phetchaburi, Nakhon Nayok. Laos, Myanmar.

#### Specimens examined.

Thailand, Chiang Mai, Doi Sutep Pui national park, 18°48.39'N, 98°54.90'E, 8 Oct 2007, *S. Bunwong* 335 (KKU, US); Doi Sutep Pui national park, 4 Oct 1986, *Y. Paisooksantivatana* 1857-86 (BK); Doi Sutep Pui national park, 14 Oct 1979, *T. Shimizu, H. Toyokuni, H. Koyama, T. Yahama & T. Santisuk* T-18670 (L); Loei, Phu Reau, 23 Dec 1982, *H. Koyama, H. Terao, Th. Wongprasert* T-31570 (BKF, L); Phu Luang, 24 Dec 1982, *H. Koyama, H. Terao, Th. Wongprasert* T-30837 (BKF); Phu Kra Dung, 18 Jan 1991, *Din* 6 (P); Phu Kra Dung,1 Nov 1984, *G. Murata, C. Phengklai, S. Mitsuta, T. Yahara, H. Nagamasu & N. Nantasan* T-42599 (L); Phu Kra Dung, 31 Oct 1984, *S. Mitsuta, H. Nagamasu, T. Yahara & N. Nantasan* T-42260 (BKF); Khon Kaen, Phu Khiew, 7 Nov 1984, *G. Murata, K. Iwatsuki, C. Phengklai & C. Charenphol* T41669 (BKF); Nakhon Nayok, Khao Yai, 12 Aug 1974, *J.F. Maxwell* 74-808 (BK).

#### Diagnostic characters.

*Iodocephalopsis eberhardtii* is different from *Camchaya* spp. by having 3-colporate pollen, no pappus and phyllaries without marginal spines.

#### Ecology.

Edge of evergreen and pine-oak forests, alt. 700–1700 m; flowering August to December.

#### Vernacular name.

Muang Cha Rad (ม่วงจรัส).

### Koyamasia

H.Rob., Proc. Biol. Soc. Washington 112(1): 234. 1999.

urn:lsid:ipni.org:names:1010933-1:1.1.2.1.1.1

http://species-id.net/wiki/Koyamasia

#### Type.

*Camchaya calcarea* Kitam., Acta Phytotax. Geobot. 23: 71. 1968.

#### Description.

Perennial herbs. Stems erect, pubescent. Leaves simple, alternate, petiolate, lamina ovate or elliptic, margin serrate, apex acute or acuminate, base attenuate. Capitulescences terminal, solitary or paniculate. Capitula discoid, homogamous; pedunculate, florets bisexual and fertile. Involucre imbricate, phyllary tips recurved. Florets 60–100; corollas purplish or white, actinomorphic; corolla lobes 5. Anthers 5, syngenesious. Styles 2-branched, inner surface covered with stigmatic papillae, outer surface covered with sweeping hairs reaching to below style bifurcation. Achenes subterete, 10-ribbed, carpopodium absent. Pappus in one series of bristles, deciduous. Pollen echinolophate, 3-porate, without micropuncta.

Three species are recognized in Thailand.

#### Key to the species

**Table:** 

1	Capitula broadly campanulate, 15–30 mm long, involucres hemispherical, 15–20 mm long, 15–20 mm in diam., florets ca. 100	*Koyamasia calcarea*
–	Capitula campanulate, 15–20 mm long, involucres campanulate, 7–10 mm long, 8–15 mm in diam., florets ca. 60	*Koyamasia curtisii*

### Koyamasia calcarea

(Kitam.) H.Rob., Proc. Biol. Soc. Washington 112(1): 235. 1999.

urn:lsid:ipni.org:names:1010934-1:1.1.2.1.1.2

http://species-id.net/wiki/Koyamasia_calcarea

Camchaya calcarea Kitam., Acta Phytotax. Geobot. 23: 71. 1968.
Vernonia calcarea (Kitam.) H.Koyama, Bull. Natn. Sci. Mus. Tokyo, Ser. B 29(1): 20. 2003.

#### Type.

Thailand, Chiang Mai, Doi Chiangdao, *T. Shimizu, H. Koyama & A. Nalampoon* T-10011 (holotype: KYO!). Fig. 9D.

#### Description.

Perennial herbs, 20–80 m tall. Stems erect, conspicuously ribbed, puberulous with stipitate glands. Leaves 10–30 by 3–6 cm, ovate or elliptic, margin serrate, apex acute to acuminate, base attenuate, subcoriaceous, upper surface scabrous without glands, lower surface scabrous with whipshaped hairs, and capitate glands, lateral veins 7–10-paired; petioles up to 6 cm long. Capitulescences terminal, usually solitary. Capitula broadly campanulate, 1.5–3 cm long, pedunculate. Receptacle glabrous. Involucres hemispherical, in 6–7 series, 15–20 mm long, 15–20 mm in diam. Phyllaries imbricate, green or purple, margin entire, outer surface puberulous glandular; the outer and the middle ones ovate to lanceolate, acuminate or aristate, upper half strongly reflexed; the inner ones ovate-lanceolate, apex acute. Florets more than 80, corollas funnelform, purple or white, glandular, corolla tubes 5–6 mm long; corolla lobes 2–3 mm long. Anthers 3–4 mm long, apical appendage acute, base obtuse. Styles purple. Achenes subterete, 3.5–4.5 mm long, 10-ribbed, glabrous. Pappus in 1 series of bristles, 1–3 mm long, deciduous.

#### Distribution.

Thailand: Chiang Mai. Endemic.

#### Specimens examined.

Thailand. Chiang Mai, Chiangdao wildlife sanctuary, 19°26.08'N, 98°53.76'E, 15 Nov 1963, *Adisai* 611 (BK); Chiangdao wildlife sanctuary, 3 Nov 1922, *A.F.G. Kerr* 6548 (BM, BK, K); Chiangdao wildlife sanctuary, 27 Oct 1979, *T. Shimitzu, H. Toyokuni, H. Koyama, T. Yahara, T. Santisuk & C. Niyomdham* T-21121 (L); Chiangdao wildlife sanctuary, *T. Shimitzu, H. Toyokuni, H. Koyama, T. Yahara, T. Santisuk & C. Niyomdham* T-21128 (L); Chiangdao wildlife sanctuary, 27 Oct 1979, *T. Shimitzu, H. Toyokuni, H. Koyama, T. Yahara, T. Santisuk & C. Niyomdham* T-21181 (L).

#### Diagnostic characters.

*Koyamasia calcarea* can be distinguished by its oblong achenes, 3-porate pollen and reflexed phyllaries. Its capitula are also larger than those of *Koyamasia curtisii*.

#### Ecology.

Restricted to limestone mountain, altitude ca. 1300 m from sea level; flowering October to December.

#### Vernacular name.

Akkanee Thewa (อัคคนีเทวา).

### Koyamasia curtisii

(Craib & Hutchinson) Bunwong, Chantar. & S.C.Keeley comb. nov.

urn:lsid:ipni.org:names:77138474-1

http://species-id.net/wiki/Koyamasia_curtisii

Vernonia curtisii Craib & Hutchinson, Bull. Misc. Inform. Kew 1910: 22. 1910.

#### Type.

Malay Peninsula, Kedah, Langawi, *Curtis* 2127 (holotype: K!). Figs 9E–F.

#### Key to the varieties

**Table:** 

1	Lower surface of leaves puberulous	var. *curtisii*
–	Lower surface of leaves tomentose	var. *tomentosa*

### Koyamasia curtisii var. curtisii

#### Description.

Herbs 20–100 cm tall. Stems erect, conspicuously ribbed, puberulous with stipitate glands. Leaves 5–15 by 2–7 cm, ovate or elliptic, margin serrate, apex acute to acuminate, base attenuate, chartaceous; both surfaces puberulous with whip-shaped hairs and capitate glands; lateral veins 7–12-paired; petioles up to 4 cm long. Capitulescences terminal, solitary or loosely paniculate. Capitula campanulate, 15–20 mm long, pedunculate. Receptacle flat, glabrous. Involucres campanulate, 7–10 mm long, 8–15 mm in diam. Phyllaries imbricate, in 6–7 series, light green or purple apex, margin entire, outer surface puberulous; the outer and the middle ones ovate to lanceolate, apex acuminate with reflexed, the inner ones lanceolate to oblong, apex caudate. Florets ca. 60; corollas funnelform, purple, pubescent with soft hairs and capitate glands; corolla tubes 7–10 mm long; corolla lobes 2–3 mm long. Anthers 2.8–3 mm long, apical appendage acute, base obtuse. Styles purple. Achenes clavate, 3–3.5 mm long, 10-ribbed, sparsely glandular. Pappus in one series of bristles, 2–8 mm long, deciduous.

#### Distribution.

Thailand: Lampang, Phetchabun, Kanchanaburi, Prachuap Khiri Khan, Saraburi, Phatthalung, Trang, Satun, Songkla. India, Laos, Myanmar, Vietnam, Malay Peninsula, Malay islands.

#### Specimens examined.

Thailand, Prachuap Khiri Khan, Kuiburi district, 12°3.44'N, 99°37.59'E, 19 Nov 1964, *Adisai* 965 (BK); Satun, Tarutao national park, 6°36.19’N, 99°39.15’E, 21 Apr 1969, *C. Chermsirivathana* 1477 (BK, L); Lampang, Muang Ngao, 17 Jan 1931, *Put* 4019 (AAU, BK, BM, K, P); Muang Ngao,30 Aug 1925, *Winit* 757 (K); Kanchanaburi, Si Sa Wat, 10 Aug 1967, *Kasem* 548 (BK); Si Sa Wat, 30 Jul 1925, *A. Marcan* 2348 (BM, K, P); Si Sa Wat, 31 Jul 1928, *Put* 1780 (AAU, BK, K, L, P); Saraburi, Muak Lek, 4 Sep 1925; Muak Lek, *Put* 1877 (BK),; Muak Lek, 4 Sep 1928, *Put* 1879 (AAU, BK, BM, E, K, P); Muak Lek, 4 Sep 1963, *T. Smitinand & H. Sleumer* 1372 (K, L); Trang, Kao Kao, 2 Aug 1929, *Rabil* 310 (BK, BM, K).

#### Diagnostic characters.

*Koyamasia curtisii* is similar to *Koyamasia calcarea* in having solitary or loosely paniculate capitulescences and reflexed phyllaries but differs in having smaller capitula and a pappus. Both species are found in limestone mountains.

#### Ecology.

Limestone mountain, alt. 100–500 m; flowering November to April.

#### Vernacular name.

Chang Nga Pha (ช้างงาผา).

### Koyamasia curtisii var. tomentosa

(Kerr) Bunwong, Chantar. & S.C.Keeley comb. nov.

urn:lsid:ipni.org:names:77138506-1

Vernonia curtisii var. *tomentosa* Kerr, Fl. Siam Enum. 2(3): 238. 1936.

#### Type.

Thailand, Ratchaburi, *A.F.G. Kerr* 8997 (isotype: BK!, isotype: BM!, holotype: K!).

#### Description.

Herbs 20–100 cm tall. Stems erect, conspicuously ribbed, tomentose or villose. Leaves 5–10 by 2–6 cm, ovate or elliptic, margin serrate, apex acute to acuminate, base attenuate, chartaceous; upper surface scabrous; lower surface tomentose; lateral veins 7–12-paired; petioles up to 4 cm long. Capitulescences terminal, solitary or paniculate. Capitula campanulate, 11–13 mm long, pedunculate. Receptacle flat, glabrous. Involucres campanulate, in 6–7 series, 7–8 mm long, 8–10 mm in diam. Phyllaries imbricate, green with purple apex, margin piliferous, outer surface puberulous; the outer and the middle ones ovate to lanceolate, caudate, upper half strongly reflexed; the inner ones lanceolate to oblong, apex acute to acuminate. Florets ca. 60; corollas funnelform, purple; corolla tubes 7–8 mm long; corolla lobes 2–2.5 mm long. Anthers 2.8–3 mm long, apical appendage acute, base obtuse. Styles purple. Achenes clavate, 3–3.5 mm long, 10-ribbed, sparsely glandular. Pappus in 2 series of bristles, the inner ones 5–6 mm long, deciduous.

#### Distribution.

Thailand: Chiang Mai, Chiang Rai, Phayao, Kanchanaburi, Ratchaburi. Endemic.

#### Specimens examined.

Thailand. Chiang Mai, Chiangdao wildlife sanctuary, 19°26.08'N, 98°53.76'E, 25 Jul 1990, *H. Banziger* 702 (CMU); Chiang Rai, Doi Thung, 27 Aug 2000, *S. Watthana* 875 (AAU, QBG); Phayao, Pak Bok, 5 Sep 2006, *S. Pumicong* 443 (QBG); Kanchanaburi, 16 Aug 1971, *C. Phengklai, B. Sangkhachand & B. Nimanong* 2986 (K).

#### Diagnostic characters.

This plant differs from the typical variety by having tomentose hairs on the lower leaf surfaces.

#### Ecology.

Limestone mountain, alt. 200–1600 m; flowering June to August.

#### Vernacular name.

Hua Chai Wai Yarap (หัวใจไวยราพณ์).

### Kurziella

H.Rob. & Bunwong, Proc. Biol. Soc. Washington 123(2): 176. 2010.

urn:lsid:ipni.org:names:77114714-1:1.2

http://species-id.net/wiki/Kurziella

#### Type.

*Vernonia gymnoclada* Collett & Hemsl.

#### Description.

Perennial herbs. Stems erect, young branches angled, puberulous. Leaves simple, alternate, subsessile, pubescent, glands capitate, subcoriaceous absent at anthesis. Capitulescences axillary, spicate or solitary. Capitula discoid, homogamous, sessile or subsessile, florets bisexual and fertile. Involucres campanulate. Phyllaries imbricate, persistent, without glands. Corollas purple, actinomorphic, lobes 5. Anthers 5, syngenesious. Styles 2-branched, inner surface covered with stigmatic papillae, outer surface covered with sweeping hairs. Achenes terete, carpopodium present, hairy without glands. Pappus in 1 series of bristles. Pollen echinate, 3-colporate, with micropuncta.

One species is recognized in Thailand.

### Kurziella gymnoclada

(Collett & Hemsl.) H.Rob & Bunwong, Proc. Biol. Soc. Washington 123(2): 177. 2010.

urn:lsid:ipni.org:names:77114715-1:1.2

http://species-id.net/wiki/Kurziella_gymnoclada

Vernonia gymnoclada Collett & Hemsl., J. Linn. Soc., Bot. 28: 70. 1890.
Vernonia juncea Hook.f., Fl. Br. Ind. 3: 231. 1881, *nom. nud.*
Kurziella gymnoclada Type. Myanmar, Meiktila, *H. Collet* 515 (holotype: K!). Fig. 9G.

#### Description.

Perennial herbs, up to 1 m tall. Stems erect, conspicuously ribbed, puberulous. Leaves 1–3 by 1–2 cm, obovate, margin serrate, apex obtuse or truncate, base cuneate, subcoriaceous, both surfaces scabrous with whip-shaped hairs and capitate glands, lateral veins 2–3-paired; petioles up to 5 mm long. Capitulescences terminal and axillary, spicate or solitary. Capitula campanulate, 10–13 mm long, subsessile or shortly pedunculate. Receptacle flat, 1.5–2 mm in diam. Involucres campanulate. Phyllaries imbricate, in 5–6 series, 8–10 mm long, 4–5 mm in diam., green or purple apically, margin piliferous, outer surface puberulous without glands; the outer and the middle ones ovate or lanceolate, acute; the inner ones lanceolate to oblong, apex acute. Florets 15–20; corollas funnelform, purple, glabrous, corolla tubes 8–10 mm long; corolla lobes 3–3.5 mm long. Anthers 2–2.5 mm long, apical appendage acute, base obtuse. Styles purple, 7–8 mm long, branches 2–2.5 mm long. Achenes 2–3 mm long, ca. 5-ribbed, covered with dense hairs. Pappus in 1 series of brisitles, 9–10 mm long.

#### Distribution.

Thailand: Kamphaeng Phet, Khon Kaen, Nakhon Ratchasima, Kanchanaburi, Ratchaburi, Phetchaburi, Prachuap Khiri Khan, Chai Nat, Saraburi, Bangkok. Myanmar.

#### Specimens examined.

Thailand, Khon Kaen, Phon district, 15°48.96'N, 102°35.91'E, 28 Feb 2008, *S. Bunwong* 391 (KKU, US); Nakhon Ratchasima, 21 Jan 1931, 21 Jan 1931, *A.F.G. Kerr* 19911 (AAU, BK, BM, E, K); Nakhon Ratchasima, 3 Mar 1958, *Th. Sørensen, K. Larsen & B. Hansen* 2166 (BKF, C, K); Kanchanaburi, 13 Mar 1926, *A.F.G. Kerr* 10618 (BK, BM, C, E, K); Kanchanaburi, 18 Jan 1929, *Put* 2273 (AAU, BK, BM, E, K); Kanchanaburi, 21 Dec 1970, *T. Smitinand* 11398 (BKF); Chai Nat, 8 Jan 1980, *Put* 2654 (BK, BM, E, L, K); Saraburi, Minam Sak, 3 May 1923, *A.F.G. Kerr* 7029 (AAU, BK, BM, E, K); Ratchaburi, Kao Tum Pha, 15 Mar 1965, *S. Sutheesorn* 479 (BK); Phetchaburi, Kao Ec San, 8 Mar 1965, *S. Sutheesorn* 478 (BK).

#### Diagnostic characters.

*Kurziella gymnoclada* is distinguished by a single row of persistent pappus, deciduous leaves and sessile or subsessile capitula in axillary leaves.

#### Ecology.

Dipterocarp forest, alt. 40–200 m; flowering November to March.

#### Vernacular name.

Yoong Pad Maa Mai (ยุ้งปัดแม่หม้าย).

### Monosis

DC. in Wight, Contrib. Bot. Ind. 5. 1834.

urn:lsid:ipni.org:names:30071981-2:1.1.2.1

http://species-id.net/wiki/Monosis

#### Type.

*Monosis wightiana* DC., Contr. Bot. India [Wight]: 5. 1834.

#### Description.

Perennial plants. Stems small trees or shrubs, young branches terete, tomentose. Leaves simple, alternate, petiolate, pubescent with flagellate hairs, lamina ovate, obovate, oblanceolate or elliptic, margin serrate, apex acute, base attenuate or cuneate, subcoriaceous. Capitulescences terminal, thyrsoid paniculate. Capitula discoid, homogamous, pedunculate, florets bisexual and fertile. Involucre campanulate, in 4–5 series, 4–5 mm long. Phyllaries imbricate, persistent, without glands. Corollas funnelform, purple, actinomorphic, corolla lobes 5. Anthers 5, syngenesious. Styles 2-branched, inner surface covered with stigmatic papillae, outer surface covered with sweeping hairs on the outer surface reaching below style bifurcation. Achenes turbinate, 10-ribbed, carpopodium present, hairy with glands. Pappus in 2 series of bristles, persistent, the outer ones are shorter than the inner ones. Pollen lophate with high muri.

Two species are recognized in Thailand.

#### Key to the species

**Table:** 

1	Shrubs; lower leaf surface and young shoot ferruginous tomentose; Achenes 2.5–3.5 mm long	*Monosis parishii*
–	Small tree; lower leaf surface and young shoot whitish puberulous; Achenes 4–5 mm long	*Monosis volkameriifolia*

### Monosis parishii

(Hook.f.) H.Rob. & Skvarla, Proc. Biol. Soc. Washington 119(4): 605. 2006.

urn:lsid:ipni.org:names:60452500-2:1.1

http://species-id.net/wiki/Monosis_parishii

Vernonia parishii Hook.f., Fl. Br. Ind. 3: 240. 1882.

#### Type.

Myanmar, Attran, *Parish* 103 (holotype: K!). Fig. 9H.

#### Description.

Shrubs or subshrubs, 1–3 m tall. Stems erect, young branches inconspicuously ribbed, ferruginous tomentose. Leaves 10–26 by 3–11 cm, ovate or elliptic, margin serrate, apex acute, base attenuate, coriaceous; upper surface ferruginous puberulous without glands; lower surface ferruginous tomentose with flagellate hairs and capitate glands; lateral veins 11–13-paired; petioles up to 3 cm long. Capitulescences terminal, thyrsoid paniculate. Capitula narrowly campanulate or slightly oblong-cylindrical, 6–7 mm long, subsessile or pedunculate. Receptacle flat, 2–3 mm in diam., glabrous. Involucres narrowly campanulate, in 4–5 series, 4–5 mm long, 2.5–3 mm in diam. Phyllaries imbricate, purple or green with purple apex, margin piliferous, outer surface arachnoid without glands; the outer and the middle ones ovate, apex acute or obtuse; the inner ones lanceolate or oblong, apex acute. Florets 7–9; corollas funnelform, purple, glandular, corolla tubes 4–5 mm long; corolla lobes 2–2.5 mm long. Anthers 3–3.5 mm long, apical appendage acute, base obtuse. Styles purple, 4–5.5 mm long, branches 2, 2–2.5 mm long. Achenes turbinate, 2.5–3.5 mm long, 10-ribbed, covered with sparse hairs and capitate glands. Pappus in 2 series of bristles, the inner ones 5–6 mm long, persistent.

#### Distribution.

Thailand: Mae Hong Son, Chiang Mai, Chiang Rai, Lampang, Sukhothai, Kanchanaburi. China (Yunnan), India, Myanmar, Laos.

#### Specimens examined.

Thailand, Chiang Mai, Doi Sutep Pui national park, 18°48.39'N, 98°54.90'E, 1 Mar 2002, *S. Bunwong* 66 (KKU); Mae Rim, 1 Mar 2008, *S. Bunwong* 394 (KKU, US); Mae Hong Son, Mae Sariang, 15 Apr 1973, *S. Sutheesorn* 2337 (BK); Mae Sariang, 17 Jan 1983, *H. Koyama, H. Terao & Th. Wongprasert* T-32677 (BKF); Chiang Mai, Pang Tawn, 30 Apr 1981, *Put* 4527 (BK, BM, E, K, L, P); Thoeng, Ban Miya, 10 Feb 1970, *S. Sutheesorn* 1634 (BK); Doi Chiangdao, 14 Jan 1973, *S. Sutheesorn* 2272 (BK); Doi Chiangdao, 2 Dec 1984, *H. Koyama, S. Mitsuta & H. Nagamasu* T-39776 (BKF); Fang, Doi Ang Khang, 6 Jun 1973, *J. Sadakorn* 231 (BK); Doi Sutep, 11 Mar 1984, *L.K. Juaton* 126 (BK); Doi Sutep, 2 Mar 1966, *C. Chermsirivathana* 416 (BK); Doi Inthanon, 6 Dec 1984, *S. Mitsuta, T. Yahara & H. Nagamasu* 46455 (BKF); Doi Inthanon, 7 Dec 1998, *F. Konta, C. Niyomdham & S. Khao-iam* 4347 (BKF); Doi Inthanon, 17 Feb 1998, *C. Niyomdham* 5311 (BKF); Doi Inthanon, 15 Nov 1969, *Worawoot* 4 (BKF); Doi Inthanon, 27 Feb 1979, *T. Koyama, C. Phengklai, C. Niyomdham, H. Okada & P.J. O’Connor* T-15,579 (AAU, BKF); Chiang Rai, Wieng Paa Pao, 2 Apr 1998, *J.F. Maxwell* 89-487 (BKF, L); Lampang, Khun Than, 28 Dec 1984, *H. Koyama, C. Phengklai* T-39152 (BKF); Sukhothai, Kao Luang Khirimat, 24 Jan 1990, *Parikarn & Prayad* 168 (BK); Kanchanaburi, Thong Pha Phume, 17 Dec 1961, *C. Phengklai* 215 (BKF); Thong Pha Phume, 22 Dec 1961, *C. Phengklai* 303 (BKF).

#### Diagnostic characters.

*Monosis parishii* is recognized by its ferruginuous tomentose leaf surface and the shrubby habit.

#### Ecology.

Hill evergreen or pine-oak forest, alt. 250–800 m; flowering December to April.

#### Vernacular name.

Khang Hang Lek (ขางหางเล็ก), Tree Cha Wa (ตรีชะวา), Nat Ngern (หนาดเงิน).

### Monosis volkameriifolia

(DC.) H.Rob. & Skvarla, Proc. Biol. Soc. Washington 119(4): 606. 2006.

urn:lsid:ipni.org:names:60452504-2:1.1

http://species-id.net/wiki/Monosis_volkameriifolia

Conyza volkameriifolia Wall., Numer. List [Wallich] no. 3001, comp. no. 111, *nom. nud.*
Vernonia volkameriifolia DC., Prodr. 5: 32. 1836.

#### Type.

Nepal, *Wallich* 3001 (holotype: K!). Fig. 9I.

#### Description.

Small tree, 3–6 m tall. Stems erect, bark grey, young branches inconspicuously ribbed, white tomentose. Leaves alternate, 10–50 by 5–20 cm, obovate or oblanceolate, margin serrate, apex acute, base cuneate, coriaceous; upper surface whitish puberulous, without glands; lower surface whitish puberulous with flagellate hairs and capitate glands; lateral veins 10–20-paired; petioles up to 3 cm long. Capitulescences terminal, thyrsoid paniculate. Capitula campanulate, 9–10 mm long, subsessile or shortly pedunculate. Receptacle flat, 1.5–2 mm in diam., glabrous. Involucres narrowly campanulate or slightly oblong-cylindrical, in 4–5 series. Phyllaries imbricate, light green or purple apically, 4–5 mm long, 2–3 mm in diam., margin piliferous or entire, outer surface white arachnoid without glands; the outer and the middle ones ovate, apex acute; the inner ones ovate or lanceolate, apex acute. Florets 8–10; corollas funnelform, purple, glandular; corolla tubes 5.5–6 mm long; corolla lobes 2–3 mm long. Anthers 3–3.5 mm long, apical appendage acute, base acute. Styles purple, 5–7 mm long, branches 2, 3–4 mm long. Achenes turbinate, 4–5 mm long, 10-ribbed, covered with dense twin hairs and capitate glands. Pappus in 2 series of bristles, the inner ones 7–8 mm long.

#### Distribution.

Thailand: Mae Hong Son, Chiang Mai, Lamphun, Lampang. China (Yunnan), India, Bhutan, Myanmar, Vietnam.

#### Specimens examined.

Thailand, Chiang Mai, Doi Sutep Pui national park, 18°48.39'N, 98°54.90'E, 9 Dec 2007, *S. Bunwong* 362 (KKU, US); Mae Rim, 1 Mar 2008, *S. Bunwong* 393 (KKU, US); Mae Rim, 2 Apr 1925, *Winit* 1329 (BK, K); Doi Chiangdao, 5 Nov 1961, *K. Bunchuai* 110 (BKF); Doi Chiangdao, 7 Jan 1975, *R. Geesink, P. Hiepko & C. Phengklai* 8160 (AAU, BKF, L); Doi Chiangdao,9 Feb 1983, *H. Koyama, H. Terao & Th. Wongprasert* T-33277 (BKF); Doi Chiangdao, 4 Mar 1995, *J.F. Maxwell* 95-212 (BKF, L); Doi Chiangdao, 26 Oct 1979, *T. Shimitzu, H. Toyokuni, H. Koyama, T. Yahara & T. Santisuk* T-20886 (BKF, L); Doi Sutep, 16 Feb 1983, *H. Koyama, H. Terao & Th. Wongprasert* T-33616 (BKF); Doi Sutep, 10 Jan 1958, *Th. Sørensen, K. Larsen & B. Hansen* 6604 (BKF, C, K); Doi InThanon, 22 Nov 1964, *K. Bunchuai* 1397 (BKF, K, L, P); Doi InThanon, 28 Nov 1930, *H.B.G. Garrett* 617 (AAU, BKF, K, L, P); Mae Hong Son, Pai, 16 Jan 1983, *H. Koyama, H. Terao & Th. Wongprasert* T-32615 (BKF); Lampang, Doi Khun Than, 27 Dec 1984, *H. Koyama & C. Phengklai* T-39091 (BKF, L); Doi Khun Than,2 Jan 1985, *H. Koyama & C. Phengklai* T-39196 (AAU, BKF, L).

#### Diagnostic characters.

*Monosis volkameriifolia* is similar to *Monosis parishii* in capitula and leaf shape but differs in tree habits and whitish puberulous leaf surfaces.

#### Ecology.

Hill evergreen or pine-oak forest, alt. 500–1750 m; flowering November to March.

#### Vernacular name.

Kla Po Pha Du (คละปอพะดุ), Ya Kaa (หญ้าแก้), Ma Hok Ton (มะโหกต้น), Yarn (หยาน).

### Okia

H.Rob. & Skvarla, Proc. Biol. Soc. Washington 123(1): 88. 2010.

urn:lsid:ipni.org:names:77114254-1:1.2

http://species-id.net/wiki/Okia

#### Type.

*Cacalia birmanica* Kuntze.

#### Description.

Annual herbs. Stems erect, inconspicuously ribbed, puberulous. Leaves cauline, lanceolate, margin serrate. Capitulescences terminal, loosely paniculate. Capitula campanulate, peduncles fistulose. Phyllaries imbricate, subglobular. Florets 20–55; corollas funnelform, white or purple. Achenes subterete, 10-ribbed, aculeate between ribs, carpopodium present. Pappus in 2 series of bristles, persistent, the outer ones are shorter than the inner ones. Pollen lophate, 3-porate, without micropuncta.

Two species are recognized in Thailand.

#### Key to the species

**Table:** 

1	Capitula 10–12 mm long, florets ca. 55, inner pappus ca. 7 mm long	*Okia birmanica*
–	Capitula 5–6 mm long, florets ca. 20, inner pappus ca. 5 mm long	*Okia pseudobirmanica*

### Okia birmanica

(Kuntze) H.Rob. & Skvarla, Proc. Biol. Soc. Washington 123(1): 91. 2010.

urn:lsid:ipni.org:names:77114255-1:1.2

http://species-id.net/wiki/Okia_birmanica

Vernonia birmanica (Kuntze) Merr., Brittonia 2: 200. 1936.
Cacalia birmanica Kuntze, Rev. Gen. P1.: 323. 1891.

#### Type.

**Figure 10.**
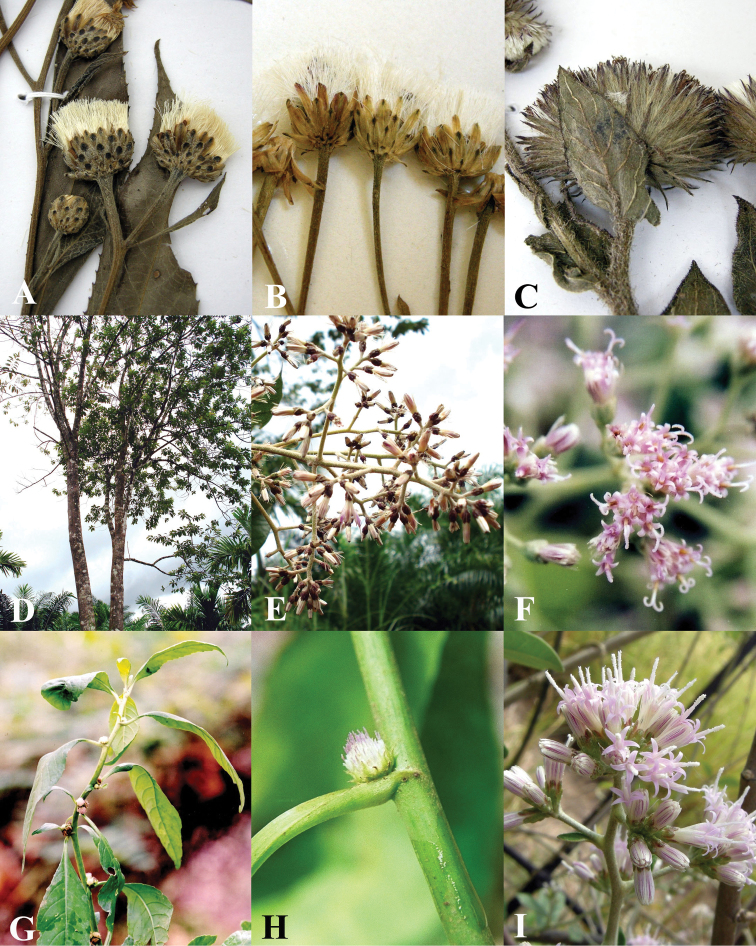
Morphology of *Vernonieae* in Thailand 6. **A** *Okia birmanica* **B** *Okia pseudobirmanica* **C** *Pulicarioidea annamica* **D–E** *Strobocalyx arborea* **F** *Strobocalyx solanifolia* **G–H** *Struchium sparganophorum* **I** *Tarlmounia elliptica*.

Myanmar, Moulmein, *O. Kuntze* 6271 (holotype: K!). Fig. 10A.

#### Description.

Annual herbs, 1–2 m tall. Stems erect, inconspicuously ribbed, puberulous. Leaves cauline, 5–20 by 1–5 cm, lanceolate, margin serrate, apex acuminate, base attenuate, chartaceous; upper surface scabrous, lower surface puberulous, lateral veins 8–12-paired; petioles up to 2 cm long. Capitulescences terminal, loosely paniculate. Capitula campanulate, 10–12 mm long, pedunculate. Receptacle convex, glabrous. Involucres subglobular, in 4–5 series, ca. 7 mm long. Phyllaries imbricate, green with purple apex, outer surface arachnoid; the outer and the middle ones ovate or elliptic, apex obtuse; the inner ones broadly oblong, apex obtuse. Florets ca. 55; corollas funnelform, white or purple, puberulous without glands. Achenes subterete, ca. 4 mm long, 10-ribbed, aculeate between ribs. Pappus in 2 series of bristles, the inner ones ca. 7 mm long, persistent.

#### Distribution.

Thailand: Lampang, Kanchanaburi. Myanmar.

#### Specimens examined.

Thailand, Kanchanaburi, Sai Yok national park, 14°22.29'N, 98°51.07'E, 4 Dec 1961, *K. Larsen* 8492 (K); Lampang, Jae Son national park, 4 Nov 1996, *J.F. Maxwell* 96-1490 (BKF).

#### Diagnostic characters.

*Okia birmanica* is distinguished by its long and slender pedicel in a loose panicle, phyllaries mostly obtuse with thickened tips, and cup-shaped involucre.

#### Ecology.

Under dense canopy on the top of limestone mountain, alt. 650–1000 m; flowering September to December.

#### Vernacular name.

Tum Doi (ตุ้มดอย).

### Okia pseudobirmanica

(H.Koyama) Bunwong, Chantar. & S.C.Keeley comb. nov.

urn:lsid:ipni.org:names:77138475-1

http://species-id.net/wiki/Okia_pseudobirmanica

Vernonia pseudobirmanica H.Koyama, Bull. Natl. Sci. Mus. Tokyo, Ser. B 29(1): 16. 2003.

#### Type.

Thailand, Tak, Khao Pha War, *T. Shimizu, H. Toyokuni, H. Koyama, T. Yahara & T. Santisuk* T-18505-bis (holotype: KYO!). Fig. 10B.

#### Description.

Perennial herbs, 20–40 cm tall. Stems erect, conspicuously ribbed, puberulous. Leaves cauline, 3–12 by 1–3 cm, elliptic or obovate, margin serrate, apex acuminate, base attenuate, chartaceous; upper surface glabrate or scabrous; lower surface glabrate or pubescent; lateral veins 7–12-paired; petioles up to 1 cm long. Capitulescences terminal, loosely corymbose. Capitula broadly campanulate, 5–6 mm long, pedunculate. Receptacle glabrous. Involucres broadly campanulate, in 4–5 series, ca. 5 mm long. Phyllaries imbricate, green, margin piliferous, outer surface nearly glabrous; the outer and the middle ones linear-oblong, apex acute; the inner ones ovate-lanceolate to oblong, apex obtuse. Florets ca. 20; corollas funnelform, purple, glabrous. Achenes fusiform, ca. 3 mm long, 10-ribbed, glabrous. Pappus in 2 series of bristles, the inner ones ca. 5 mm long.

#### Distribution.

Thailand: Tak, Kanchanaburi. Endemic.

#### Specimens examined.

Thailand, Tak, Khao Pha War, 16°46.32'N, 98°41.17'E, *T. Shimizu, H. Toyokuni, H. Koyama, T. Yahara & T. Santisuk* T-18505-bis (KYO); Kanchanaburi, Si Sa Wat, 13 Nov 1971, *C.F. van Beusekom, C. Phengklai, R. Geesink & B. Wongwan* 3746 (BKF, K, L, P).

#### Diagnostic characters.

*Okia pseudobirmanica* differs from *Okia birmanica* by its smaller capitula.

#### Ecology.

Rare on limestone, alt. 700–900 m; flowering November to December.

#### Vernacular name.

Tum Doi (ตุ้มดอย).

### Pseudelephantopus

Rohr, Skrifl. Nat. Selsk. Kiobenl. 2: 214. 1792 nom. et orth. cons.

urn:lsid:ipni.org:names:10701-1:1.1

http://species-id.net/wiki/Pseudelephantopus

#### Type.

*Pseudelephantopus spicatus* (Aubl.) C.F. Baker.

#### Description.

Perennial herbs. Stems erect, surface pilose-villose. Leaves simple, alternate or in rosette, sessile or petiolate; lamina obovate, oblanceolate, puberulous glandular; margin crenate, slightly serrate, dentate to entire; apex acute or obtuse, base cuneate to attenuate, chartaceous. Capitulescences terminal and axillary, spicate. Capitula discoid, tubular, clusters supported by foliaceous bracts, homogamous, florets bisexual and fertile. Phyllaries 8, in 2 series, decussate, persistent, oblong, outer surface puberulous. Florets 4; corollas white, glabrous, lobes 5, zygomorphic. Anthers 5, syngenesious, apical appendages acute, anther bases not calcarate. Styles white, 2-branched, inner surface covered with stigmatic papillae, outer surface covered with sweeping hairs reaching to below style bifurcation. Achenes usually clavate, 10-ribbed, pubescent, carpopodium present. Pappus with 2 unequal contorted bristles. Pollen lophate, 3-porate, without micropuncta.

One species is recognized in Thailand.

### Pseudelephantopus spicatus

(Juss. ex Aubl.) C.F. Baker, Trans. Acad. Sci. St. Louis 12: 45, 55 & 56. 1902.

urn:lsid:ipni.org:names:60438009-2:1.1.2.1

http://species-id.net/wiki/Pseudelephantopus_spicatus

Pseudelephantopus spicatus (Juss. ex Aubl.) Rohr, Skrifl. Nat. Selsk. Kiobenh. 2: 216. 1792.
Elephantopus spicatus Juss. ex Aublet, Hist. Pl. Guiane 2: 808. 1775.

#### Type.

not ascertained. Figs 8E–F.

#### Description.

Perennial herbs, 10–40 cm tall. Stems erect, inconspicuously ribbed, puberulous. Leaves simple, rosulate or alternate at base, 5–15 by 1.5–5 cm, obovate or oblanceolate, margin slightly serrate to entire, apex obtuse or rounded, base cuneate or attenuate, subcoriaceous; upper surface puberulous without glands, lower surface puberulous with filiform hairs and capitate glands; lateral veins 9–15-paired; petioles up to 2 cm long. Capitulescences terminal and axillary, capitula 1-4 aggregated in clusters supported by foliaceous bracts, clusters arranged in a spike. Capitula tubular, 14–17 mm long. Receptacle flat, 1–1.5 mm in diam., glabrous. Florets bisexual and fertile. Involucres oblong, in 2 series, 10–11 mm long. Phyllaries 8, decussate, light green, margin entire or piliferous, outer surface pilose without glands; the outer lanceolate, apicies acute; the inner ones obovate-lanceolate or oblong, apices acute. Florets 4; corollas salverform, white, zygomorphic, glabrous; corolla tubes 5–9 mm long; corolla lobes 2.5–2.8 mm long. Anthers 1.5–2 mm long, apical appendage acute, base acute. Styles white, 5–9 mm long, branches ca. 2 mm long, inner surface covered with stigmatic papillae. Achenes clavate, 4–5 mm long, pubescent with densely twin hairs, without glands, 10-ribbed. Pappus in 1 series, often of 2 sizes and bent at the tip, bristles 6–9, 2–6 mm long.

#### Distribution.

Thailand: Chiang Rai, Nakhon Phanom, Ubon Ratchathani. Tropics.

#### Specimens examined.

Thailand, Ubon Ratchathani, Chong Mek border crossing, 15°8.02'N, 105°28.01'E, 27 Oct 2007, *S. Bunwong* 342 (KKU); Chiang Rai, Muang District, 20 Nov 2007, *S. Bunwong* 352 (KKU, US); Nakhon Phanom, Ban Phang, 24 Feb 2003, *Th. Wongprasert et al.* 032-17 (BKF).

#### Diagnostic characters.

*Pseudelephantopus spicatus* is distinguished from *Elephantopus* by having spicate capitulescences and contorted pappus.

#### Ecology.

Open areas in dipterocarp forest or river bank, alt. 100–400 m; flowering October to December.

#### Vernacular name.

Doo La Doo (โด่ลาโด่), Ton Tai Din (ต้นใต้ดิน).

### Pulicarioidea

Bunwong, Chantar. & S.C.Keeley gen. nov.

urn:lsid:ipni.org:names:77138472-1

http://species-id.net/wiki/Pulicarioidea

#### Type.

*Pulicaria annamica* Gagnep., Bull. Soc. Bot. France 68: 121. 1921.

#### Description.

Perennial herbs. Stems erect, conspicuously ribbed, villose. Leaves oblong or oblanceolate, margin subentire, apex acute or truncate, base cuneate, subcoriaceous; petioles sessile. Capitulescences terminal, solitary or few. Capitula hemispherical, pedunculate. Receptacle glabrous. Involucres hemispherical, phyllaries imbricate, green with purple apically. Florets ca. 70; corollas narrowly funnelform, purple, pubescent with hairs and glands. Anthers with apical appendage acute, base acute. Styles purple. Achenes subterete or oblong, 3–5 ribbed, pubescent with dense twin hairs without glands, carpopodium present. Pappus in 2 series of bristles, persistent, the outer ones are shorter than the inner ones. Pollen lophate, without micropuncta.

### Pulicarioidea annamica

(Gagnep.) Bunwong, Chantar. & S.C.Keeley comb. nov.

urn:lsid:ipni.org:names:77138476-1

http://species-id.net/wiki/Pulicarioidea_annamica

Pulicaria annamica Gagnep., Bull. Soc. Bot France. 68: 121. 1921.
Vernonia pulicarioides Gagnep., Fl. Indo-Chine 3: 482. 1924.

#### Type.

Vietnam, Annam, Da Lat plateau, Langbian mountain, *A. Chevalier* 30672 (P!, lectotype designated here). Fig. 10C.

#### Description.

Perennial herbs, 20–40 cm tall. Stems erect, conspicuously ribbed, villose. Leaves 5–10 by 1.5–2 cm, oblong or oblanceolate, margin subentire, apex acute or truncate, base cuneate, subcoriaceous; upper surface scabrous without glands; lower surface scabrous with whip-shaped hairs and capitate glands; lateral veins 4–8-paired; petioles sessile. Capitulescences terminal, solitary or few. Capitula hemispherical, 10–15 mm long, pedunculate. Receptacle glabrous. Involucres hemispherical, in 5–6 series, 10–15 mm long, 10–15 mm in diam., phyllaries imbricate, green with purple apically, margin piliferous, outer surface sericeous without glands; the outer and the middle ones lanceolate, apex acuminate; the inner ones lanceolate, apex acuminate or aristate. Florets ca. 70; corollas narrowly funnelform, purple, pubescent with hairs and glands; corolla tubes 6–7 mm long; corolla lobes 2–2.5 mm long. Anthers 3.5–4 mm long, apical appendage acute, base acute. Styles purple, 6–6.5 mm long, branches 2–2.5 mm long. Achenes subterete or oblong, 2–2.5 mm long, 3–5 ribbed, pubescent with dense twin hairs without glands, carpopodium present. Pappus in 2 series of bristles, persistent, the outer ones shorter than the inner ones, the inner ones 8–9 mm long. Pollen lophate, without micropuncta.

#### Distribution.

Thailand: Mae Hong Son, Chiang Mai. Myanmar, Laos, Vietnam.

#### Specimens examined.

Thailand, Chiang Mai, Doi Sutep Pui national park, 18°48.39'N, 98°54.90'E, 20 Oct 1999, *P. Suksathan* 1932 (QBG); Laos, *M. Poilane* 2038 (P); Laos, 4 Feb 1932, *M. Poilane* 20073 (P); Vietnam, Annam, Da Lat plateau, Langbian mountain, 10 Feb 1914, *A. Chevalier* 30672 (P); Langbian mountain, 15 Feb 1914, *A. Chevalier* 30847 (P); Langbian mountain, *Eberhardt* 1747 (P); Langbian mountain, 27 Oct 1920, *E. Evrard* 406 (E, P); Langbian mountain, 19 Jan 1924, *E. Evrard* 1057 (P); Langbian mountain, *E. Evrard* 1446 (P); Langbian mountain, 20 Feb 1952, *Schmid* 1237 (P).

#### Diagnostic characters.

This species is distinguished by having hemispherical capitula, sericeous phyllaries without glands, 3–5 ribed achenes, whitish flattened pappus and lophate pollen with small lacuna.

#### Ecology.

Evergreen and pine-oak forest, alt. 780–1600 m; flowering October to February.

#### Vernacular name.

Pha Ya Muang Doi (พญาม่วงดอย).

### Strobocalyx

Sch.Bip., Jahresber. Pollichia 18. 170. 1861.

urn:lsid:ipni.org:names:11179-1:1.2.1.2

http://species-id.net/wiki/Strobocalyx

#### Type.

*Strobocalyx arborea* (Buch.-Ham.) Sch.Bip., Jahresber. Pollichia 18: 171. 1861.

#### Description.

Perennial plants. Stems arborescent or scandent. Leaves simple, alternate, usually petiolate, lamina elliptic or oblong, pubescent with uniseriate or flagellate hairs, margin serrate or entire, apex acute or acuminate, base cuneate, coriaceous. Capitulescences terminal or axillary. Capitula discoid, homogamous, pedunculate or sessile, florets bisexual and fertile. Involucre campanulate, in 2–4 series, 2–4 mm long. Phyllaries imbricate, hairy without glands. Corollas purple to white, actinomorphic, lobes 5. Anthers 5, syngenesious. Styles purple, 2-branched, inner surface covered with stigmatic papillae, outer surface covered with sweeping hairs on the outer surface reaching below style bifurcation. Achenes turbinate, usually 10-ribbed, ca. 2 mm long, hairy with glands, carpopodium present. Pappus in 2 series of bristles, persistent, the outer ones are shorter than the inner ones. Pollen echinate, subechinolophate or echinolophate, 3-colporate, with micropuncta.

Two species are recognized in Thailand.

#### Key to the species

**Table:** 

1	Plants arborescent; capitulescences thyrsoid-paniculate; achenes 3–4-angled	*Strobocalyx arborea*
–	Plants scandent or shrubby; capitulescences corymbose; achenes 10 ribs	*Strobocalyx solanifolia*

### Strobocalyx arborea

(Buch.-Ham.) Sch.Bip., Jahresber. Pollichia 18: 171. 1861.

urn:lsid:ipni.org:names:251873-1:1.2.1.2

http://species-id.net/wiki/Strobocalyx_arborea

Conyza arborea Wall., List [Wallich] no. 2. nom. nud.
Vernonia arborea Buch.-Ham., Trans. Linn. Soc. 14: 218. 1825.
Strobocalyx arborea Type. Nepal, S.N. (holotype: E!). Figs 10D–E.
Eupatorium javanicum Blume, Bijdr. Fl. Ned. Ind. 15: 903. 1826.
Vernonia javanica (Blume) DC., Prodr. 5: 22. 1836.
Strobocalyx arborea Type. Indonesia. Java, Blume s.n. (holotype: L).

#### Description.

Trees, 5–20 m tall. Stems arborescent, terete, inconspicuously ribbed, branches ferruginuous pubescent. Leaves 8–20 by 4–10 cm, elliptic to oblong, margin entire, apex acuminate or caudate, base cuneate or oblique, coriaceous; both surfaces puberulous with filiform hairs and capitate glands; lateral veins 10–15-paired; petioles up to 3 cm long. Capitulescences terminal or axillary, thyrsoid paniculate. Capitula narrowly campanulate, shortly pedunculate. Receptacle flat, glabrous. Involucres narrowly campanulate or slightly oblong-cylindrical, in 3–4 series, 2–3 mm long. Phyllaries imbricate, green or purple, margin piliferous, outer surface puberulous without glands; the outer ovate, apex obtuse or rounded; the inner ones ovate-lanceolate or oblong, apex obtuse. Florets 3–6; corollas funnelform, purple to white, glandular; corolla tubes 6–7 mm long; corolla lobes ca. 2 mm long. Anthers ca. 2.5 mm long, apical appendage acute, base obtuse. Styles purple. Achenes turbinate, ca. 2 mm long, 3–4-angled and inconspicuously ribbed, pubescent with twin hairs and capitate glands. Pappus in 2 series of bristles, the inner ones 6–7 mm long. Pollen subechinolophate, 3-colporate, with micropuncta.

#### Distribution.

Thailand: Nan, Loei, Ranong, Surat Thani, Phangnga, Phuket, Krabi, Nakhon Si Thammarat, Phatthalung, Trang, Satun, Songkhla, Pattani, Yala, Narathiwat. India, Nepal, Sri Lanka, Myanmar, Malay Peninsula, Vietnam, New Guinea.

#### Specimens examined.

Thailand, Phang Nga, Takuapha, 8°56'N, 98°21'E, 7 Jul 1972, *K. Larsen, S.S. Larsen, I. Nielsen & T. Santisuk* 30935 (AAU, BKF, K, L); Songkhla, Ban Prakamp, 18 Feb 1928, *A.F.G. Kerr* 15847 (BK, BM, K); Trang, Khao Chong, 11 Aug 1975, *J.F. Maxwell* 75-739 (AAU, BK, L); Yala, 29 Jan 1931, *Put* 3655 (BK, BM, E, K); Narathiwat, Nakorn Vang, 6 Oct 1966, *Prayad* 494 (BK, US); Nakhon Si Thammarat, Thung Song, 24 Jul 1929, *Rabil* 188 (BK, BM, E, K); Ranong, Ka Pur, 7 Dec 1979, *T. Shimizu, H. Toyokuni, H. Koyama, T. Yahama & C. Niyomdham* T-26360 (BKF, L); Ka Pur, 12 Aug 1973, *Pochanart* 427 (BKF, K, P); Ka Pur, 1 Jan 1919, *A.F.G. Kerr* 16477 (BK, BM, K); Surat Thani, Kanchanadid, 31 Jul 1927, *A.F.G. Kerr* 13034 (AAU, BK, BM, K, L).

#### Diagnostic characters.

*Strobocalyx arborea* is distinguished by its large size, 3–4-angled achenes and obtuse phyllaries.

#### Ecology.

Evergreen forest, alt. 50–300 m; flowering July to February.

#### Vernacular name.

Ka Ton Rok (กะท้อนรอก), Ka Puam Ma Prao (กะพวมมะพร้าว), Kra Phee Kao (กระพี้ขาว), Ko Ta Ba Ru (โกตาบารู), Kee Aon (ขี้อ้น), Torn Lor (ตอนเลาะ), Baa Hor (แบหอ), Smong Kung (สมองกุ้ง), Ai Nieaw Maa (อ้ายเหนียวหมา), Ta Kuam (ตะกวม), Nuang Chang (งวงช้าง).

### Strobocalyx solanifolia

(Benth.) Sch.Bip., Jahresber. Pollichia 18–19: 171. 181.

urn:lsid:ipni.org:names:251885-1:1.2.1.2

http://species-id.net/wiki/Strobocalyx_solanifolia

Vernonia solanifolia Benth., Lond. Journ. Bot. 1: 486. 1842.

#### Type:

Hong Kong: *Hinds* s.n. (holotype: K!). Fig. 10F.

#### Description.

Scandent or climbing shrubs, 2–10 m tall. Stems caulescent, becoming woody with age, young branches inconspicuously ribbed, ferruginous tomentose. Leaves 8–20 by 4–10 cm, ovate or elliptic, margin serrate or entire, apex acute or acuminate, base cuneate, subcoriaceous; upper surface puberulous without glands; lower surface tomentose with filiform hairs, flagellate hairs and capitate glands; lateral veins 5–7-paired; petioles up to 3.5 cm long. Capitulescences terminal and axillary, thyrsoid paniculate. Capitula narrowly campanulate, 8–10 mm long, pedunculate. Receptacle flat, 2–2.5 mm in diam., hairy. Involucres narrowly campanulate or slightly cylindrical, 2–3 series, 3.5–4 mm long, 3–4 mm in diam. Phyllaries imbricate, light green, margin piliferous, outer surface tomentose without glands; the outer and the middle ones ovate, apex obtuse; the inner ones obovate, apex obtuse. Florets 5–7; corollas funnelform, purple, puberulous, glands capitate; corolla tubes 4.5–6 mm long; corolla lobes 1.5–2.5 mm long. Anthers 2–2.5 mm long, apical appendage acute, base acute. Styles purple, 5–6.5 mm long, branches 2–2.5 mm long. Achenes turbinate, ca. 2 mm long, 10-ribbed, covered with sparse hairs and capitate glands. Pappus in 2 series of bristles, the inner ones 5–6 mm long. Pollen echinate, 3-colporate, with prominent micropuncta.

#### Distribution.

Thailand: Mae Hong Son, Chiang Mai, Nan, Lampang, Phitsanulok, Phetchabun, Loei, Sakon Nakhon, Chaiyaphum, Nakhon Ratchasima, Kanchanaburi, Nakhon Nayok. Hong Kong, Myanmar, Vietnam, Laos, Myanmar.

#### Specimens examined.

Thailand, Loei, Phu Kradung national park, 16°52.25'N, 101°50.74'E, *S. Bunwong* 68 (KKU); Mae Hong Son, Khum Yuam, 8 Apr 1977, *B. Nimanong & S. Phusomsaeng* 1813 (BKF, PSU); Lampang, Metud, 1 Mar 1925, *Winit* 1262 (BK, BKF, K); Phitsanulok, Thung Salang Luang, 22 Apr 1964, *Pradit* 846 (BK); Phetchabun, Lom Kao, 5 May 1955, *T. Smitinand* 2639 (BKF); Loei, Phu Kra Dung, 12 Feb 1931, *A.F.G. Kerr* 20129 (BK, BM, K, L); Dan Sai, 26 Mar 1965, *A.F.G. Kerr* 8816 (BK, BM, E, K); Phu Rue, 5 Mar 1993, *P. Chantaranothai, J. Parnell, D. Middleton & D. Simpson* 1079 (BKF); Chaiyaphum,Paa Hin Ngam, 22 Feb 1963, *Adisai* 382 (BK); Khao Kiew, 23 Feb 1931, *A.F.G. Kerr* 20226 (BK, BM, K); Khao Kiew, 6 Mar 1984, *W. Nanakorn* 391 (BKF); Nakhon Ratchasima; Kanchanaburi, Ban Tun, 2 Mar 1921, *A.F.G. Kerr* 4982 (BK, BM, K).

#### Diagnostic characters.

*Strobocalyx solanifolia* is distinguished by its scandent habit, corymbose capitulescences and tomentose leaf surfaces.

#### Ecology.

Hill evergreen or pine-oak forest, alt. 900–1250 m; flowering February to May.

#### Vernacular name.

Cha Kua (ชะเคือ จ๊าเขือ).

### Struchium

P.Browne, Civ. Nat. Hist. Jamaica 312, t. 34. 1756.

urn:lsid:ipni.org:names:11185-1:1.3

http://species-id.net/wiki/Struchium

#### Type.

*Struchium herbaceum* J. St.-Hil.

#### Description.

Annual herbs. Stems caulescent, erect or decumbent, puberulous. Leaves simple, alternate, usually petiolate, lamina elliptic, pubescent, margin serrate, apex acute, base attenuate, chartaceous. Capitulescences axillary, in clusters. Capitula discoid, homogamous, hemispherical, sessile, florets bisexual and fertile. Involucre imbricate. Florets 50–70; corollas purple to white, actinomorphic, glandular, corolla lobes 3–4. Anthers 3 or 4, syngenesious. Styles purple, 2-branched, inner surface covered with stigmatic papillae, outer surface covered with sweeping hairs reaching to below style bifurcation. Achenes turbinate, 3–4-angular, usually 3–5-ribbed, carpopodium present. Pappus coroniform, thick, in 1 series, persistent. Pollen lophate, 3-porate, without micropuncta.

One species is recognized in Thailand.

### Struchium sparganophorum

(L.) Kuntze, Revis. Gen. Pl. 1: 366. 1891.

urn:lsid:ipni.org:names:251901-1:1.1.2.1.1.3

http://species-id.net/wiki/Struchium_sparganophorum

Ethulia sparganophora L., Sp. Pl.: 1171. 1763.
Sparganophoros vaillantii Crantz, Inst. Rei. Herb. 1: 261. 1766.
Ethulia struchium Sw., Prodr. 111. 1788.

#### Type.

Jamaica (not seen). Figs 10G–H.

#### Description.

Annual, 20–50 cm tall. Stems erect, inconspicuously ribbed, puberulous. Leaves 4–12 by 2–15 cm, elliptic, pubescent, margin serrate, apex acute, base attenuate, chartaceous; both surfaces puberulous with cylindrical hairs and capitate glands; lateral veins 7–11-paired; petioles up to 12 mm long. Capitulescences axillary, solitary or clustered. Capitula hemispherical, sessile, 4–6 mm in diam. Receptacle convex, 2–2.5 mm in diam., glabrous. Involucres 3–4 series, 3–4 mm long, imbricate, hemispherical. Phyllaries light green, margin piliferous, outer surface puberulous without glands; the outer ovate to lanceolate, apex acute to acuminate; the inner ones obovate-lanceolate, apex acuminate. Florets 50–70; corollas funnelform, white, glandular; corolla tubes 1–1.5 mm long; corolla lobes 3–4, 0.5–1 mm long. Anthers ca. 1 mm long, apical appendage acute, base acute. Styles purple, ca. 2 mm long, branches 1–1.5 mm long, inner surface covered with stigmatic papillae. Achenes turbinate, 3–4-angular, 1–1.5 mm long, 3–5-ribbed, glandular. Pappus of 3–4 parts, coroniform, ca. 1 mm long, whitish.

#### Distribution.

Thailand: Lamphun, Nakhon Phanom, Kanchanaburi, Bangkok, Ranong, Phangnga, Krabi, Trang, Songkhla, Yala. Tropics.

#### Specimens examined.

Thailand, Phang Nga, Muang district, 8°26.55'N, 98°31.16'E, 1 Aug 2002, *S. Bunwong* 28 (KKU); Lamphun, Muang District, 20 Dec 1994, *P. Palee* 265 (BKF, CMU, L); Bangkok, Klong San, 20 Feb 1971, *J.F. Maxwell* 71-107 (AAU, BK, L); Lad Yao, 27 Sep 1984, *Y. Paisooksantivatana* 1433-84 (BK); Ranong, Kra Buri, 10 Apr 1967, *C. Chermsirivathana* 1266 (BK, L); Kra Buri, *S. Sutheesorn* 2275 (BK); Ka Pur, 7 Dec 1979, *T. Shimizu, H. Toyokuni, H. Koyama, T. Yahama & C. Niyomdham* 26302 (AAU, BKF, L); Trang, Khao Chong, 13 Aug 1975, *J.F. Maxwell* 75-824 (AAU, BK, L); Songkhla, Rattaphume, 3 Aug 1986, *J.F. Maxwell* 86-636 (AUU, BKF, L); Rattaphume, *J.F. Maxwell* 86-911 (BKF, L).

#### Diagnostic characters.

Distinct characters of *Struchium sparganophorum* are the sessile capitula in axillary head, achenes with coroniform pappus, and florets with 3–4 corolla lobes.

#### Ecology.

Open sandy grassland or secondary evergreen forest, alt. 50–400 m; flowering August to April.

#### Vernacular name.

Muk Din (มุกดิน).

### Tarlmounia

H.Rob., S.C.Keeley, Skvarla & R.Chan, Proc. Biol. Soc. Washington 121(1): 31. 2008.

urn:lsid:ipni.org:names:77094699-1:1.1

http://species-id.net/wiki/Tarlmounia

#### Type.

*Vernonia elliptica* DC. in Wight, Contrib. Bot. Ind. 5. 1834.

#### Description.

Perennial plants. Stems scandent, young branches terete, white, sericeous. Leaves simple, alternate, petiolate, sericeous with horn-shaped hairs, lamina elliptic, margin entire or serrate, apex acute rounded, base rounded, subcoriaceous. Capitulescences terminal or axillary. Capitula discoid, homogamous, pedunculate, florets bisexual and fertile. Involucres imbricate, in 3–4 series, 3–4 mm long, glandular without hairs. Corollas purple to white, actinomorphic, corolla lobes 5. Anthers 5, syngenesious. Styles 2-branched, inner surface covered with stigmatic papillae, outer surface covered with sweeping hairs on the outer surface reaching below style bifurcation. Achenes turbinate, 4–7-ribbed, ca. 2 mm long, glandular without hair, carpopodium present. Pappus in 2 series of bristles, persistent, the outer ones are shorter than the inner ones. Pollen echinate, 3-colporate, with micropuncta.

One species is recognized in Thailand.

### Tarlmounia elliptica

(DC.) H.Rob., S.C.Keeley, Skvarla & R.Chan, Proc. Biol. Soc. Washington 121(1): 32. 2008.

urn:lsid:ipni.org:names:77094700-1:1.1

http://species-id.net/wiki/Tarlmounia_elliptica

Vernonia elliptica DC. in Wight, Contrib. Bot. Ind. 5. 1834.

#### Type.

India, Nilgherry, *Wight* 1377 (holotype: E!). Fig. 10I.

#### Description.

Climbing shrubs or scandents. Stems caulescent, young branches inconspicuously ribbed, white sericeous. Leaves 5–12 by 3–6 cm, elliptic, margin entire or serrate, apex acute or obtuse, base rounded, subcoriaceous; upper surface puberulous without glands; lower surface sericeous with T-shaped hairs; lateral veins 7–11-paired; petioles up to 1 cm long. Capitulescences terminal and axillary, thyrsoid paniculate. Capitula narrowly campanulate, 10–15 mm long. Receptacle convex, ca. 1 mm in diam., glabrous. Involucres narrowly campanulate or slightly oblong-cylindrical, in 3–4 series, 3–4 mm long, ca. 3 mm in diam. Phyllaries imbricate, green or purple apically, margin piliferous, outer surface arachnoid glandular; the outer and the middle ones ovate, obtuse to rounded; the inner ones obovate, apex acute or obtuse. Florets 4–5; corollas funnelform, purple or white, glandular, corolla tubes 4.5–5.5 mm long; corolla lobes 2–3 mm long. Anthers 3–3.5 mm long, apical appendage acute, base obtuse. Styles purple, 5.5–7 mm long, branches ca. 2 mm long. Achenes turbinate, ca. 2 mm long, 4–7-ribbed, covered with sparse hairs and capitate glands. Pappus in 2 series of bristles, the inner ones 5–6 mm long, persistent.

#### Distribution.

Thailand: Mae Hong Son, Chiang Mai, Phitsanulok, Loei, Nong Bua Lum Phu, Udon Thani, Chaiyaphum, Mahasarakam, Nakhon Ratchasima, Roi Et, Si Sa Ket, Phetchaburi, Saraburi, Phra Nakhon Si Ayutthaya, Nakhon Nayok, Nonthaburi, Bangkok, Prachin Buri, Chon Buri, Rayong, Chanthaburi, Trat, Chumphon, Surat Thani, Nakhon Si Thammarat. America, Africa, Asia.

#### Specimens examined.

Thailand, Mahasarakam, Nadoon district, 15°46.22'N, 103°1.81'E, 1 Mar 2003, *S. Bunwong* 69 (KKU); Khon Kaen, Chumpare District, 23 Feb 2008, *S. Bunwong* 390 (KKU, US); Phitsanulok, Thung Salang Luang, 1 Mar 1971, *S. Sutheesorn* 2980 (BK); Loei, Paa See Than, 15 Feb 1901, *P. Suvarnakoset* 1300 (BKF, US); Chaiyaphum, 27 Jan 1931, *A.F.G. Kerr* 19950 (BK, BM, E, K); Ratchaburi, Tapha Ban Pang, 25 Feb 1965, *S. Sutheesorn* 371 (BK, US); Saraburi, Muang District, 22 Feb 1975, *J.F. Maxwell* 75-152 (AAU, BK, L); Muang District, 19 Mar 1990, *Dee* 34 (BKF); Nakhon Nayok, 16 Feb 1966, *K. Iwatzuki & N. Fukuoka* T-7373 (BKF); Nakhon Nayok, 16 Jan 1985, *F. Konta, W. Nanakorn & Th. Wongprasert* 49085 (BKF, K, L); Bangkok, Ram Intra, 21 Feb 1983, *H. Koyama & H. Terao* T-33716 (BKF); Prachin Buri, Aranyaprathet, 29 Mar 1962, *Adisai* 123 (BK); Chon Buri, Bang Saan, 18 Oct 1982, *Y. Paisooksantivatana* 1182-82 (BK); Trat, Koh Chang, 19 Feb 1998, *Sanan* 13 (E, BKF, US); Koh Chang, 15 Jan 1955, *B. Sangkhachand* 417 (BKF, US); Rayong, Ban Pe, 24 Feb 1980, *Put* 2771 (BK, BM, E, K); Chantaburi, Khao Soi Dow, 10 Feb 1966, *K. Iwatzuki & N. Fukuoka* T-7314 (BKF); Chumphon, 1 Feb 1968, *Vacharapong* 8 (BK, US); Surat Thani, Ta Kanan, 16 Mar 1927, *A.F.G. Kerr* 12344 (BK, BM, K); Nakhon Si Thammarat, Ka Biad, 15 Mar 1957, *Sanan* 1002 (BKF, US).

#### Diagnostic characters.

*Tarlmounia elliptica* is characterized by having appressed T-shaped hairs on leaf surfaces, scandent habits and involucre without glands.

#### Ecology.

Open area in wetland or saline land, alt. 0–100 m; flowering October to May.

#### Vernacular name.

Kiew Darn (เขี้ยวดาน), Sar Muk Lord (ซ้าหมักหลอด), Tanmon (ตานหม่อน), Tao Kee Tao (เถาขี้เถ้า), Lee Kuan Yuu (ลีกวนยู), Tao Wan Lek (เถาวัลย์เหล็ก), Khud Mon (คัดมอญ), Tarlmoun (ตาลหม่น).

## Supplementary Material

XML Treatment for Vernonieae

XML Treatment for Acilepis

XML Treatment for Acilepis attenuata

XML Treatment for Acilepis chiangdaoensis

XML Treatment for Acilepis divergens

XML Treatment for Acilepis doichangensis

XML Treatment for Acilepis kerrii

XML Treatment for Acilepis kingii

XML Treatment for Acilepis namnaoensis

XML Treatment for Acilepis ngaoensis

XML Treatment for Acilepis peguensis

XML Treatment for Acilepis principis

XML Treatment for Acilepis pseudosutepensis

XML Treatment for Acilepis saligna

XML Treatment for Acilepis silhetensis

XML Treatment for Acilepis squarrosa

XML Treatment for Acilepis sutepensis

XML Treatment for Acilepis tonkinensis

XML Treatment for Acilepis virgata

XML Treatment for Camchaya

XML Treatment for Camchaya gracilis

XML Treatment for Camchaya kampotensis

XML Treatment for Camchaya loloana

XML Treatment for Camchaya loloana var. loloana

XML Treatment for Camchaya loloana var. mukdahanensis

XML Treatment for Camchaya pentagona

XML Treatment for Camchaya spinulifera

XML Treatment for Camchaya tenuiflora

XML Treatment for Camchaya thailandica

XML Treatment for Cyanthillium

XML Treatment for Cyanthillium cinereum

XML Treatment for Cyanthillium montanum

XML Treatment for Cyanthillium patulum

XML Treatment for Decaneuropsis

XML Treatment for Decaneuropsis cumingiana

XML Treatment for Decaneuropsis eberhardtii

XML Treatment for Decaneuropsis garrettiana

XML Treatment for Elephantopus

XML Treatment for Elephantopus mollis

XML Treatment for Elephantopus scaber

XML Treatment for Elephantopus scaber var. penicillatus

XML Treatment for Elephantopus scaber var. scaber

XML Treatment for Ethulia

XML Treatment for Ethulia conyzoides

XML Treatment for Gymnanthemum

XML Treatment for Gymnanthemum extensum

XML Treatment for Iodocephalopsis

XML Treatment for Iodocephalopsis eberhardtii

XML Treatment for Koyamasia

XML Treatment for Koyamasia calcarea

XML Treatment for Koyamasia curtisii

XML Treatment for Koyamasia curtisii var. curtisii

XML Treatment for Koyamasia curtisii var. tomentosa

XML Treatment for Kurziella

XML Treatment for Kurziella gymnoclada

XML Treatment for Monosis

XML Treatment for Monosis parishii

XML Treatment for Monosis volkameriifolia

XML Treatment for Okia

XML Treatment for Okia birmanica

XML Treatment for Okia pseudobirmanica

XML Treatment for Pseudelephantopus

XML Treatment for Pseudelephantopus spicatus

XML Treatment for Pulicarioidea

XML Treatment for Pulicarioidea annamica

XML Treatment for Strobocalyx

XML Treatment for Strobocalyx arborea

XML Treatment for Strobocalyx solanifolia

XML Treatment for Struchium

XML Treatment for Struchium sparganophorum

XML Treatment for Tarlmounia

XML Treatment for Tarlmounia elliptica

## References

[B1] Bunwong S Chantaranothai P (2008) Pollen morphology of the Tribe Vernonieae (Compositae) in Thailand. The Natural History Journal of Chulalongkorn University 8(1): 45-55

[B2] Bunwong S Robinson H Chantaranothai P (2009) Taxonomic notes on *Camchaya* and *Iodocephalus* (Vernonieae: Asteraceae), and a new genus *Iodocephalopsis*. Proceedings of the Biological Society of Washington 122: 357-363. doi: 10.2988/08-45.1

[B3] Keeley SC Forsman ZH Chan R (2007) A phylogeny of the ‘‘evil tribe’’ (Vernonieae: Compositae) reveals Old/New World long distance dispersal: Support from separate and combined congruent datasets (trnL-F, ndhF, ITS). Molecular Phylogenetics and Evolution 44: 89-103. doi: 10.1016/j.ympev.2006.12.024 17292633 10.1016/j.ympev.2006.12.024

[B4] Keeley SC Robinson H (2009) Vernonieae. In: Funk VA Susanna A Stuessy TF Bayer RJ (Eds) Systematics, Evolution, and Biogeography of Compositae.IAPT, Vienna: 439-461

[B5] Kerr AFG (1936) *Vernonia* Schreb. Flora Siamensis Enumeratio 2(3): 236-245

[B6] Koyama H (1984) Taxonomic Studies in the Compositae of Thailand 3. Acta Phytotaxonomica Geobotanica 35(1–3): 49-58

[B7] Koyama H (1993) Taxonomic studies in the Compositae of Thailand 10. *Vernonia* Schreb. sect. *Decaneurum* (DC.) Oliv. Acta Phytotaxonomica Geobotanica 44(1): 29-34

[B8] Koyama H (1997) Taxonomic studies in the Compositae of Thailand 11. *Vernonia* Schreb. sect. *Strobocalyx* Bl. Bulletin of the Science Museum Series B (Botany) 23(4): 159-166

[B9] Koyama H (1998) Taxonomic studies in the Compositae of Thailand 12. *Vernonia* Schreb. sect. *Tephrodes* DC. and sect. *Cyanopis* Bl. Bulletin of the Science Museum Series B (Botany) 24(3): 109-115

[B10] Koyama H (2003) Taxonomic studies in the Compositae of Thailand 15. *Vernonia* sect. *Calcarea* comb. nov. Bulletin of the Science Museum Series B (Botany) 29(1): 15-22

[B11] Koyama H (2004) Taxonomic studies in the Compositae of Thailand 16. *Vernonia* sect. *Xipholepis* and *Claotrachelus*. Bulletin of the Science Museum Series B (Botany) 30(1): 21-34

[B12] Koyama H (2005) Taxonomic studies in the Compositae of Thailand 17. *Vernonia* sect. *Lepidaploa* subsect. *Paniculatae*. Bulletin of the Science Museum Series B (Botany) 31(2): 67-78

[B13] Maddison WP Maddison DR (2001) MacClade, analysis of phylogeny and character evolution, version 4.03. Sinauer Associates, Sunderland

[B14] Narayana BM (1979) Taxonomic value of trichomes in *Vernonia* Schreb. (Asteraceae). Proceedings of the Indian Academy of Sciences 88B(5): 347–357

[B15] Robinson H (1999a) Generic and subtribal classification of American Vernonieae. Smithsonian Contributions to Botany 89: 1-116. doi: 10.5479/si.0081024X.89

[B16] Robinson H (1999b) Revision of paleotropical Vernonieae (Asteraceae). Proceedings of the Biological Society of Washington 112: 220-247

[B17] Robinson H (2007) Vernonieae. In: Kadereit JW Jeffrey C (Eds) The Families and Genera of Vascular Plants, vol. 8, Flowering Plants, Eudicots, Asterales Springer, Berlin, 149-174

[B18] Robinson H Bunwong S Chantaranothai P (2010) A new genus, *Kurziella*, from Thailand (Vernonieae: Asteraceae). Proceedings of the Biological Society of Washington. 123: 174-178. doi: 10.2988/10-01.1

[B19] Robinson H Skvarla JJ (2006) Studies on the Gymnantheminae (Vernonieae: Asteraceae): restoration of the genus *Monosis*. Proceedings of the Biological Society of Washington 119: 600-607. doi: 10.2988/0006-324X(2006)119[600:SOTGVA]2.0.CO;2

[B20] Robinson H Skvarla JJ (2007) Studies on the Gymnantheminae (Vernonieae: Asteraceae) II: A new genus, *Decaneuropsis*, from China, India, and southeast Asia. Proceedings of the Biological Society of Washington 120: 359-366. doi: 10.2988/0006-324X(2007)120[359:SOTGAV]2.0.CO;2

[B21] Robinson H Skvarla JJ (2009) Study on the Paeotropical Vernonieae (Asteraceae): addition to the genus *Acilepis* from southern Asia. Proceedings of the Biological Society of Washington 122: 131-145. doi: 10.2988/08-19.1

[B22] Robinson H Keeley SC Skvarla JJ Chan R (2008) Studies on the Gymnantheminae (Vernonieae: Asteraceae) III: Restoration of the genus *Strobocalyx* and the genus *Tarlmounia*. Proceedings of the Biological Society of Washington 121: 19-33

[B23] Suvatti C (1978) Flora of Thailand, vol. 2 Kurusapha Publishing, Thailand

[B24] Swofford DL (2002) PAUP*: Phylogenetic analysis using parsimony (* and other methods), version 4.0b10. Sinauer Press, Sunderland, Massachusetts

